# Revision of the genus *Cenophengus* LeConte, 1881 (Coleoptera, Phengodidae), with the description of four new species, new geographic records and a new synonymy

**DOI:** 10.3897/zookeys.1068.70295

**Published:** 2021-11-04

**Authors:** Viridiana Vega-Badillo, Juan J. Morrone, Santiago Zaragoza-Caballero

**Affiliations:** 1 Laboratorio de Entomología, Departamento de Zoología, Instituto de Biología, Universidad Nacional Autónoma de México (UNAM), 04510 Mexico City, Mexico Universidad Nacional Autónoma de México Mexico City Mexico; 2 Posgrado en Ciencias Biológicas, Universidad Nacional Autónoma de México (UNAM), 04510 Mexico City, Mexico Universidad Nacional Autónoma de México Mexico City Mexico; 3 Museo de Zoología “Alfonso L. Herrera”, Departamento de Biología Evolutiva, Facultad de Ciencias, Universidad Nacional Autónoma de México (UNAM), 04510 Mexico City, Mexico Universidad Nacional Autónoma de México Mexico City Mexico

**Keywords:** Diversity, Nearctic and Neotropical regions, taxonomy

## Abstract

A taxonomic revision of the genus *Cenophengus* LeConte, 1881 (Coleoptera: Phengodidae) is provided, including new data on geographic ranges of the species. This is the first time this genus has been recorded for Belize and in Honduras. Four new species (*C.gardunoi*, *C.saasil*, *C.tsiik* and *C.zuritai*) are described and a new synonymy (*C.guerrerensis*, Zaragoza-Caballero, 1991 = *C.major* Wittmer, 1976) is established. The study includes a key to the 30 valid species, diagnoses, descriptions, photographs and distribution maps.

## Introduction

The genus *Cenophengus* was described by LeConte (1881), based on *C.debilis*, a species from California, United States of America. LeConte took into account characters such as the shape of the maxillary palpi, the antennal length and the shape of the seventh and eighth abdominal segments. He also considered the prothorax to be a little longer than wide, with a lateral border present only behind the mid-length and the anterior angle of the pronotum acute. Schaeffer (1904) described *C.pallidus*, from Texas, but stressed that it did not agree completely with LeConte’s description of the genus. Wittmer (1963) removed three species from this genus and transferred them to *Paraptorthodius* (*C.mirabilis* [Schaeffer 1904]) and *Phrixothix* (*C.nanus* [Wittmer 1948] and *C.unicolor* [Pic, 1926]) and described a new species for *Cenophengus* (*C.penai*). Wittmer (1976) added some characters to the description of *Cenophengus* (mandibles simple, maxillary palpi with four palpomeres, labial palpi with two palpomeres, head with two separated tentorial pits and gula with two sutures) and described one species from Colombia and five more from Mexico. He also transferred *C.unicolor* to *Oxymastinocerus*; and *C.penai* and *C.nanus* to a new genus (*Neophengus*) with reserves in *C.nanus* (as the front is too wide, this does not match with the description of *Neophengus*). Finally, he described one species of *Cenophengus* from Costa Rica and another from the United States (Wittmer 1981, 1986).

**Figure 1.**
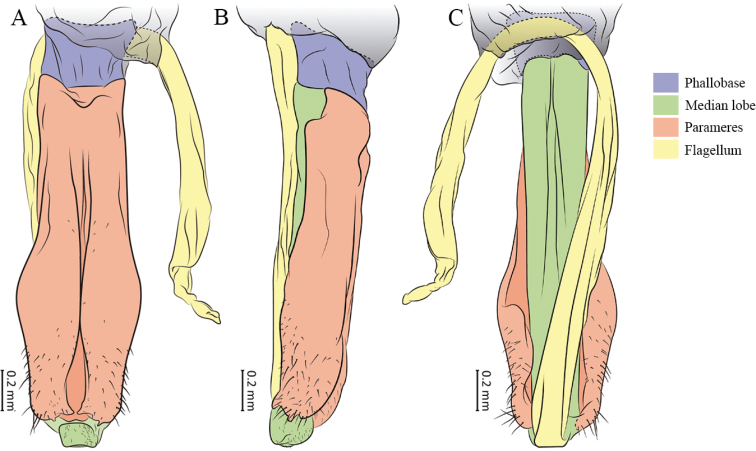
Morphological structures in the male reproductive apparatus of *Cenophengusgardunoi* sp. nov. **A** dorsal view **B** lateral view **C** ventral view.

Zaragoza-Caballero (1975, 1984, 1986, 1988, 1991, 2003, 2008) described 12 Mexican species, with his descriptions more detailed than previous ones. Vega Badillo et al. (2020) designated *C.breviplumatus* as the type species of a new genus (*Cleicosta*), characterised by short elytra, the last seven tergites exposed, gular sutures parallel anteriorly and parameres of aedeagus narrowed medially to spineless apex, differing from *Cenophengus* in that the latter has the parameres of the aedeagus parallel, with apical teeth (Fig. 1). Finally, Vega Badillo et al. (2021a) described one species from Guatemala and five more for Mexico. Before this treatment, *Cenophengus* consisted of 27 species, known from the USA, Mexico, Guatemala and Costa Rica.

The aim of this study is to revise the species of *Cenophengus*, based on available type material and other specimens.

## Material and methods

A total of 85 specimens for the analysis were borrowed from the following collections (acronyms follow the Insect and Spider Collections of the World website [Evenhuis 2020]): **CNIN**, Colección Nacional de Insectos, Instituto de Biología, UNAM, Mexico City (Santiago Zaragoza Caballero); **CNCI**, The Canadian National Collection of Insects, Arachnids, and Nematodes, Canada (Patrice Bouchard); **NMNH**, Smithsonian Institution, Washington, DC, USA (Floyd Schokley); **FSCA**, Florida State Collection of Arthropods (Paul Skelley); **FMNH**, Field Museum of Natural History, Chicago, USA (Crystal A. Maier); **AMNH**, American Museum of Natural History, New York, USA (Lee Herman); **CBG**, Centre for Biodiversity Genomics, University of Guelph, Canada (Kate Perez). Twenty-three holotypes were personally examined and two holotypes (*C.debilis* and *C.niger*) were examined through photographs provided by **MCZ**, Museum of Comparative Zoology Collection Harvard University, Boston, USA and **NHMB**, Natural History Museum Basel, Switzerland (Matthias Borer), respectively. Two holotypes (*C.pallidus* and *C.magnus*) were not available for this study; however, in the case of *C.pallidus*, the literature was consulted (Schaeffer 1904) and specimens identified by Wittmer were examined. For *C.magnus*, in addition to the literature (Zaragoza-Caballero 1988), specimens identified by Zaragoza-Caballero were examined.

The material was determined by means of existing taxonomic keys (Wittmer 1976), species level identification was made from the original descriptions (Wittmer 1976; Zaragoza-Caballero1986, 1988, 1991, 2008) and reference material identified by Wittmer. The following measurements were taken with a Zeiss Discovery V8 stereoscopic microscope, equipped with a 1× lens and a 1.6× eyepiece (80×): measurements were taken in dorsal view for the following structures: body length, interantennal (between the antennal fossae) and interocular distance (across widest part), length and width at widest point of head, eye, pronotum, elytra, scape; antennomeres, antennal rami (along mid-line of structure); in the case of the following structures, measurements were taken in ventral view: maxillary and labial palpi and tarsomeres (along mid-line of structure). These measurements are provided in millimetres, including the range, followed by average and standard deviation and the sample size, for eight species only, the holotype measurements are presented. To describe the morphology of the hind wings of each species, the hind left wing was dissected from one to two specimens, mounted on cardboard sheets and placed on the mounting pins of the corresponding specimens. Original drawings were made with a Zeiss Discovery V8 stereoscopic microscope, equipped with camera lucida. Once separated, the aedeagi were mounted on cardboard points and placed on the mounting pins of the corresponding specimens.

The taxonomic treatment includes information on the type specimens and the material examined. In the Remarks section, we comment on the morphological similarities and dissimilarities between phylogenetically closed taxa (Vega-Badillo et al. 2021b). A key is provided to identify adults of *Cenophengus* to species. General terminology follows Lawrence et al. (2011), except for hind wing veins that follows Kukalova-Peck and Lawrence (1993). Photographs were taken with a Zeiss Axio Zoom V16 with a Plan NeoFluar Z lens, 1×10.25 FWD 56 in the Laboratorio de Microscopía y Fotografía de la Biodiversidad, Instituto de Biología, UNAM. Studied material is cited in the following format: labels of the specimens are arranged in sequence from top to bottom, where the data for each label are within double quotes (“”), a slash (/) separates the rows and information between square brackets ([]) provides the correct information for label mistakes. # (Number of specimens) | Depositories (acronym of collection where the specimens are deposited). The number between parentheses refers to the number of specimens in the lot.

## Results

### Cenophengus

Taxon classificationAnimaliaColeopteraPhengodidae

LeConte, 1881

2038E342-D78B-57D6-AB69-71749763B094

#### Type species.

*Cenophengusdebilis* LeConte, 1881

#### Diagnosis.

Body 2.8–16.0 mm long; interantennal distance less than or equal to the scape length; antennae with 12 antennomeres, antennomeres 4 to 11 each with two symmetrical rami, 1.5 to 3 times longer than the respective antennomere; mandibles long, thin and crossed; maxillary palpi 4-segmented, last segment securiform; labial palpi 2-segmented; two separated tentorial pits and gula with two sutures; pronotum longer than wide, anterior edge rounded, sides almost straight; each elytron 2.8 to 5.4 times longer than wide, leaving the last 3 abdominal segments exposed; tarsomeres simple, without ventral combs; claws simple, without any teeth; wing with radial cell closed, RP reaching half or length less than half of MP1+2; and aedeagus with parameres slightly widened towards the mid-length, apex toothed mesad.

#### Redescription.

**Male.** Body length 2.8–16.0 mm; maximum body width 0.44–2.0 mm (pronotum). ***Head*.** Surface of vertex concave, wider than long, with posterior margin posteriorly convergent, usually partially covered by pronotum, integument smooth (without microsculpture) or chagreened (with isodiametric microscuplture); antennae 2 to 3 times the pronotum; antennae with 12 antennomeres, antennomeres 4 to 11 each with two long, antennomeres 12 lanceolate; symmetrical antennal rami, 1.5 to 3 times longer than antennomere; eyes hemispherical, finely faceted, 1/2 to 3/4 as long as head in lateral view, laterally projected in dorsal view; surface of vertex survace slightly concave between eyes, with a declivity between antennae, interantennal distance less than or equal length of antennomere 1; clypeus bilobed, partially or totally sclerotised, wider than long; mandibles long and thin, projected and crossed, pointed forward obliquely from head; maxillary palpi with four palpomeres, palpomere 2, 0.5 to 1.5 times as long as 3, palpomere 3 twice as long as 1, palpomere 4 securiform, 3 times as long as 3, twice as long as wide; labial palpi with two palpomeres, short, palpomere 2, twice to 5 times as long as 1, not covered by mandibles, last palpomere spindle-shaped; two separated tentorial pits (in the middle of the head in ventral view) and gula with two sutures. ***Thorax*.** Pronotum as long as or longer than wide, integument smooth or chagreened, coarsely punctured, anterior margin convex in dorsal view, posterior edge convex with a small median notch or not, sides almost straight, anterior angles rounded, posterior angles rounded or acute; prosternal anterior margin sinuate; scutellum triangular, narrowed distally; each elytron 2.8 to 5.4 times wider than long, leaving the last 3 abdominal segments exposed, apex slightly swollen in dorsal view; hind wings with radial cell closed, r4 vein present or absent (if present: without touching the RP and the radial cell or touching the RP and the radial cell) , r3 vein present or absent, RP up to half as long as MP1+2, medial field may or may not contain seven main veins: MP3, MP4, CuA, CuA2, CuA3+4, AA1+2 and AA3+4; AA and AP3+4 well-marked to vestigial and cubito-anal cell open or closed; legs increasing in length posterad, tarsomeres simple without ventral combs, tarsomeres 1 and 2 of the prothoracic legs similar in length, tarsomere 3 shorter, tarsomeres 1, 2 and 3 of meso- and metathoracic legs decreasing in length, fourth tarsomere of all legs 1/3 as long as fifth; claws simple, without any teeth. ***Abdomen*.** Eight sternites visible, sternite 7 with sides subparallel, posterior margin sinuate; sternite 8 rhomboidal, with posterior margin notched; aedeagus with phallobase entirely sclerotised; median lobe cylindrical, rounded apically; flagellum not encircling median lobe at rest, about 1.5 times longer than median lobe; parameres symmetrical in dorsal view, slightly widened towards the middle, toothed on mesal side pre-apically, with long bristles separated by a distance at least 0.2 setae lengths.

**Table 1.** Species of the genus *Cenophengus*. Country of origin of the species. Holotype: acronym of the collections where the holotype is deposited.

Species	Author	Country	Holotype
*C.baios*	Zaragoza-Caballero 2003	Mexico	CNIN
*C.brunneus*	Wittmer 1976	Mexico	NMNH
*C.ciceroi*	Wittmer 1981	USA	NMNH
*C.cuicatlaensis*	Zaragoza-Caballero 2008	Mexico	CNIN
*C.debilis*	LeConte 1881	USA	MCZ
*C.gardunoi* sp. nov.	This work	Mexico	CNIN
*C.gorhami*	Zaragoza-Caballero 1986	Mexico	NMNH
*C.hnogamui*	Vega-Badillo et al. 2021	Mexico	CNIN
*C.howdeni*	Zaragoza-Caballero 1986	Mexico	CNIN
*C.hautulcoensis*	Zaragoza-Caballero 2008	Mexico	CNIN
*C.kikapu*	Vega-Badillo et al. 2021	Mexico	CNIN
*C.longicollis*	Wittmer 1976	USA and Mexico	FMNH
*C.magnus*	Zaragoza-Caballero 1988	Mexico	CUIC
*C.major*	Wittmer 1976	Mexico	AMNH
*C.marmoratus*	Wittmer 1976	Mexico	NMNH
*C.mboi*	Vega-Badillo et al. 2021	Mexico	CNIN
*C.mumui*	Vega-Badillo et al. 2021	Mexico	CNIN
*C.munizi*	Zaragoza-Caballero 2008	Mexico	CNIN
*C.niger*	Wittmer 1986	Costa Rica	NHMB
*C.pallidus*	Schaeffer 1904	USA	NHMUK
*C.pedregalensis*	Zaragoza-Caballero1975	Mexico	CNIN
*C.punctatissimus*	Wittmer 1976	Mexico	NMNH
*C.saasil* sp. nov.	This work	Honduras	CBG
*C.sonoraensis*	Zaragoza-Caballero 2008	Mexico	CNIN
*C.tsiik* sp. nov.	This work	Belize	NMNH
*C.tupae*	Vega-Badillo et al. 2021	Mexico	CNIN
*C.villae*	Zaragoza-Caballero 1984	Mexico	CNIN
*C.wittmeri*	Zaragoza-Caballero 1984	Mexico	CNIN
*C.xiinbali*	Vega-Badillo et al. 2021	Guatemala	CNIN
*C.zuritai* sp. nov.	This work	Costa Rica	NMNH

#### Female and immature stages.

Unknown.

#### Remarks.

*Cenophengus* is morphologically similar to *Cleicosta*: both genera exhibit separated tentorial pits, vertical frons and simple tarsomeres. Additionally, in *Cenophengus*, the pronotum is rectangular and each elytron leaving the last 3 abdominal segments exposed, in *Cleicosta* pronotum, it is subquadrate in shape (slightly wider than long) and each elytron is short, leaving the last 5 abdominal segments exposed. Other important characteristics in *Cenophengus* are of the parameres of the aedeagus: symmetrical in dorsal view, slightly widened towards the middle, apex with spines mesad; in *Cleicosta*, parameres narrowing slightly after middle towards apex, apex without spines.

##### Key to the species of *Cenophengus*

**Table:** 

1	Pronotum as long as wide; integument smooth	**2**
–	Pronotum longer than wide; integument chagreend or smooth	**5**
2	Interocular distance 1.5 times longer than eye width in dorsal view; posterior angles of pronutum rounded (Fig. 10D), almost inconspicous; branches of anterior cubital veins (CuA) of the hind wing present (Fig. 10E)	***C.hnogamui* Vega-Badillo et al. 2021 (Fig. 10)**
–	Interocular distance twice or longer than eye width; posterior angles of pronutum acute (Fig. 12D); branches of anterior cubital veins (CuA) of hind wing absent (Fig. 12E)	**3**
3	Body length not exceeding 3 mm; eyes circular in lateral view; posterior radial vein (RP) absent in hind wing	***C.huatulcoensis* Zaragoza-Caballero, 2008 (Fig. 12)**
–	Body longer than 3 mm; eyes oval in lateral view (Fig. 21C); posterior radial vein (RP) present in hind wing	**4**
4	Interocular distance 2.0–2.3 times longer than eye width; terminal maxillary palpomere shorter than preceding three combined	***C.munizi* Zaragoza-Caballero, 2008 (Fig. 21)**
–	Interocular distance 2.5 times eye width; terminal maxillary palpomere as long as preceding three combined	***C.mumui* Vega-Badillo et al. 2021 (Fig. 20)**
5	Integument smooth	**6**
–	Integument chagreened	**9**
6	Body length not exceeding 5 mm	**7**
–	Body length longer than 10 mm	**8**
7	Body pale brown; pronotum monochrome; antennal rami as long as respective antennomere; branching of anterior cubital veins (CuA) absent in hind wing; aedeagus with three teeth at the inner apex of paramere	***C.baios* Zaragoza-Caballero, 2003 (Fig. 4)**
–	Body darker brown; pronotum bicoloured; antennal rami 1.5 times as long as respective antennomere; anterior cubital veins (CuA) branched into CuA 1, CuA 2, CuA 3+4; aedeagus with one spine at the inner apex of paramere	***C.debilis* LeConte, 1881 (Fig. 2)**
8	Each elytron 4.0 times as long as wide; r3 vein absent	***C.magnus* Zaragoza-Caballero, 1988 (Fig. 15)**
–	Each elytron 4.5 times as long as wide; r3 vein present	***C.major* Wittmer, 1976 (Fig. 16)**
9	Antennae short (less than twice the length of the pronotum)	**10**
–	Antennae long (more than twice the length of pronotum)	**20**
10	Body pale brown or yellow; eyes 3/4 as long as head in lateral view	**11**
–	Body brown or dark brown with pronotum yellow-orange; eyes 1/2 or 3/4 as long as head in lateral view	**14**
11	Each elytron 3.0 to 3.5 times as long as wide	**12**
–	Each elytron 4.0 times as long as wide	***C.sonoraensis* Zaragoza-Caballero, 2008 (Fig. 27)**
12	Body yellow; interocular distance 1.5 times eye width; terminal maxillary palpomere as long as the preceding three combined	**13**
–	Body pale brown; interocular distance 2.0 times eye width; terminal maxillary palpomere shorter than preceding three combined	***C.gorhami* Zaragoza-Callero, 1986 (Fig. 9)**
13	Pronotal disc without groove along mid-line	***C.pallidus* Schaeffer, 1904 (Fig. 23)**
–	Pronotal disc with groove along mid-line	***C.ciceroi* Wittmer, 1981 (Fig. 6)**
14	Body dark brown with pronotum yellow-orange; eyes 3/4 as long as head in lateral view	**15**
–	Body entirely dark brown; eyes 1/2 as long as head in lateral view	**16**
15	Interocular distance twice as long as eye width (Fig. 13C); antennomere 1 longer than next two combined	***C.kikapu* Vega-Badillo et al. 2021 (Fig. 13)**
–	Interocular distance 3.5 times eye width (Fig. 14C); antennomere 1, 1.7 times longer than next two combined	***C.longicollis* Wittmer, 1976 (Fig. 14)**
16	Interocular distance 2.5 times width of eye; 4^th^ (terminal) maxillary palpomere longer than preceding three combined	***C.cuicatlaensis* Zaragoza-Caballero, 2008 (Fig. 7)**
–	Interocular distance 3.0 or more than 3 times width of eye; 4^th^ maxillary palpomere shorter or equal to preceding three combined	**17**
17	Antennomere 1 is longer than antennomeres 2 and 3 combined; 4^th^ (terminal) maxillary palpomere shorter than preceding three combined	***C.tsiik* sp. nov. (Fig. 28)**
–	Antennomere 1 as long as antennomeres 2 and 3 combined; 4^th^ (terminal) maxillary palpomere equal to preceding three combined	**18**
18	Interocular distance 3.5 to 4.0 times eye width	**19**
–	Interocular distance 3.0 times eye width	***C.niger* Wittmer, 1986 (Fig. 22)**
19	Pronotal disc without longitudinal carina	***C.brunneus* Wittmer, 1976 (Fig. 5)**
–	Pronotal disc with a longitudinal carina in posterior portion of pronotum strongly visible, with a length that does not reach the median length of the pronotum	***C.villae* Zaragoza-Caballero, 1984 (Fig. 30)**
20	Body length not exceeding 6 mm	**21**
–	Body longer than 9 mm	**22**
21	Body brown, except antennae yellow-brown; interocular distance twice eye width; antennomere 1 longer than antennomeres 2 and 3 combined	***C.tupae* Vega-Badillo et al. 2021 (Fig. 29)**
–	Body brown, yellowish mandibles with darker tips; interocular distance 3.0 times eye width; antennomere 1 shorter than antennomeres 2 and 3 combined	***C.howdeni* Zaragoza-Caballero, 1986 (Fig. 11)**
22	Interocular distance at most twice eye width	**23**
–	Interocular distance more than twice eye width	**26**
23	Body orange, except antennae, abdomen, hind wings and legs dark brown; terminal maxillary palpomere half as long as preceding three combined	***C.gardunoi* sp. nov. (Fig. 8)**
–	Body yellow or brown, pronotum yellow orange or brown; terminal maxillary palpomere equal or 2/3 as long as preceding three combined	**24**
24	Pronotum with uniform colouration; posterior radial vein (RP) length twice less than the length of MP1+2 (Fig. 31E)	**25**
–	Pronotum mottled with darker brown spots; posterior radial vein (RP) length 1.5 times less than the length of MP1+2 (Fig. 17E)	***C.marmoratus* Wittmer, 1976 (Fig. 17)**
25	Body brown, with pronotum dark brown near mid-line; elytra each 3.5 times as long as wide; r3 vein present	***C.wittmeri* Zaragoza-Caballero, 1984 (Fig. 31)**
–	Body yellow; elytra each 4.6 times as long as wide; r3 vein absent	***C.saasil* sp. nov (Fig. 26)**
26	Body black; terminal maxillary palpomere as long as preceding three combined	***C.mboi* Vega-Badillo et al. 2021 (Fig. 18)**
–	Body dark brown or body dark brown and pronotum yellow-orange; terminal maxillary palpomere shorter to longer than preceding three combined	**27**
27	Body dark brown; pronotal disc with a longitudinal carina in posterior portion of pronotum strongly visible, with a length exceeding the median length of the pronotum; elytra 5.4 times as long as wide	***C.punctatissimus* Wittmer, 1976 (Fig. 25)**
–	Body dark brown and pronotum yellow-orange, pronotal disc without longitudinal carina; each elytron less than 5.0 times as long as wide	**28**
28	Antennomere 1 as long as antennomeres 2 and 3 combined; terminal maxillary palpomere longer than preceding three combined	***C.pedregalensis* Zaragoza-Caballero, 1975 (Fig. 24)**
–	Antennomere 1 is longer than antennomeres 2 and 3 combined; terminal maxillary palpomere shorter than or equal to preceding three combined	**29**
29	Terminal maxillary palpomere shorter than preceding three combined; antennal rami 1.5 times respective antennomere	***C.zuritai* sp. nov. (Fig. 33)**
–	Terminal maxillary palpomere as long as preceding three combined; antennal rami twice as long as respective antennomere	***C.xiinbali* Vega-Badillo et al. 2021 (Fig. 32)**

### Cenophengus debilis

Taxon classificationAnimaliaColeopteraPhengodidae

LeConte, 1881

F6592DD2-F0D8-52ED-B9BB-A0833639341B

Fig. 2A–H

Cenophengus debilis LeConte, 1881: 41.

#### Type locality.

California, USA (Fig. 3).

#### Type material examined.

***Holotype*** ♂: “Type /2813” “*Cenophengus/ debilis* Lec.” “Cal.” | MCZ, url: http:/insects.oeb.harvard.edu.

#### Remarks.

*Cenophengusdebilis* is morphologically similar to *C.baios*, but can be distinguished by the antennal rami length, branching of the hind wing and interantennal distance. In *C.debilis*, the branching of the anterior cubital veins (CuA) is present on the hind wing, whereas in *C.baios*, it is unbranched. The interantennal distance is wider than length of first antennomere in *C.debilis*, in *C.baios*, it is narrower than length of antennomere 1. Additionally, in *C.debilis*, the antennal rami are 1.5 times as long as the respective antennomere, whereas in *C.baios*, they are as long as the respective antennomere.

#### Diagnosis.

Integument smooth and pronotum bicoloured (yellow-orange and dark brown); antennal rami are 1.5 times as long as the respective antennomere; clypeus totally sclerotised; anterior cubital veins (CuA) on hind wing branched; aedeagus with one spine at the inner apex of paramere.

#### Redescription.

**Figure 2.**
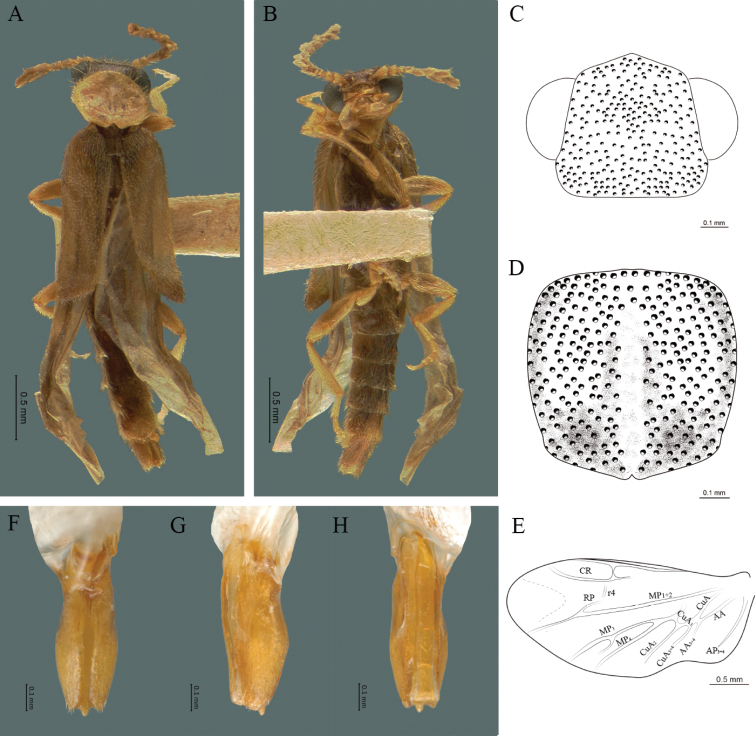
*Cenophengusdebilis* LeConte, 1881, male. Habitus: **A** dorsal **B** ventral **C** head dorsal **D** pronotum dorsal **E** hind wing. Wing venation: CR = Radial Cell; r3 = radial 3 vein; r4 = radial 4 vein; RP = Posterior Radial vein; MP1+2 = Posterior Median vein; CuA = Cubital vein; AA and AP = Anterior and Posterior Anal veins. Aedeagus: **F** dorsal view **G** lateral view **H** ventral view.

**Male.** Body length 4.0–5.3 mm; maximum body width 0.64–0.70 mm (pronotum). Body brown, except for the head, posterior part of the pronotum, scutellum and elytra are dark brown (Fig. 2A, B). ***Head*.** Wider (0.64–0.82 mm) (0.68 ± 0.025 mm, n = 6) than long (0.35–0.44 mm) (0.37 ± 0.036 mm, n = 6) (Fig. 2C), at eye level, almost as wide as the pronotum, integument smooth, punctures smaller than eye facets and separated by approximately 2.5 punctured diameters, each puncture bearing a yellow-orange seta; interantennal distance (0.10–0.13 mm) (0.11 ± 0.012 mm, n = 6) less than the length of antennomere 1 (0.12–0.17 mm) (0.15 ± 0.016 mm, n = 6); eyes 3/4 as long as head in lateral view, longer (0.25–0.35 mm) (0.30 ± 0.031 mm, n = 6) than wide (0.11–0.19 mm) (0.16 ± 0.031 mm, n = 6); interocular distance (0.40–0.46 mm) (0.42 ± 0.031 mm, n = 6) 2.5 times eye width; short antennae (1.16–1.40 mm) (1.2 ± 0.099 mm, n = 6), less than twice the length of the pronotum; antennomere 1 (0.10–0.15 mm) (0.11 ± 0.023 mm, n = 6) longer than the next two combined, antennomere 3 cup-shaped, the 4 (0.08–0.11 mm) (0.98 ± 0.011 mm, n = 6) shorter than the following antennomeres; 5 to 11 about equal in length (0.10–0.12 mm) (0.11 ± 0.08 mm, n = 6), 12 (terminal) (0.16–0.20 mm) (0.17 ± 0.020 mm, n = 6), antennal rami lanceolate in lateral view, 1.5 times as long as respective antennomere; terminal maxillary palpomere robust, securiform (0.15–0.17 mm) (0.16 ± 0.009 mm, n = 6), is shorter than the preceding three combined; terminal labial palpomere spindle-shaped (0.10–0.12) (0.11 ± 0.008 mm, n = 6), 5 times as long as preceding one (0.02–0.03 mm) (0.25 ± 0.005 mm, n = 6). ***Thorax*.** Pronotum longer (0.63–0.72 mm) (0.68 ± 0.038 mm, n = 6) than wide (0.64–0.70 mm) (0.68 ± 0.025 mm, n = 6); (Fig. 2D); integument smooth, punctures smaller than eye facets and separated by approximately 1.5 punctured diameters, each puncture bearing a yellow-orange seta, convex disc, posterior margin curved with middle notch, sides convex, anterior and posterior angles rounded; mesosternal suture complete; scutellum with posterior margin rounded ; each elytron 3.5 times as long (1.68–1.75 mm) (1.71 ± 0.027 mm, n = 6) as wide (0.44–0.48 mm) (0.45 ± 0.013 mm, n = 6), convex, without longitudinal costae, elytral apex right angled; hind wings with posterior radial vein (RP) length 4 times less than the length of MP1+2, radial cell closed, r3 vein present, r4 vein reduced (not reaching the RP or the radial cell), those of the anterior anal and posterior anal sectors, evident (Fig. 2E). Legs: tarsomere 1 of pro-, meso- and metathoracic legs longer than 2. ***Abdomen*.** Integument shiny, punctured, with long dense setae, sternite 7 with margin sinuate, sternite 8 with margin notched; aedeagus with one spine at the inner apex of paramere (Fig. 2F–H).

#### Immatures and females.

Unknown.

#### Distribution.

**Figure 3.**
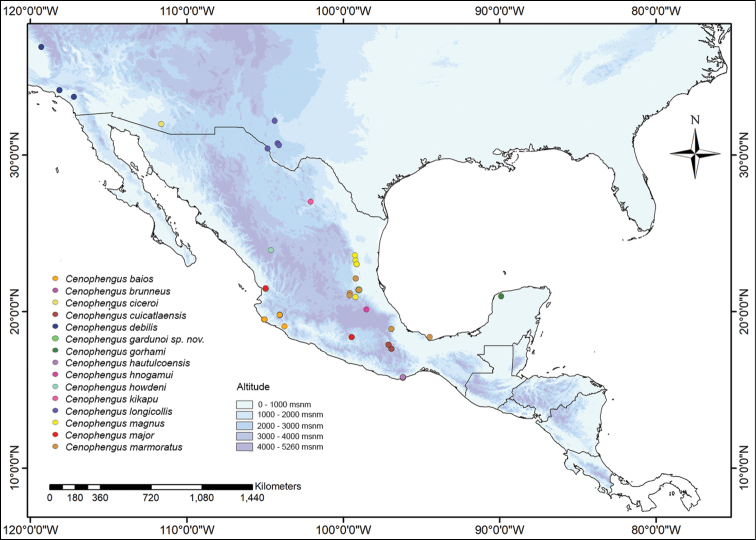
Map of southern North America showing specimen localities for *Cenophengus* spp.

USA: California (Fig. 3).

#### Additional material examined.

“USA CA: 10 mi. NE of /Trimmer/ 24.VI. 93. / Lot 2 BF&JL/ Carr.” “J. & B. Carr Coll. / Bequest to CNCI/ August, 2000” (3) |CNCI; “Sun City, Calif. / Riverside Co./ VIII. 28.1968/ D. E. Bright” “*Cenophengus /debilis* / det. W. Wittmer” (1) |CNCI; “3 mi. N Refugio/ Beach, Calif. / Sta. Barb. Co. / July 4, 1965” “J.S. Bucket/ Collector” (1) |CNIN; “Pasadena/ 13 – 15/ VI.1917 Cal. / A. Fenyes” “Electric light” “*Cenophengus/ debilis* Lec. / det. D. Linsdale 1960” (1) |CNIN.

### Cenophengus baios

Taxon classificationAnimaliaColeopteraPhengodidae

Zaragoza-Caballero, 2003

E164D703-C2F1-5DFE-B34E-E7826C8427CC

Fig. 4A–H

Cenophengus baios Zaragoza-Caballero, 2003: 159.

#### Type locality.

Jalisco, Mexico.

#### Type material examined.

***Holotype*** ♂: “MEXICO: Jalisco/ Est. Biol. Chamela 7/ Cuenca 1 TM. / 3-8- VIII-1992/ Trampa Malaise 237/ Col. A. Rodríguez” |CNIN. Paratype ♂: “MEXICO: Jalisco/ San Buenaventura/ 3-8-VI-1992 Alt. 720 m/ 19°47.6' N 104°03.32' O/ Trampa Malaise 4” “Cols. V.H. Toledo/ M.E. Guardado, A. Soria/ S. Zaragoza, L.F. Novelo/ E. Ramírez, M.A. Sarmiento” |CNIN; ***Paratype*** ♂: ” MEXICO: Jalisco/ Estación Biológica Chamela/ 13-XI-1987 en hojarasca/ R. Terron” |CNIN.

#### Remarks.

*Cenophengusbaios* is morphologically similar to *C.huatulcoensis*, but can be distinguished by the antennal rami length, interantennal and interocular distances. In *C.baios*, the interantennal distance is shorter than the length of the antennomere 1, whereas in *C.huatulcoensis*, it is equal. The interocular distance is 3.5 times eye width in *C.baios* and in *C.huatulcoensis*, it is 3 times longer. Additionally, in *C.baios*, the antennal rami are as long as the respective antennomere, whereas in *C.huatulcoensis*, they are twice as long as the respective antennomere.

#### Diagnosis.

Integument smooth, antennae less than twice the length of the pronotum, antennal rami as long as the respective antennomere, pronotum as long as wide, each elytron 2.7 times as long as wide and branching of the anterior cubital veins (CuA) absent in the hind wing; aedeagus with three teeth at the inner apex of paramere.

#### Redescription.

**Figure 4.**
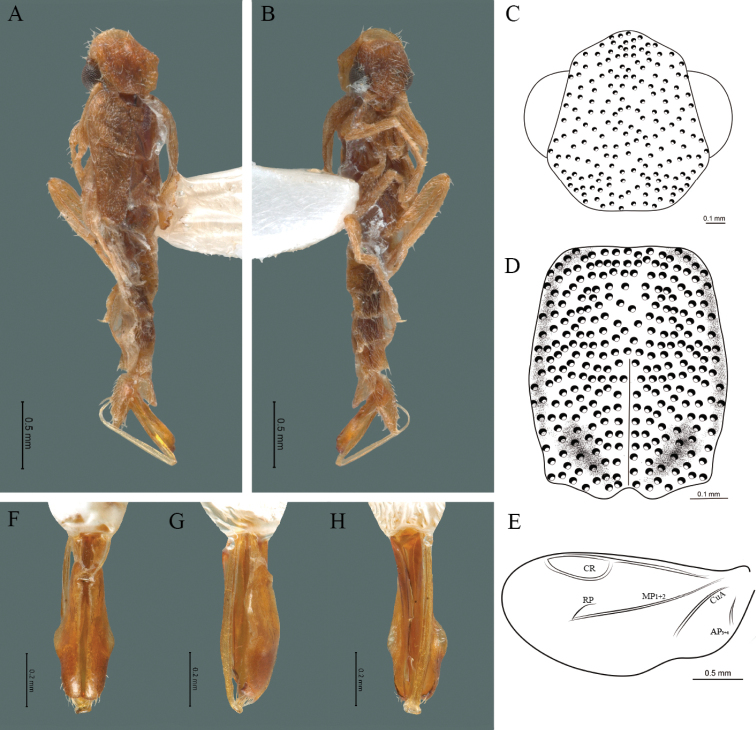
*Cenophengusbaios* Zaragoza-Caballero, 2003, male. Habitus: **A** dorsal **B** ventral **C** head dorsal **D** pronotum dorsal **E** hind wing. Wing venation: CR = Radial Cell; RP = Posterior Radial vein; MP1+2 = Posterior Median vein; CuA = Cubital vein; AP = Posterior Anal vein. Aedeagus: **F** dorsal view **G** lateral view **H** ventral view.

**Male.** Body length 3.8–4.0 mm: maximum body width 0.50–0.52 mm (pronotum). Body pale brown, except for head which is dark brown (Fig. 4A, B). ***Head*.** Wider (0.49–0.56 mm) (0.52 ± 0.022 mm, n = 10) than long (0.33–0.40 mm) (0.35 ± 0.019 mm, n = 10) (Fig. 4C), at eye level, as wide as the pronotum, integument smooth, punctures as large as eye facets and separated by approximately 2 punctured diameters, each puncture bearing a yellow-orange seta; interantennal distance (0.06–0.10 mm) (0.67 ± 0.013 mm, n = 10) less than the length of antennomere 1 (0.10–0.13 mm) (0.11 ± 0.013 mm, n = 10); eyes 1/2 as long as head in lateral view, longer (0.16–0.23 mm) (0.19 ± 0.024 mm, n = 10) than wide (0.08–0.11 mm) (0.95 ± 0.014 mm, n = 10); interocular distance (0.30–0.35 mm) (0.33 ± 0.016 mm, n = 10) 3.5 times eye width, slightly excavated; short antennae (1.09–1.36 mm) (1.15 ± 0.085 mm, n = 10) less than twice the length of the pronotum; antennomere 1 (0.10–0.13 m) (0.11 ± 0.013 mm, n = 10) as long as the next two combined, 3 cup-shaped, from 4 to 11 about equal in length (0.1–0.12) (1.05 ± 0.0084, n = 10), 12 (terminal) (0.10–0.15 mm) (0.12 ± 0.017 mm, n = 10), antennal rami lanceolate in lateral view, as long as the respective antennomere; terminal maxillary palpomere securiform (0.13–0.15 mm) (0.14 ± 0.006 mm, n = 10), as long as the preceding three combined; terminal labial palpomere spindle-shaped (0.05–0.07 mm) (0.06 ± 0.004 mm, n = 10), 3 times as long as preceding one (0.02–0.03 mm) (0.21 ± 0.003 mm, n = 10). ***Thorax*.** Pronotum as long (0.55–0.60 mm) (0.58 ± 0.020 mm, n = 10) as wide (0.50–0.55 mm) (0.52 ± 0.020 mm, n = 10) (Fig. 4D); integument smooth, punctures smaller than eye facets and separated by approximately 1 punctured diameter, each puncture bearing a yellow-orange seta, disc convex, with a longitudinal carina in posterior portion of pronotum strongly visible, with a length exceeding the median length of the pronotum, weakly elevated dorsally forming a small depression in the basal part of each side and a posterior margin curved with middle notch, sides almost straight, anterior and posterior angles rounded; mesosternal suture complete; scutellum with posterior margin rounded; each elytron 3.1 times as long (0.82–1.0 mm) (0.90 ± 0.071 mm, n = 10) as wide (0.26–0.34 mm) (0.29 ± 0.028 mm, n = 10), convex, without longitudinal costae, elytral apex rounded; hind wings with posterior radial vein (RP) length 4.7 times less than the length of MP1+2, radial cell closed, r3 and r4 veins absent, those of the anterior anal and posterior anal sectors, absent (Fig. 4E). Legs: tarsomere 1 of pro-, meso- and metathoracic legs is longer than 2. ***Abdomen*.** Integument shiny, punctured, with long dense setae, sternite 7 with margin sinuate, sternite 8 with margin notched; aedeagus with three teeth at the inner apex of paramere (Fig. 4F–H).

#### Female and immatures.

Unknown.

#### Distribution.

Mexico: Jalisco and Colima (Fig. 3).

#### Additional material examined.

“MEXICO. Jalisco, San Buenaventura/ 19° 47'.614" N 104° /03'.324" O. Alt. 720 m/ TL3 09-02-1997/ Cols. F. A. Noguera, S. / Zaragoza, E. Ramírez y /E. González” (1) |CNIN; “MÉXICO: Jalisco, San Buenaventura/ 19° 46'61" N 104°/ 03'32" O Alt 620 m/ TL2 09-II-97” “Cols. F. A. Noguera, / S. Zaragoza, E. Ramírez, / E. González” “*Cenophengusbaios*/ det. S. Zaragoza” (5) |CNIN; “MÉXICO: Jalisco, San Buenaventura/ 19° 46'61"N 104°/ 03'32" O Alt 620 m/ TL2 09-II-97/ Col S. Zaragoza” “*Cenophengusbaios* /det. S. Zaragoza” (1) |CNIN; “MÉXICO: Colima, 0.5 km /S Jiliotupa Alt. 330 m Tl 4/ 19° 03' 05.6" N/ 103° 45' 28.8"O/28-IV-2006” “Cols. S. Zaragoza, F. A. Noguera/ E. Ramírez, E. González/ L. Salas” (1) |CNIN.

### Cenophengus brunneus

Taxon classificationAnimaliaColeopteraPhengodidae

Wittmer, 1976

3A6281AD-0329-57BD-88E2-095A125D75E7

Fig. 5A–H

Cenophengus brunneus Wittmer, 1976: 453.

#### Type locality.

Veracruz, Mexico.

#### Type material examined.

***Holotype*** ♂: “MEXICO: Veracruz/ Córdoba / Dr. A. Fenyes” “*Cenophengus/ brunneus* det. W. Wittmer” “HOLOTYPUS” “Type No. / 73887/ USNM” | NMNH.

#### Remarks.

*Cenophengusbrunneus* is sister to *C.villae* (Vega-Badillo et al. 2021b), but can be distinguished by the interocular distance: in *C.brunneus* it is 3.5 times eye width, whereas in *C.villae*, it is 4.0 times longer. Additionally, in *C.brunneus*, the pronotal disc is convex, without a longitudinal carina, whereas in *C.villae*, it has a longitudinal carina.

#### Diagnosis.

Body brown, integument chagreened, antennae less than twice the length of the pronotum, antennal rami 1.5 times the respective antennomere and each elytron 5.1 times as long as wide; aedeagus with three teeth at the inner apex of paramere.

#### Redescription.

**Figure 5.**
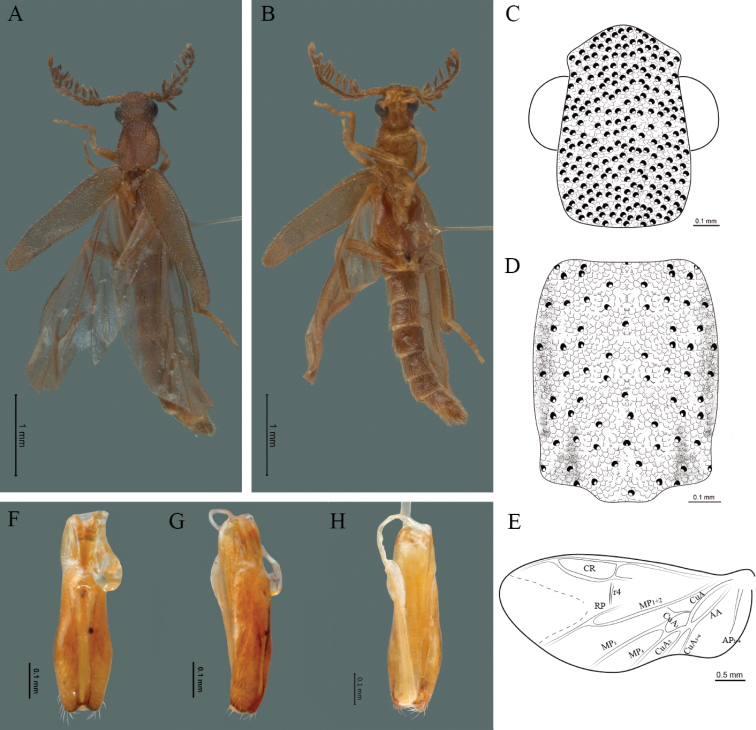
*Cenophengusbrunneus* Wittmer, 1976, male. Habitus: **A** dorsal **B** ventral **C** head dorsal **D** pronotum dorsal **E** hind wing. Wing venation: CR = Radial Cell; r3 = radial 3 vein; r4 = radial 4 vein; RP = Posterior Radial vein; MP1+2 = Posterior Median vein; CuA = Cubital vein; AA and AP = Anterior and Posterior Anal veins. Aedeagus: **F** dorsal view **G** lateral view **H** ventral view.

**Male.** Body length 4.30 mm; maximum body width 0.47 mm (pronotum). Body brown, legs paler (Fig. 5A, B). ***Head*.** Wider (0.50) than long (0.40) (Fig. 5C), at eye level, slightly wider than the pronotum, integument chagreened, punctures as large as eye facets and separated by approximately 1 punctured diameter, each puncture bearing a yellow-brown seta; interantennal distance (0.04 mm) less than length of the antennomere 1 (0.12 mm); eyes 1/2 as long as head in lateral view, longer (0.21 mm) than wide (0.09 mm); interocular distance (0.32 mm) 3.5 times eye width; short antennae (1.59 mm) less than twice the length of the pronotum; antennomere 1 (0.12 mm) as long as the next two combined, 3 cup-shaped, 4 (0.13 mm) shorter than the following antennomeres, 5 to 11 about equal in length (0.14 mm), 12 (terminal) (0.20 mm), antennal rami lanceolate in lateral view, 1.5 times the respective antennomere; terminal maxillary palpomere uniform, securiform (0.17 mm), as long as the preceding three combined (0.18 mm); terminal labial palpomere spindle-shaped (0.07 mm), 3 times as long as preceding one (0.02 mm). ***Thorax*.** Pronotum longer (0.64 mm) than wide (0.47 mm) (Fig. 5D); integument chagreened, punctures as large as eye facets and separated by approximately 4 punctured diameters, each puncture bearing a yellow-brown seta; convex disc, weakly elevated dorsally forming a small depression in the basal part of each side, posterior margin curved with middle notch, sides almost straight, anterior angles rounded and posterior angles acute; mesosternal suture complete; scutellum with posterior margin rounded; each elytron 5.1 times as long (1.64 mm) as wide (0.32 mm), convex, without longitudinal costae, elytral apex rounded; hind wings with posterior radial vein (RP) length 3.8 times less than the length of MP1+2, radial cell closed, r3 vein present, r4 vein reduced (not reaching the RP or the radial cell), those of the anterior anal and posterior anal sectors, evident (Fig. 5E). Legs: tarsomeres 1 and 2 of the prothoracic legs with a similar length and tarsomere 1 of meso- and metathoracic legs is longer than 2. ***Abdomen*.** Integument shiny, punctured, with long dense setae, sternite 7 with margin sinuate, sternite 8 with margin notched; aedeagus with three teeth at the inner apex of paramere (Fig. 5F–H).

#### Female and immatures.

Unknown.

#### Distribution.

Mexico: Veracruz (Fig. 3).

### Cenophengus ciceroi

Taxon classificationAnimaliaColeopteraPhengodidae

Wittmer, 1981

90B56AFD-FBF1-54CE-8BC2-FF4C1562D073

Fig. 6A–H

Cenophengus ciceroi Wittmer, 1981: 106.

#### Type locality.

USA.

#### Type material examined.

***Holotype*** ♂: USA: “Az. Pima Co. Tucson Mts. / Saguaro Nat. Mon./ 5-APR-80/ Cicero” “Red Hills / visiter center” “Note Luminescent spots/ vaguely indicated as/ two white patches on/ last tergite” “*Cenophengus / ciceroi* det. W. Wittmer” “HOLOTYPUS” “Type No./ 100336 / USMN” | NMNH.

#### Remarks.

*Cenophengusciceroi* is sister to *C.gorhami* (Vega-Badillo et al. 2021b), but can be distinguished by the antennal rami length, interocular distance and the terminal maxillary palpomere. In *C.ciceroi*, the interocular distance is 1.5 times eye width, whereas in *C.gorhami*, it is twice longer. The terminal maxillary palpomere is as long as the preceding three combined in *C.ciceroi*; in *C.gorhami*, it is shorter than the preceding three combined. Additionally, in *C.ciceroi*, the antennal rami are twice as long as the respective antennomere, whereas in *C.gorhami*, they are 1.5 times as long as the respective antennomere.

#### Diagnosis.

Integument chagreened, antennae less than twice the length of the pronotum, antennal rami twice as long as the respective antennomere, pronotum longer than wide and each elytron 3.3 times as long as wide; aedeagus with three teeth at the inner apex of paramere.

#### Redescription.

**Figure 6.**
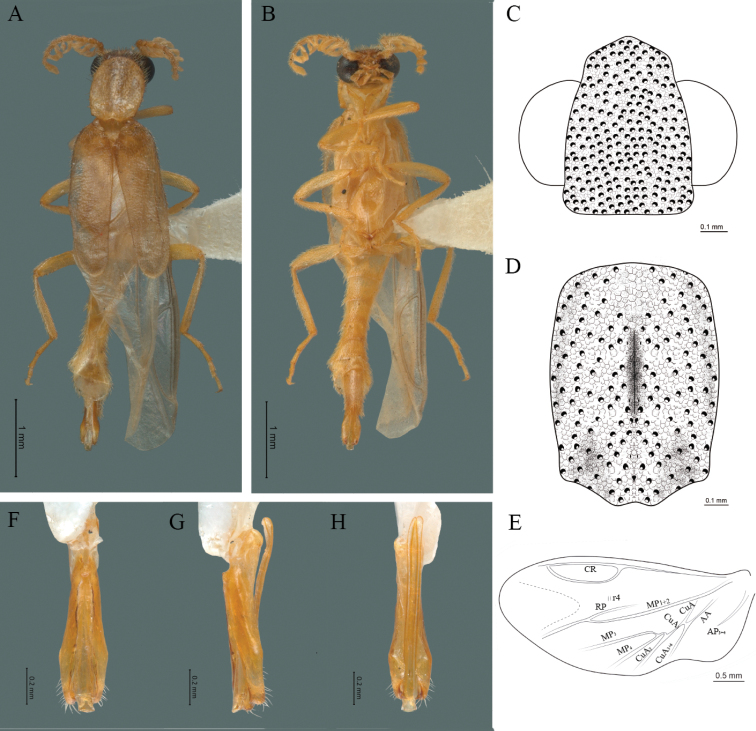
*Cenophengusciceroi* Wittmer, 1981, male. Habitus: **A** dorsal **B** ventral **C** head dorsal **D** pronotum dorsal **E** hind wing. Wing venation: CR = Radial Cell; r4 = radial 4 vein; RP = Posterior Radia vein l; MP1+2 = Posterior Median vein; CuA = Cubital vein; AP = Posterior Anal vein. Aedeagus: **F** dorsal view **G** lateral view **H** ventral view.

**Male.** Body length 4.20 mm; maximum body width 0.60 mm (pronotum). Head dark brown to black, rest of the body, antennae and legs included, yellow to pale brown (Fig. 6A, B). ***Head*.** Wider (0.75 mm) than long (0.42 mm), at eye level, wider than the pronotum (Fig. 6C), integument chagreened, punctures as large as eye facets and separated by approximately 1 punctured diameter, each puncture bearing a yellow-orange seta; interantennal distance (0.04 mm) less than the length of antennomere 1 (0.16 mm); eyes 3/4 as long as head in lateral view, longer (0.37 mm) than wide (0.17 mm); interocular distance (0.25 mm) 1.5 times eye width; antennae short (1.51 mm) less than twice the length of the pronotum; antennomere 1 (0.16 mm) longer than the next two combined, antennomere 3 cup-shaped, 4 (0.10 mm) shorter than following antennomeres, 5 to 11 about equal in length (0.15 mm), 12 (terminal) (0.20 mm), antennal rami lanceolate in lateral view, twice as long as the respective antennomere; terminal maxillary palpomere robust, securiform (0.23 mm), as long as the preceding three combined (0.24 mm); terminal labial palpomere spindle-shaped (0.1), 3 times as long as preceding one (0.03 mm). ***Thorax*.** Pronotum longer (0.89 mm) than wide (0.64 mm) (Fig. 6D); integument chagreened with punctures as large as facets and separated by approximately 2 punctured diameters, each puncture bearing a yellow-orange seta; disc convex, with groove along mid-line, posterior margin curved with middle notch, sides almost straight, anterior angles rounded and posterior angles acute; mesosternal suture incomplete; scutellum with posterior margin rounded; each elytron 3.3 times as long (1.88 mm) as wide (0.56 mm), convex, without longitudinal costae, elytral apex rounded; hind wings with posterior radial vein (RP) length 5 times less than the length of MP1+2, radial cell closed, r3 vein absent, r4 vein reduced (not reaching the RP or the radial cell), those of the anterior anal and posterior anal sectors, absent (Fig. 6E). Legs: tarsomere 1 of pro-, meso- and meathoracic legs is longer than 2. ***Abdomen*.** Integument shiny, punctured, with long dense setae, sternite 7 with margin sinuate, sternite 8 with margin notched; aedeagus with three teeth at the inner apex of paramere (Fig. 6F–H).

#### Female and immatures.

Unknown.

#### Distribution.

USA (Fig. 3).

### Cenophengus cuicatlaensis

Taxon classificationAnimaliaColeopteraPhengodidae

Zaragoza-Caballero, 2008

66A50348-37EB-567A-A995-708BC5FEDE8D

Fig. 7A–H

Cenophengus cuicatlaensis Zaragoza-Caballero, 2008: 153.

#### Type locality.

Oaxaca, Mexico.

#### Type material examined.

***Holotype*** ♂: “MEXICO: Oaxaca/ 23.5 km SSE Cuicatlán/ 17°37.582' N, 96°55.121' O/ 25-V-1998. Alt. 940 m/ trampa de Luz 2/ Cols. S. Zaragoza, A. Soria/ V. H. Toledo, E. Ramírez/ M.A. Morales” “*Cenophenguscuicatlaensis*/ S. Zaragoza-Caballero” | CNIN. ***Paratype*** ♂: “MÉXICO: Oaxaca / Dominguillo / 17°38'907"” N, 96°54' / 703" O, Alt. 760 m. / TL3 P475 m./ 26/01/1998 / Col. S. Zaragoza” (4) | CNIN.

#### Remarks.

*Cenophenguscuicatlaensis* is morphologically similar to *C.tsiik*, but can be distinguished by the interocular distance and the terminal maxillary palpomere. In *C.cuicatlaensis*, interocular distance is twice as long as eye width, whereas in *C.tsiik*, it is 3 times as long as eye width. Terminal maxillary palpomere is longer than the preceding three combined in *C.cuicatlaensis*, in *C.tsiik*, it is shorter than the preceding three combined.

#### Diagnosis.

Integument chagreened, antennae long more than twice the length of pronotum, antennal rami twice as long as the respective antennomere, pronotum longer than wide and each elytron 3.8 times as long as wide; aedeagus with three teeth at the inner apex of paramere.

#### Redescription.

**Figure 7.**
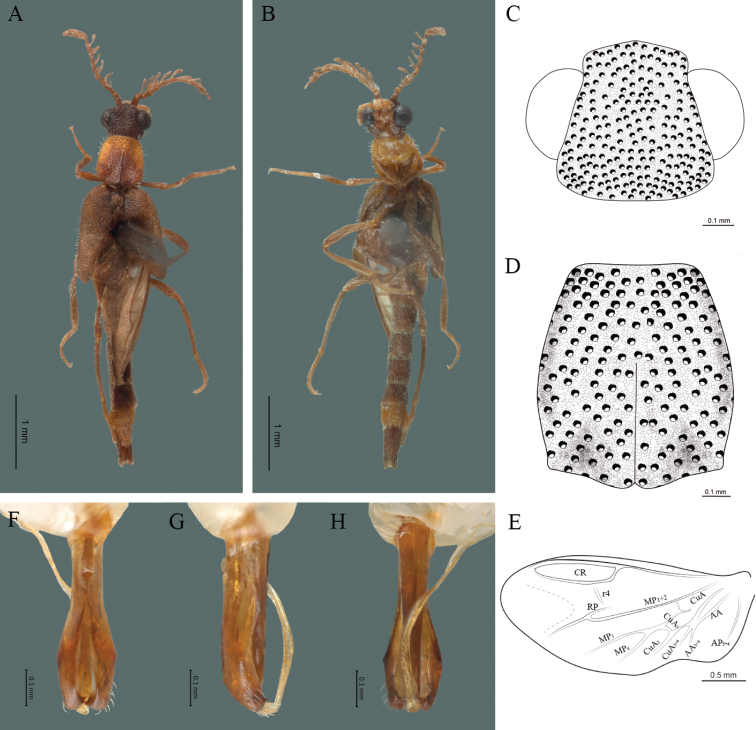
*Cenophenguscuicatlaensis* Zaragoza-Caballero, 2008, male. Habitus: **A** dorsal **B** ventral **C** head dorsal **D** pronotum dorsal **E** hind wing. Wing venation: CR = Radial Cell; r4 = radial 4 vein; RP = Posterior Radial vein; MP1+2 = Posterior Median vein; CuA = Cubital vein; AA and AP = Anterior and Posterior Anal veins. Aedeagus: **F** dorsal view **G** lateral view **H** ventral view.

**Male.** Body length 3.3–5.0 mm; maximum body width 0.55–0.75 mm (pronotum). Body brown, except for head dark brown; buccal parts and the two last sternites are yellowish coloured (Fig. 7A, B). ***Head*.** Wider (0.55–0.74 mm) (0.64 ± 0.074 mm, n = 5) than long (0.40–0.50 mm) (0.44 ± 0.037 mm, n = 5) (Fig. 7C), at eye level, wider than the pronotum, integument chagreened, punctures twice as large as eye facets and separated by approximately 0.5 punctured diameters, each puncture bearing a yellow-brown seta; interantennal distance (0.06–0.10 mm) (0.07 ± 0.016 mm, n = 5) less than the length of antennomere 1 (0.08–0.12 mm) (0.09 ± 0.017 mm, n = 5); eyes 1/2 as long as head in lateral view, longer (0.22–0.27 mm) (0.24 ± 0.022 mm, n = 5) than wide (0.11–0.17 mm) (0.14 ± 0.022 mm, n = 5); interocular distance (0.33–0.4) (0.36 ± 0.033, n = 5) 2.5 times longer than eye width; antennae long (1.13–1.57 mm) (1.33 ± 0.171 mm, n = 5) more than twice the length of pronotum; antennomere 1 (0.08–0.12 mm) (0.09 ± 0.017 mm, n = 5) longer than next two combined, antennomere 3 cup-shaped, 4 to 11 about equal in length (0.1–0.15 mm) (0.13 ± 0.020 mm, n = 5), 12 (terminal) (0.15–0.17 mm) (0.16 ± 0.008 mm, n = 5), antennal rami lanceolate in lateral view, twice as long as the respective antennomere; terminal maxillary palpomere robust, securiform (0.23–0.27 mm) (0.25 ± 0.017 mm, n = 5), longer than the preceding three combined; terminal labial palpomere spindle-shaped (0.07–0.08 mm) (0.74 ± 0.005 mm, n = 5), twice as long as preceding one (0.03–0.04 mm) (0.34 ± 0.005 mm, n = 5). ***Thorax*.** Pronotum longer (0.55–0.75 mm) (0.67 ± 0.075 mm, n = 5) than wide (0.5–0.63 mm) (0.58 ± 0.053 mm, n = 5) (Fig. 7D); integument chagreened, punctures twice as large as eye facets and separated by approximately 1 punctured diameter, each puncture bearing a yellow-brown seta, disc convex, with a longitudinal carina in posterior portion of pronotum strongly visible, with a length exceeding the median length of the pronotum , posterior margin curved with middle notch, sides almost straight, anterior angles rounded and posterior angles acute; mesosternal suture complete; scutellum with posterior margin rounded; each elytron 3.8 times as long (1.12–1.35 mm) (1.23 ± 0.102 mm, n = 5) as wide (0.26–0.36) (0.32 ± 0.039, n = 5), convex, without longitudinal costae, elytral apex rounded; hind wings with posterior radial vein (RP) length 5.6 times less than the length of MP1+2, radial cell closed, r3 vein absent, r4 vein developed (reaching the radial cell), those of the anterior anal and posterior anal sectors, evident (Fig. 7E). Legs: tarsomeres 1 and 2 of the prothoracic legs with a similar length and tarsomere 1 of meso- and metathoracic legs is longer than 2. ***Abdomen*.** Integument shiny, punctured, with long dense setae, sternite 7 with margin sinuate, sternite 8 with margin notched; aedeagus with three teeth at the inner apex of paramere (Fig. 7F–G).

#### Female and immatures.

Unknown.

#### Distribution.

Mexico: Oaxaca (Fig. 3).

### Cenophengus gardunoi

Taxon classificationAnimaliaColeopteraPhengodidae

Vega-Badillo, Morrone & Zaragoza-Caballero sp. nov.

6BAD96EA-6AD2-5054-BADB-FAFD3D0B5182

http://zoobank.org/B66C49DC-CDF6-4ED8-8050-F2C5B0B11619

Fig. 8A–H

#### Type locality.

San Luis Potosí, Mexico.

#### Type material.

***Holotype*** ♂: “MEXICO: S.L.P., Mun. / Xilitla 15 mi. SW. / Xilitla, 1500 m., 20-III-1988/ R. E. Jones P. W. / Kovarik, Colls” “From the Michael / Ivie Collection” (TIP-COL) |CNIN.

#### Remarks.

*Cenophengusgardunoi* is morphologically similar to *C.major*, but can be distinguished by the integument and r3 vein. In *C.gardunoi*, the integument is chagreened, whereas in *C.major*, it is smooth; the r3 vein is absent in *C.gardunoi*, whereas in *C.major*, it is present.

#### Diagnosis.

This species can be distinguished by the chagreened integument, antennae long, more than twice the length of pronotum, antennal rami 3 times the respective antennomere and each elytron 4.4 times as long as wide, with two longitudinal costae and aedeagus with three teeth at the inner apex of paramere.

#### Description.

**Figure 8.**
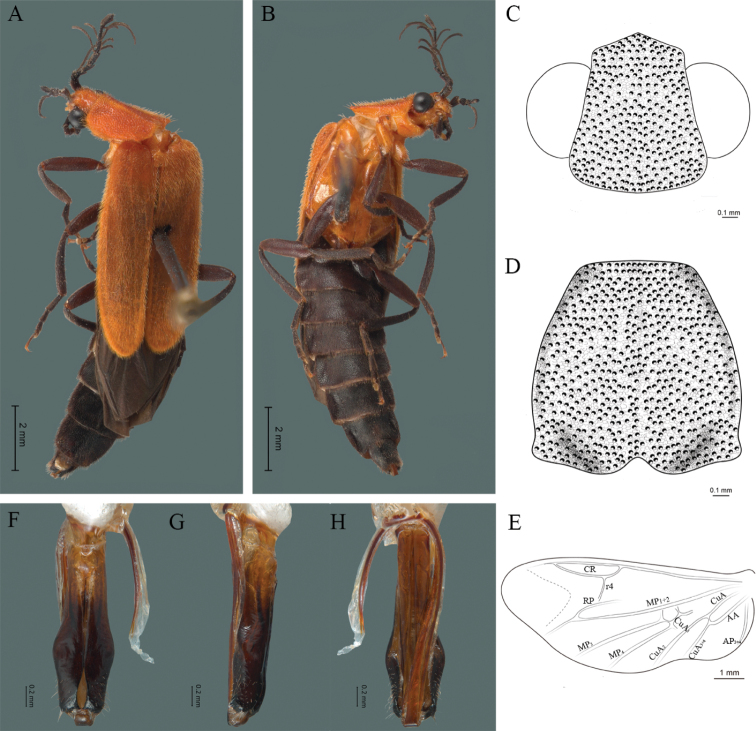
*Cenophengusgardunoi* Vega-Badillo, Morrone & Zaragoza-Caballero, sp. nov., male. Habitus: **A** dorsal **B** ventral **C** head dorsal **D** pronotum dorsal **E** hind wing. Wing venation: CR = Radial Cell; r4 = radial 4 vein; RP = Posterior Radial vein; MP1+2 = Posterior Median vein; CuA = Cubital vein; AA and AP = Anterior and Posterior Anal veins. Aedeagus: **F** dorsal view **G** lateral view **H** ventral view.

**Male.** Body length 16.0 mm; maximum body width 2.0 mm (pronotum). Body orange, except for the antennae, maxillary palpi, labial palpi, abdomen, hind wings and legs dark brown (Fig. 8A, B). ***Head*.** Wider (1.5 mm) than long (0.72 mm) (Fig. 8C), at eye level, less wide than the pronotum, integument chagreened, punctures twice as large as eye facets and separated by approximately 0.5 punctured diameters, each puncture bearing a yellow-orange seta; interantennal distance (0.15 mm) less than the length of antennomere 1 (0.45 mm); eyes 3/4 as long as head in lateral view, longer (0.60 mm) than wide (0.45 mm); interocular distance (0.80 mm) 1.7 times eye width; antennae long (4.50 mm) more than twice the length of pronotum; antennomere 1 (0.45 mm) longer than next two combined (0.30 mm), antennomere 3 cup-shaped, 4 (0.30 mm) shorter than the following antennomeres, 5 to 11 about equal in length (0.45 mm), 12 (terminal) (0.50 mm), antennal rami lanceolate in lateral view, 3 times the respective antennomere; terminal maxillary palpomere robust, securiform (0.40 mm), shorter than the preceding three combined (0.82 mm); terminal labial palpomere spindle-shaped (0.20 mm), twice as long as preceding one (0.10 mm). ***Thorax*.** Pronotum longer (2.5 mm) than wide (2.0 mm) (Fig. 8D); integument chagreened, punctures twice as large as eye facets and separated by approximately 1 punctured diameter, each puncture bearing a yellow-orange seta, disc convex, weakly elevated dorsally forming a small depression in the basal part of each side, posterior margin curved with middle notch, sides almost straight, anterior and posterior angles rounded; mesosternal suture complete; scutellum with small notch on posterior margin; each elytron 4.4 times as long (7.5 mm) as wide (1.7 mm), convex, with two longitudinal costae, elytral apex rounded; hind wings with posterior radial vein (RP) length twice less than the length of MP1+2, radial cell closed, r3 vein present, r4 vein developed (reaching the radial cell), those of the anterior anal and posterior anal sectors, evident (Fig. 8E). Legs: tarsomere 1 of pro-, meso- and metathoracic legs is longer than 2. ***Abdomen*.** Integument shiny, punctured, with long dense setae, sternite 7 with margin concave, sternite 8 with margin rounded; aedeagus with three teeth at the inner apex of paramere (Figs 1, 8F–H).

#### Female and immatures.

Unknown.

#### Distribution.

Mexico: San Luis Potosí (Fig. 3).

#### Etymology.

Species dedicated by the first author to Edgar Uriel Garduño Montes de Oca, her beloved life partner.

### Cenophengus gorhami

Taxon classificationAnimaliaColeopteraPhengodidae

Zaragoza-Callero, 1986

34B1F06D-2A43-5657-91F2-31E8E104A78C

Fig. 9A–H

Cenophengus gorhami Zaragoza-Caballero, 1986: 934.

#### Type locality.

Yucatan, Mexico.

#### Type material examined.

***Holotype*** ♂: “MEXICO: Yucatán/ Mérida/ VII-39-30-1964/ Paul J. Spangler” “S. Zaragoza C. det. / *Cenophengusgorhami* / Zaragoza” “BLNO/ 004121”| NMNH.

#### Remarks.

*Cenophengusgorhami* is sister to *C.ciceroi* (Vega-Badillo et al. 2021b), but can be distinguished by the interocular distance and the terminal maxillary palpomere. In *C.gorhami*, the interocular distance is twice as long as eye width, whereas in *C.ciceroi*, it is 1.5 times longer. The terminal maxillary palpomere is shorter than the preceding three combined in *C.gorhami*, in *C.ciceroi*, it is as long as the preceding three combined. Additionally, in *C.gorhami*, the pronotal disc with central longitudinal elevation, whereas in *C.ciceroi*, it has a groove along mid-line.

#### Diagnosis.

Integument chagreened, antennae long, more than twice the length of pronotum, antennal rami twice as long as the respective antennomere and elytra barely reaching the middle of the metasternum, each elytron 3.1 times as long as wide; aedeagus with three teeth at the inner apex of paramere.

#### Redescription.

**Figure 9.**
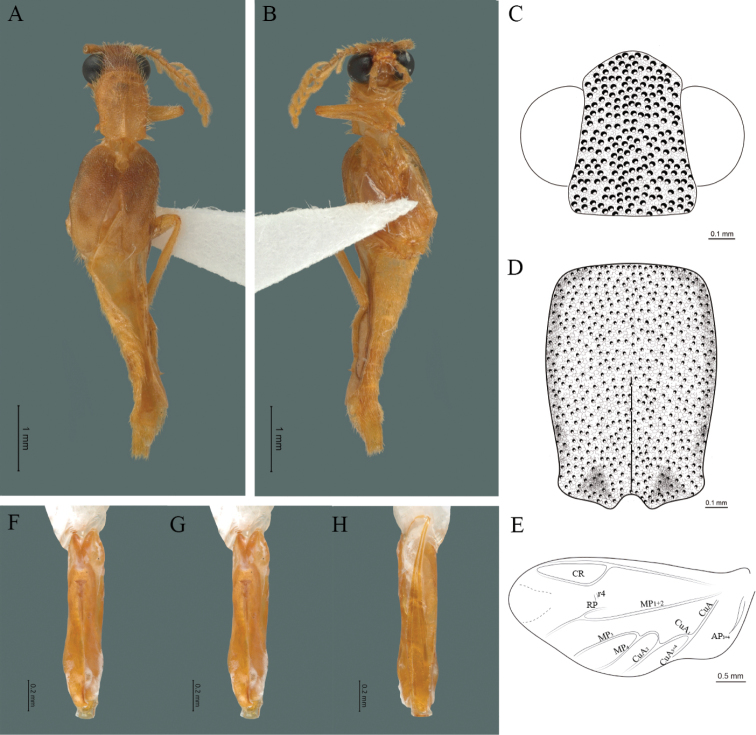
*Cenophengusgorhami* Zaragoza, 1986, male. Habitus: **A** dorsal **B** ventral **C** head dorsal **D** pronotum dorsal **E** hind wing. Wing venation: CR = Radial Cell; r4 = radial 4 vein; RP = Posterior Radial vein; MP1+2 = Posterior Median vein; CuA = Cubital vein; AP = Anterior and Posterior Anal veins. Aedeagus: **F** dorsal view **G** lateral view **H** ventral view.

**Male.** Body length 5.68–6.0 mm; maximum body width 0.71–0.75 mm (pronotum). Body yellow to pale brown, head a little darker, tip of mandibles almost black, elytra brown with yellowish apex (Fig. 9A, B). ***Head*.** Wider (0.91–0.96 mm) (0.935 ± 0.035 mm, n = 2) than long (0.55–0.58 mm) (0.565 ± 0.021 mm, n = 2) (Fig. 9C), at eye level, wider than the pronotum, integument chagreened, punctures twice as large as eye facets and separated by approximately 1 punctured diameter, each puncture bearing a yellow seta; interantennal distance (0.08–0.09 mm) (0.085 ± 0.007 mm, n = 2) less than the length of antennomere 1 (0.15–0.16 mm) (0.155 ± 0.007 mm, n = 2); eyes 3/4 as long as head in lateral view, longer (0.45–0.48 mm) (0.465 ± 0.021 mm, n = 2) than wide (0.25–0.27 mm) (0.26 ± 0.014 mm, n = 2); interocular distance (0.41–0.43 mm) (0.42 ± 0.014 mm, n = 2) twice as long as eye width; antennae long (1.75–1.88 mm) (1.81 ± 0.091 mm, n = 2) more than twice the length of pronotum; antennomere 1 (0.15–0.16 mm) (0.155 ± 0.007 mm, n = 2) longer than next two combined, antennomere 3 cup-shaped, 4 to 11 about equal in length (0.16–0.17 mm) (0.165 ± 0.007 mm, n = 2), 12 (terminal) (0.26–0.28 mm) (0.27 ± 0.014 mm, n = 2), antennal rami lanceolate in lateral view, twice as long as the respective antennomere; terminal maxillary palpomere robust, securiform (0.3–33 mm) (0.315 ± 0.021 mm, n = 2), shorter than the preceding three combined; terminal labial palpomere spindle-shaped (0.10–0.11 mm) (0.105 ± 0.007 mm, n = 2), 3 times as long as preceding one (0.03–0.04 mm) (0.035 ± 0.007 mm, n = 2). ***Thorax*.** Pronotum longer (1.03–1.08 mm) (1.05 ± 0.053 mm, n = 2) than wide (0.71–0.75 mm) (0.73 ± 0.028 mm, n = 2) (Fig. 9D); integument chagreened, punctures smaller than eye facets and separated by approximately 3 punctured diameters, each puncture bearing a yellow seta, disc convex, with a longitudinal carina in posterior portion of pronotum strongly visible, with a length equal to the median length of the pronotum, posterior margin curved with middle notch, sides almost straight, anterior angles rounded and posterior angles acute; mesosternal suture incomplete; scutellum with posterior margin rounded; elytra short, barely reaching the middle of the metasternum, each elytron 3.1 times as long (1.52–1.61 mm) (1.56 ± 0.063 mm, n = 2) as wide (0.48–0.5) (0.49 ± 0.014, n = 2), convex, without longitudinal costae, elytral apex rounded; hind wings with posterior radial vein (RP) length 5.3 times less the length of MP1+2, radial cell closed, r3 vein absent, r4 vein reduced (not reaching the RP or the radial cell), those of the anterior anal and posterior anal sectors, absent (Fig. 8E). Legs: tarsomeres 1 and 2 of the prothoracic legs with a similar length and tarsomere 1 of meso- and metathoracic legs is longer than 2. ***Abdomen*.** Integument shiny, punctured, with long dense setae, sternite 7 with margin sinuate, sternite 8 with margin notched; aedeagus with three teeth at the inner apex of paramere (Fig. 9F–H).

#### Female and immatures.

Unknown.

#### Distribution.

Mexico: Yucatán and Quintana Roo (Fig. 3).

#### Additional material examined.

“MEXICO: Quintana Roo/ 19 km N Carrillo Puerto/ 18-VI-1990 blacklight trap/ coll. M.C. Thomas” “*Cenophengusgorhami*/ Det. V. Vega-Badillo 2019” (1) | FSCA.

### Cenophengus hnogamui

Taxon classificationAnimaliaColeopteraPhengodidae

Vega-Badillo et al. 2021

9C6A7A5D-1357-5E47-8AA3-9C229DB17B5A

Fig. 10A–H

Cenophengus hnogamui Vega-Badillo et al. 2021a: 224.

#### Type locality.

Hidalgo, Mexico.

#### Type material examined.

***Holotype*** ♂: “MEXICO: Hidalgo, Huasca de/ Ocampo, Rancho Santa Elena, / Presa San Carlos, 2430 m.a.s.l./ 20°08'04.5" N 98°30' 49.9" W. / 05/IX-03/X/2005. Trampa /Malaise. Col. A. Contreras / Meléndez y Reynoso” | CNIN. ***Paratypes*** ♂: same data | CNIN (2); CC-UAEH (1).

#### Remarks.

*Cenophengushnogamui* is sister to *C.munizi* (Vega-Badillo et al. 2021b), but can be distinguished by the length of the antennal rami and terminal maxillary palpomere. In *C.munizi*, the antennal rami are twice as long as respective antennomere, whereas in *C.hnogamui*, they are 1.5 times as long as the respective antennomere. Additionally, in *C.munizi*, the terminal maxillary palpomere is shorter than the preceding three combined, whereas in *C.hnogamui*, it is as long as the preceding three combined.

#### Diagnosis.

Integument smooth, long antennae more than twice the length of pronotum, antennal rami 1.5 times the respective antennomere and each elytron 4.7 times as long as wide with whitish colouration at the apex; aedeagus with three teeth at the inner apex of paramere.

#### Redescription.

**Figure 10.**
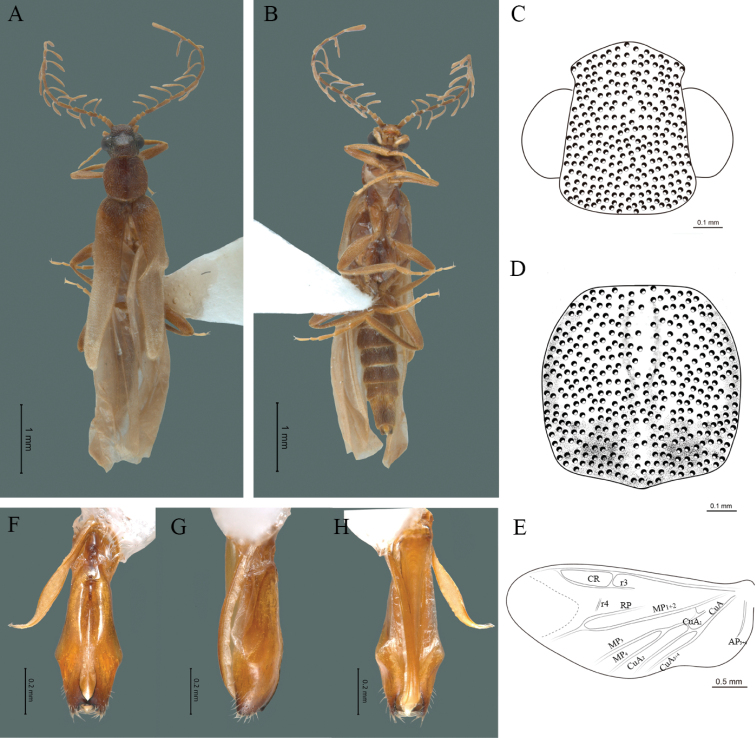
*Cenophengushnogamui* Vega-Badillo et al. 2021, male. Habitus: **A** dorsal **B** ventral **C** head dorsal **D** pronotum dorsal **E** hind wing. Wing venation: CR = Radial Cell; r3 = radial 3 vein; r4 = radial 4 vein; RP = Posterior Radial vein; MP1+2 = Posterior Median vein; CuA = Cubital vein; AA and AP = Anterior and Posterior Anal veins. Aedeagus: **F** dorsal view **G** lateral view **H** ventral view.

**Male.** Body length 4.12–4.5 mm; maximum body width 0.60–0.63 mm (pronotum). Body dark brown, except for first three antennomeres and posterior part of the elytra yellow-brown coloured (Fig. 10A, B). ***Head*.** Wider (0.6–0.63 mm) (0.62 ± 0.017 mm, n = 3) than long (0.37–0.43 mm) (0.4 ± 0.030 mm, n = 3) (Fig. 10C), at eye level, almost as wide as the pronotum, integument smooth, punctures twice as large as eye facets and separated by approximately 0.5 punctured diameters, each puncture bearing a yellow-brown seta; interantennal distance (0.09–0.11 mm) (0.1 ± 0.01 mm, n = 3) less than the length of antennomere 1; eyes 1/2 as long as head in lateral view, longer (0.24–0.26 mm) (0.25 ± 0.01 mm, n = 3) than wide (0.15–0.16 mm) (0.153 ± 0.057 mm, n = 3); interocular distance (0.3–0.33 mm) (0.32 ± 0.017 mm, n = 3) twice as long as eye width; long antennae (2.12–2.23 mm) (2.18 ± 0.058 mm, n = 3), more than twice the length of pronotum; antennomere 1 (0.15–0.16 mm) (0.153 ± 0.005 mm, n = 3) as long as the next two combined, antennomere 3 cup-shaped, the 4^th^ (0.10–0.11 mm) (0.103 ± 0.05 mm, n = 3) shorter than the following antennomeres; 5 to 11 about equal in length (0.21–0.23 mm) (0.22 ± 0.01 mm, n = 3, 12 (terminal) (0.28–0.31 mm) (0.29 ± 0.015 mm, n = 3), antennal rami lanceolate in lateral view, 1.5 times respective antennomere; terminal maxillary palpomere robust, securiform (0.18–0.21 mm) (0.21 ± 0.015 mm, n = 3), as long as the preceding three combined; terminal labial palpomere spindle-shaped (0.05–0.07 mm) (0.06 ± 0.01 mm, n = 3), 3 times as long as preceding one (0.02–0.03) mm (0.023 ± 0.015 mm, n = 3). ***Thorax*.** Pronotum longer (0.61–0.67 mm) (0.64 ± 0.03 mm, n = 3) than wide (0.60–0.65 mm) (0.64 ± 0.025 mm, n = 3) (Fig. 10D); integument smooth, punctures twice as large as eye facets and separated by approximately 1 punctured diameter, with a yellow-brown seta in each puncture; disc convex, posterior margin almost straight without middle notch, sides convex, anterior and posterior angles rounded; mesosternal suture complete; scutellum with posterior margin rounded; each elytron 4.7 times as long (2.0–2.3 mm) (2.14 ± 0.15 mm, n = 3) as wide (0.40–0.50 mm) (0.45 ± 0.052 mm, n = 3), convex, without longitudinal costae, elytral apex acute; hind wings with posterior radial vein (RP) length 2.2 times less than the length of MP1+2, radial cell closed, r3 vein presented, r4 vein reduced (not reaching the RP or the radial cell), those of the anterior anal and posterior anal sectors (Fig. 10E), evident. Legs: tarsomeres 1 and 2 of pro-, meso- and metathoracic legs with a similar length. ***Abdomen*.** Integument shiny, punctured, with long dense setae, sternite 7 with margin sinuate, sternite 8 with margin notched; aedeagus with three teeth at the inner apex of paramere (Fig. 10F–H).

#### Immatures and females.

Unknown.

#### Distribution.

Mexico: Hidalgo (Fig. 3).

### Cenophengus howdeni

Taxon classificationAnimaliaColeopteraPhengodidae

Zaragoza-Caballero, 1986

3F8D37B5-3446-5944-9C63-6154DACA0650

Fig. 11A–H

Cenophengus howdeni Zaragoza-Caballero, 1986: 933.

#### Type locality.

Durango, Mexico.

#### Type material examined.

***Holotype*** ♂: “MEXICO: Durango /24 ml. W. La Ciudad/ Dgo. MEX. VII. 11. 64/ H.F, Howden” | CNIN.

#### Remarks.

*Cenophengushowdeni* is morphologically similar to *C.tupae*, but can be distinguished by the length of antennomere 1, the pronotal disc and interocular distance. In *C.howdeni*, antennomere 1 shorter than next two combined, whereas in *C.tupae*, it is longer than next two combined. The pronotal disc with groove along mid-line in *C.howdeni*, in *C.tupae*, it has disc convex, weakly elevated dorsally forming a small depression in the basal part of each side. The interocular distance is 3 times eye width in *C.howdeni*, in *C.tupae*, it is twice as long as eye width.

#### Diagnosis.

Integument chagreened, antennae long, more than twice the length of pronotum, antennal rami twice as long as the respective antennomere, each elytron 2.6 times as long as wide; aedeagus with three teeth at the inner apex of paramere.

#### Redescription.

**Figure 11.**
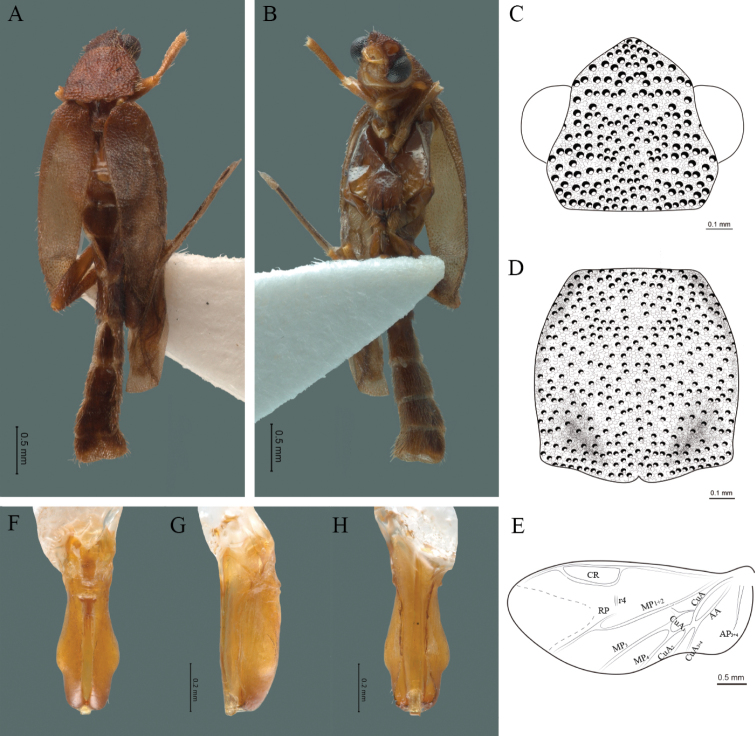
*Cenophengushowdeni* Zaragoza-Caballero, 1986, male. Habitus: **A** dorsal **B** ventral **C** head dorsal **D** pronotum dorsal **E** hind wing. Wing venation: CR = Radial Cell; r4 = radial 4 vein; RP = Posterior Radial vein; MP1+2 = Posterior Median vein; CuA = Cubital vein; AA and AP = Anterior and Posterior Anal veins. Aedeagus: **F** dorsal view **G** lateral view **H** ventral view.

**Male.** Body length 5.20 mm; maximum body width 0.60 mm (pronotum). Body brown, yellowish mandibles with darker tips (Fig. 11A, B). ***Head*.** Wider (0.67 mm) than long (0.36 mm) (Fig. 11C), at eye level, a wider than the pronotum, integument chagreened, punctures as long as eye facets and separated by approximately 1 punctured diameter, each puncture bearing a brown seta; interantennal distance (0.15 mm) wider than the length of antennomere 1 (0.10 mm); eyes 1/2 as long as head in lateral view, longer (0.45 mm) than wide (0.16 mm); interocular distance (0.45 mm) 3 times eye width; antennae long (1.64 mm) more than twice the length of pronotum; antennomere 1 (0.10 mm) shorter than next two combined (0.17 mm), antennomere 3 cup-shaped, 4 (0.12 mm) shorter than following antennomeres, 5 to 11 about equal in length (0.15 mm), 12 (terminal) (0.25 mm), antennal rami lanceolate in lateral view, twice as long as the respective antennomere; terminal maxillary palpomere robust, securiform (0.25 mm), as long as the preceding three combined (0.24 mm); terminal labial palpomere spindle-shaped (0.10 mm), 3 times as long as preceding one (0.03 mm). ***Thorax*.** Pronotum as long (0.74 mm) as wide (0.70 mm) (Fig. 11D); integument chagreened, punctures as long as eye facets and separated by approximately 2 punctured diameters, each puncture bearing a brown seta, disc convex, posterior magin curved with middle notch, sides almost straight, anterior angles rounded and posterior angles acute; mesosternal suture complete; scutellum with posterior margin rounded; each elytron 2.6 times as long (2.10 mm) as wide (0.80 mm), convex, without longitudinal costae, elytral apex obtuse; hind wings with posterior radial vein (RP) length 6 times less than the length of MP1+2, radial cell closed, r3 vein absent, r4 vein reduced (not reaching the RP or the radial cell), those of the anterior anal and posterior anal sectors (Fig. 11E). Legs: tarsomeres of the holotype lost. ***Abdomen*.** Integument shiny, punctured, with long dense setae, sternite 7 with margin sinuate, sternite 8 with margin notched; aedeagus with three teeth at the inner apex of paramere (Fig. 11F–H).

#### Female and immatures.

Unknown.

#### Distribution.

Mexico: Durango (Fig. 3).

### Cenophengus huatulcoensis

Taxon classificationAnimaliaColeopteraPhengodidae

Zaragoza-Caballero, 2008

675F5106-1094-5106-8281-05658B1DC9BB

Fig. 12A–H

Cenophengus huatulcoensis Zaragoza-Caballero, 2008: 154.

#### Type locality.

Oaxaca, Mexico.

#### Type material examined.

***Holotype*** ♂: “MEXICO: Oaxaca/ Parque Nal. Huatulco/ Estación el Sabanal/ 15° 48' 10" N, 98° 11' / 39.4"O. Alt. 109 m. / TL-1. 30/05/2005/ Col. S. Zaragoza” | CNIN. ***Paratype*** ♂: “MEXICO: Oaxaca/ Parque Nal. Huatulco/ Estación el Sabanal/ 15° 48' 10" N, 98° 11' / 39.4"O. Alt. 109 m. / TL-1. 30/05/2005/ Col. S. Zaragoza” (3) | CNIN; “MEXICO: Oaxaca-Parque Nal. Huatulco/ 1 km N Estación el Sabanal / TL-4. 15° 46' 10" N / 98° 11'40.6"O. 05-09-2005” “S. Zaragoza, F.A. Noguera/ E. Ramírez, E. González/ y V. Jiménez” (2) | CNIN.

#### Remarks.

*Cenophengushuatulcoensis* is morphologically similar to *C.baios*, but can be distinguished by its shorter size, interantennal and interocular distances. In *C.hualcoensis*, the interantennal distance is equal to the length of the antennomere 1, whereas in *C.baios*, it is shorter. The interocular distance is 3.0 times longer than eye width in *C.hualcoensis*, in *C.baios*, it is 3.5 times longer. Additionally, in *C.hualcoensis*, the antennal rami are twice as long as the respective antennomere, whereas in *C.baios*, they are as long as the respective antennomere.

#### Diagnosis.

Integument smooth, antennae less than twice the length of the pronotum, antennal rami lanceolate, twice as long as respective antennomere, pronotum as long as wide and each elytron 3.1 times as long as wide; aedeagus with one spine at the inner apex of paramere.

#### Redescription.

**Figure 12.**
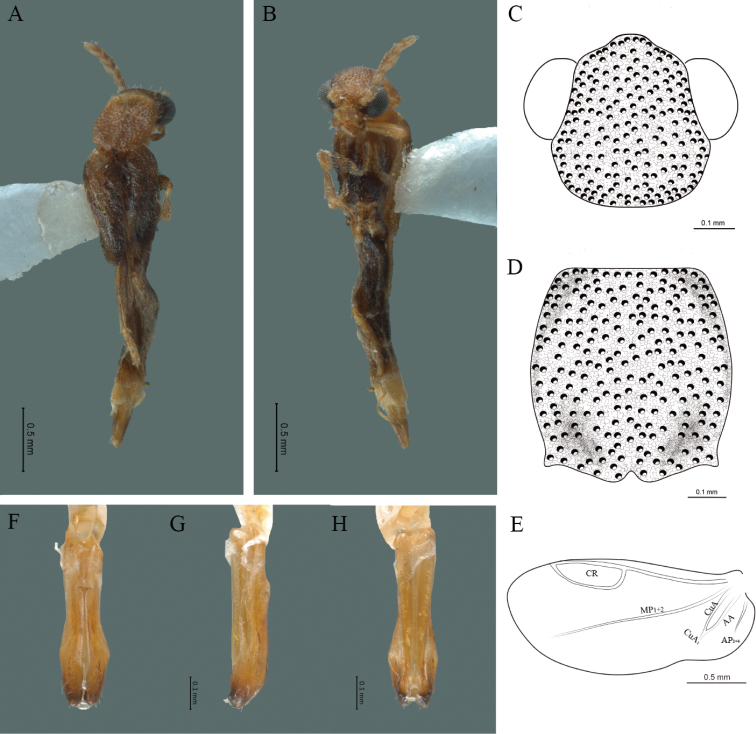
*Cenophengushautulcoensis* Zaragoza-Caballero, 2008, male. Habitus: **A** dorsal **B** ventral **C** head dorsal **D** pronotum dorsal **E** hind wing. Wing venation: CR = Radial Cell; MP1+2 = Posterior Median vein; CuA = Cubital vein; AA and AP = Anterior and Posterior Anal veins. Aedeagus: **F** dorsal view **G** lateral view **H** ventral view.

**Male.** Body length 2.84–3.0 mm; maximum body width 0.46–0.48 mm (pronotum). Body dark, except for anterior part of head, anterior half of pronotum, legs and seventh abdominal segment yellow (Fig. 12A, B). ***Head*.** Wider (0.50–0.55 mm) (0.525 ± 0.020 mm, n = 6) than long (0.28–0.32 mm) (0.3 ± 0.016 mm, n = 6) (Fig. 12C), at eye level, wider than the pronotum, integument smooth, punctures 2.5 times as long as eye facets and separated by approximately 1 punctured diameter, each puncture bearing a yellow-brown seta; interantennal distance (0.06–0.08 mm) (0.07 ± 0.008 mm, n = 6) equal to the length of antennomere 1 (0.07–0.08 mm) (0.073 ± 0.005 mm, n = 6); eyes 1/2 as long as head in lateral view, longer (0.18–0.21 mm) (0.19 ± 0.010 mm, n = 6) than wide (0.10–0.12 mm) (0.11 ± 0.008 mm, n = 6); interocular distance (0.29–0.31 mm) (0.29 ± 0.007 mm, n = 6) 3 times eye width; short antennae (0.82–0.90 mm) (0.83 ± 0.031 mm, n = 6), less than twice the length of the pronotum; antennomere 1 (0.07–0.08 mm) (0.073 ± 0.005 mm, n = 6) shorter than the next two combined, antennomere 3 cup-shaped, 4 to 11 about equal in length (0.07–0.08 mm) (0.071 ± 0.004 mm, n = 6), 12 (terminal) (0.1–0.11 mm) (0.101 ± 0.004 mm, n = 6), antennal rami lanceolate in lateral view, twice as long as respective antennomere; terminal maxillary palpomere robust, securiform (0.12–0.13 mm) (0.121 ± 0.004 mm, n = 6), is shorter than the preceding three combined; terminal labial palpomere spindle-shaped (0.05–0.07 mm) (0.056 ± 0.008 mm, n = 6), 2.5 times as long as preceding one (0.02–0.03 mm) (0.025 ± 0.054 mm, n = 6). ***Thorax*.** Pronotum as long (0.47–0.51 mm) (0.48 ± 0.018 mm, n = 6) as wide (0.47–0.52 mm) (0.5 ± 0.018 mm, n = 6) (Fig. 12D); integument smooth, punctures 2.5 times as long as eye facets and separated by approximately 1 punctured diameter, each puncture bearing a yellow-brown seta, disc convex, posterior margin curved, sides curved, anterior angles rounded and posterior acute; mesosternal suture incomplete; scutellum with small notch on posterior margin; each elytron 3.1 times as long (0.80–0.90 mm) (0.85 ± 0.035 mm, n = 6) as wide (0.25–0.28 mm) (0.275 ± 0.012 mm, n = 6), convex, without longitudinal costae, elytral apex right angled; posterior hind wings with posterior radial vein (RP) absent, radial cell closed and slightly defined, r3 and r4 vein absent, those of the anterior anal and posterior anal sectors, evident (Fig. 12E). Legs: tarsomeres 1 and 2 of the prothoracic legs with a similar length and tarsomere 1 of meso- and metathoracic legs is longer than 2. ***Abdomen*.** Integument shiny, punctured, with long dense setae, sternite 7 with margin sinuate, sternite 8 with margin notched; aedeagus with one spine at the inner apex of paramere (Fig. 12F–H).

#### Immatures and females.

Unknown.

#### Distribution.

Mexico: Oaxaca (Fig. 3).

### Cenophengus kikapu

Taxon classificationAnimaliaColeopteraPhengodidae

Vega-Badillo et al. 2021

8A5BC840-D5D8-57A4-B559-9CB32E25FEAA

Fig. 13A–H

Cenophengus kikapu Vega-Badillo et al. 2021a: 227.

#### Type locality.

Coahuila, Mexico.

#### Type material examined.

***Holotype*** ♂: “MEXICO: El Cañón, Cuatro/ Ciénegas, Coahuila, Col. MTO/ y UOGV 21/feb/2012 Col. / nocturna luz blanca” |CNIN. ***Paratypes*** ♂: same data |CNIN (2).

#### Remarks.

*Cenophenguskikapu* is morphologically similar to *C.sonoraensis*, but can be distinguished by the width of the head and the terminal maxillary palpomere. In *C.sonoraensis*, the head is almost as wide as the pronotum, whereas in *C.kikapu*, the head is wider than the pronotum. In addition, the terminal maxillary palpomere is as long as the preceding three combined in *C.sonoraensis*, whereas in *C.kikapu*, it is longer than the preceding three combined.

#### Diagnosis.

Head almost as wide as pronotum, integument chagreened, antennae less than twice the length of the pronotum, antennal rami twice as long as respective antennomere, terminal maxillary palpomere as long as preceding three combined, each elytron 3.6 times as long as wide; aedeagus with three teeth at the inner apex of paramere.

#### Redescription.

**Figure 13.**
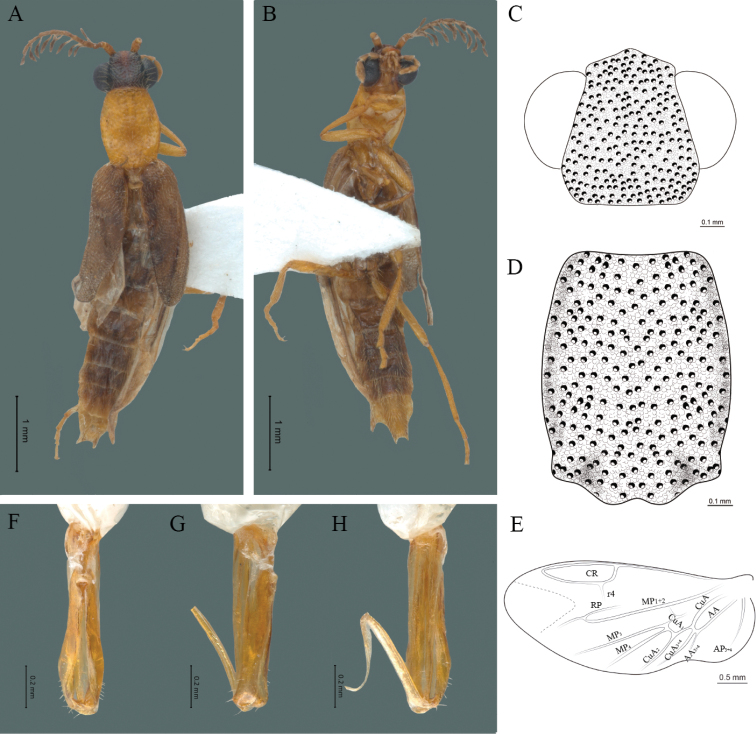
*Cenophenguskikapu* Vega-Badillo et al. 2021, male. Habitus: **A** dorsal **B** ventral **C** head dorsal **D** pronotum dorsal **E** hind wing. Wing venation: CR = Radial Cell; r4 = radial 4 vein; RP = Posterior Radial vein; MP1+2 = Posterior Median vein; CuA = Cubital; AA and AP = Anterior and Posterior Anal veins. Aedeagus: **F** dorsal view **G** lateral view **H** ventral view.

**Male.** Body length 4.64–5.0 mm; maximum body width 0.80–0.82 mm (pronotum). Body dark brown, except for pronotum, legs and two last abdominal segments yellow-orange (Fig. 13A, B). ***Head*.** Wider (0.80–0.81 mm) (0.806 ± 0.005 mm, n = 3) than long (0.50–0.52 mm) (0.506 ± 0.011 mm, n = 3) (Fig. 13C), at eye level, almost as wide as pronotum, integument chagreened, punctures 1.5 times as long as eye facets and separated by approximately 2 punctured diameters, each puncture bearing a yellow-brown seta; interantennal distance (0.07–0.09 mm) (0.08 ± 0.01 mm, n = 3) less than the length of antennomere 1 (0.16–0.18 mm) (0.17 ± 0.01 mm, n = 3); eyes 3/4 as long as head in lateral view, longer (0.35–0.37 mm) (0.356 ± 0.011 mm, n = 3) than wide (0.19–0.20 mm) (0.196 ± 0.005 mm, n = 3); interocular distance (0.40–0.42 mm) (0.41 ± 0.01 mm, n = 3) twice eye width; antennae short (1.63–1.73 mm) (1.68 ± 0.05 mm, n = 3) less than twice the length of the pronotum; antennomere 1 (0.16–0.18 mm) (0.17 ± 0.01 mm, n = 3) is longer than the next two combined, antennomere 3 cup-shaped, 4 (0.11–0.12 mm) (0.116 ± 0.005 mm, n = 3) shorter than the following antennomeres, 5 to 11 about equal in length (0.15–0.17 mm) (0.16 ± 0.01 mm, n = 3), 12 (terminal) (0.20–0.21 mm) (0.203 ± 0.005 mm, n = 3), antennal rami lanceolate in lateral view, twice as long as the respective antennomere; terminal maxillary palpomere robust, securiform (0.28–0.30 mm) (0.286 ± 0.011 mm, n = 3), as long as the preceding three combined; terminal labial palpomere spindle-shaped (0.05–0.06 mm) (0.056 ± 0.005 mm, n = 3), 3 times as long as preceding one (0.02–0.03 mm) (0.26 ± 0.005 mm, n = 3). ***Thorax*.** Pronotum longer (1.07–1.09 mm) (1.073 ± 0.015 mm, n = 3) than wide (0.80–0.82 mm) (0.81 ± 0.01 mm, n = 3) (Fig. 13D); integument chagreened, punctures 1.5 times as long as eye facets and separated by approximately 2 punctured diameters, each puncture bearing a yellow-brown seta, disc convex, weakly elevated dorsally forming a small depression in the basal part of each side, posterior margin almost straight with a middle notch, sides almost straight, anterior and posterior angles rounded; mesosternal suture complete; scutellum with posterior margin rounded; each elytron 3.6 times as long (1.7–2.0 mm) (1.89 ± 0.015 mm, n = 3) as wide (0.44–0.50 mm) (0.46 ± 0.030 mm, n = 3), convex, without longitudinal costae, elytral apex rounded; hind wings with posterior radial vein (RP) length 3.5 times less than the length of MP1+2, radial cell closed, r3 vein absent, r4 vein developed (reaching the radial cell), those of the anterior anal and posterior anal sectors, evident (Fig. 13E). Legs: tarsomeres 1 and 2 of pro-, meso- and metathoracic legs with a similar length. ***Abdomen*.** Integument shiny, punctured, with long dense setae, sternite 7 with margin sinuate, sternite 8 with margin notched; aedeagus with three teeth at the inner apex of paramere (Fig. 13F–H).

#### Female and immatures.

Unknown.

#### Distribution.

Mexico: Coahuila (Fig. 3).

### Cenophengus longicollis

Taxon classificationAnimaliaColeopteraPhengodidae

Wittmer, 1976

FA4F735E-0F2D-5638-9AEE-E60AF35456EC

Fig. 14A–H

Cenophengus longicollis Wittmer, 1976: 451.

#### Type locality.

Texas, USA.

#### Type material examined.

***Holotype*** ♂: “U.S. A: 3 mi. NE. of / Porvenir/ Presidio Co. /Tex. 26.IX.46. / B. Patterson, / J. M. SchmidI” “California Academy / of Sciences /Type No. 12986”. |FMNH. ***Paratype*** ♂: “Texas: Jeff Davis Co. /Ft. Davis, Limpia Cayon/ 16.VII.1964 St. Pla” “At light/ W. Suter leg.” (1) |FMNH. “New Mexico: White´s City/ Eddy Co. 8. IX. 1952” “C.N.H.M 1960/ Borys Malkin/ Coleoptera Colln.” (1) |FMNH.

#### Remarks.

*Cenophenguslongicollis* is morphologically similar to *C.xiinbali*, but can be distinguished by the interocular distance and the terminal maxillary palpomere. In *C.longicollis*, the interocular distance is 3.5 times longer than eye width, whereas in *C.xiinbali*, it is 2.5 times longer. The terminal maxillary palpomere is longer than the preceding three combined in *C.longicollis*, whereas in *C.xiinbali*, it is as long as the preceding three combined.

#### Diagnosis.

Integument chagreened, antennae less than twice the length of the pronotum, antennal rami twice as long as the respective antennomere and each elytron 3.5 times as long as wide; aedeagus with three teeth at the inner apex of paramere.

#### Redescription.

**Figure 14.**
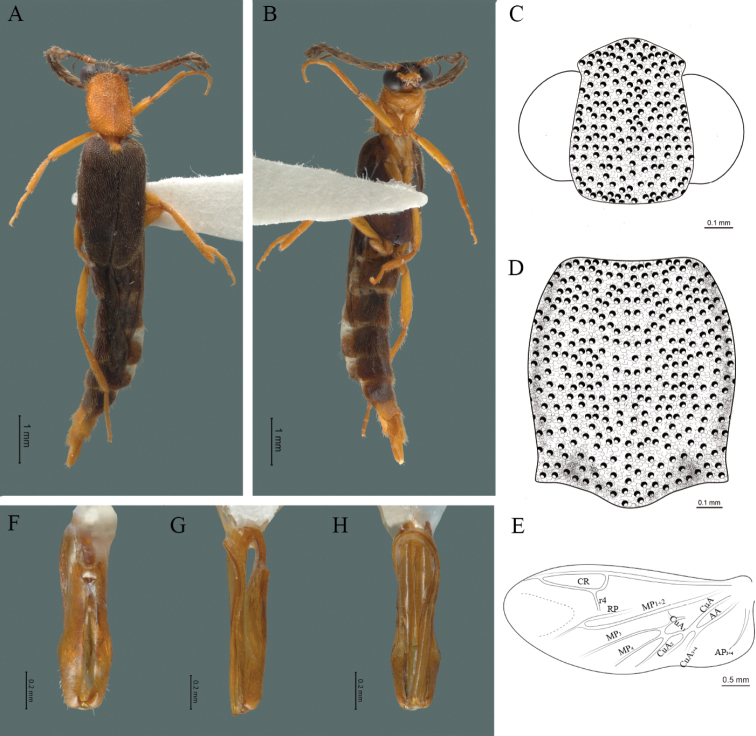
*Cenophenguslongicollis* Wittmer, 1976, male. Habitus: **A** dorsal **B** ventral **C** head dorsal **D** pronotum dorsal **E** hind wing. Wing venation: CR = Radial Cell; r4 = radial 4 vein; RP = Posterior Radial vein; MP1+2 = Posterior Median vein; CuA = Cubital vein; AA and AP = Anterior and Posterior Anal veins. Aedeagus: **F** dorsal view **G** lateral view **H** ventral view.

**Male.** Body length 5.6–7.2 mm; maximum body width 0.82–1.0 mm (pronotum). Head black; antennae black to brown, pronotum and scutellum yellow-orange; wingtips black to brown, sometimes only at the base poorly lit, legs and lower yellow to yellow-orange (Fig. 14A, B). ***Head*.** Wider (0.83–1.0 mm) (0.91 ± 0.074 mm, n = 4) than long (0.5–0.6 mm) (0.53 ± 0.038 mm, n = 4) (Fig. 14C), at eye level, a little wider than the pronotum, integument chagreened, punctures 2.5 times as long as eye facets and separated by approximately 1 punctured diameter, each puncture bearing a brown seta; interantennal distance (0.09–0.11 mm) (0.09 ± 0.010 mm, n = 4) less than the length of antennomere 1 (0.17–0.23 mm) (0.20 ± 0.026 mm, n = 4); eyes 3/4 as long as head in lateral view, longer (0.36–0.46 mm) (0.39 ± 0.034 mm, n = 4) than wide (0.20–0.25 mm) (0.22 ± 0.022 mm, n = 4); interocular distance (0.42–0.5 mm) (0.46 ± 0.033 mm, n = 4) 3.5 times eye width; antennae short (1.75–2.21 mm) (0.22 ± 0.014 mm, n = 4) less than twice the length of the pronotum; antennomere 1 (0.17–0.23 mm) (0.20 ± 0.026 mm, n = 4) a little longer than next two combined, antennomere 3 cup-shaped, 4 (0.15–0.17 mm) (0.16 ± 0.01 mm, n = 4) shorter than the following antennomeres, 5 to 11 about equal in length (0.16–0.20 mm) (0.178 ± 0.017 mm, n = 4), 12 (terminal) (0.20–0.30 mm) (0.22 ± 0.037 mm, n = 4), antennal rami lanceolate in lateral view, twice as long as the respective antennomere; terminal maxillary palpomere robust, securiform (0.25–0.30 mm) (0.27 ± 0.023 mm, n = 4), is longer than the preceding three combined; terminal labial palpomere spindle-shaped (0.10–0.11 mm) (0.105 ± 0.01 mm, n = 4), 3.5 times as long as preceding one (0.03–0.04 mm) (0.35 ± 0.005 mm, n = 4). ***Thorax*.** Pronotum longer (1.01–1.20 mm) (1.12 ± 0.088 mm, n = 4) than wide (0.82–1.0 mm) (0.9 ± 0.080 mm, n = 4) (Fig. 14D); integument chagreened, punctures twice as large as eye facets and separated by approximately 1.5 punctured diameters, each puncture bearing a yellow-brown seta, convex disc, weakly elevated dorsally forming a small depression in the basal part of each side, posterior margin curved, sides almost straight, anterior angles rounded and posterior angles acute; mesosternal suture complete; scutellum with posterior margin rounded; each elytron 3.5 times as long (1.72–2.04 mm) (2.03 ± 0.263 mm, n = 4) as wide (0.52–0.65 mm) (0.59 ± 0.071 mm, n = 4), convex, without longitudinal costae, elytral apex rounded; hind wings with posterior radial vein (RP) length 3 times less than the length of MP1+2, radial cell closed, r3 vein absent, r4 vein developed (reaching the radial cell), those of the anterior anal and posterior anal sectors, evident (Fig. 14E). Legs: tarsomere 1 of pro-, meso- and metathoracic legs is longer than 2. ***Abdomen*.** Integument shiny, punctured, with long dense setae, sternite 7 with margin sinuate, sternite 8 with margin notched; aedeagus with three teeth at the inner apex of paramere (Fig. 14F–H).

#### Female and immatures.

Unknown.

#### Distribution.

USA: Texas (Fig. 3).

#### Additional material examined.

“U.S.A: Texas J. Davis/ Limpia Cyn. / July 26 197/ J. E. Wappes” **(1)** |FMNH.

### Cenophengus magnus

Taxon classificationAnimaliaColeopteraPhengodidae

Zaragoza-Caballero, 1988

5CD5D5D5-7018-528D-A915-931FCBBAD646

Fig. 15A–H

Cenophengus magnus Zaragoza-Caballero, 1988: 651.

#### Type locality.

Mexico.

#### Type material.

***Holotype*** ♂: “Nuevo Leon, Mexico (92°44'N; 99°56'W), 16 de Julio de 1979, 1 800 m, Col. D.C. Darling” | CUIC.

#### Remarks.

*Cenophengusmagnus* is sister to *C.major* (Vega-Badillo et al. 2021b), but can be distinguished by the elytral length and r3 vein. In *C.magnus*, each elytron is 4 times as long as wide, whereas in *C.major*, they are almost 4.5 times as long as wide; the r3 vein is absent in *C.magnus*, whereas in *C.major*, it is present.

#### Diagnosis.

Integument smooth, antennae long, more than twice the length of pronotum antennal rami, 3 times the respective antennomere, scutellum almost quadrangular, with small notch on posterior margin and each elytron 4 times as long as wide, with one longitudinal costa; aedeagus with three teeth at the inner apex of paramere.

#### Redescription.

**Figure 15.**
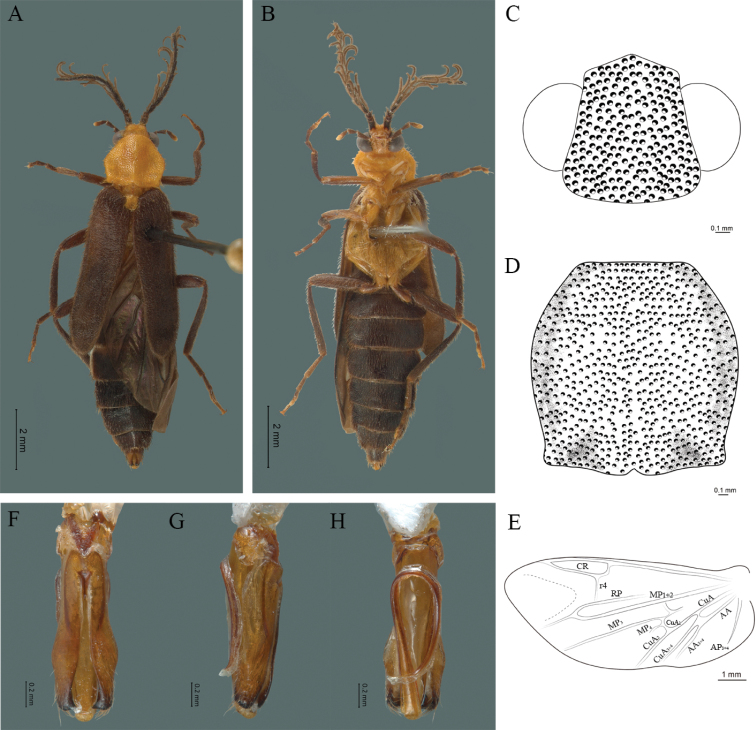
*Cenophengusmagnus* Zaragoza-Caballero, 1988, male. Habitus: **A** dorsal **B** ventral **C** head dorsal **D** pronotum dorsal **E** hind wing. Wing venation: CR = Radial Cell; r4 = radial 4 vein; RP = Posterior Radial vein; MP1+2 = Posterior Median vein; CuA = Cubital vein; AA and AP = Anterior and Posterior Anal veins. Aedeagus: **F** dorsal view **G** lateral view **H** ventral view.

**Male.** Body length 11.0–12.0 mm; maximum body width 1.74–1.80 mm (pronotum). Body brown, except for head, pronotum and scutellum yellow-orange; antennae and buccal parts dark brown (Fig. 15A, B). ***Head*.** Wider (1.30–1.35 mm) (1.325 ± 0.035 mm, n = 2) than long (0.75–0.80 mm) (0.77 ± 0.035 mm, n = 2) (Fig. 15C), at eye level, less wide than the pronotum, integument smooth, punctures 2.5 times as long as eye facets and separated by approximately 0.5 punctured diameters, each puncture bearing a yellow-orange seta; interantennal distance (0.14–0.16 mm) (0.15 ± 0.014 mm, n = 2) less than the length of antennomere 1 (0.31–0.34 mm) (0.325 ± 0.021 mm, n = 2); eyes 3/4 as long as head in lateral view , longer (0.55–0.6 mm) (0.57 ± 0.035 mm, n = 2) than wide (0.3–0.35 mm) (0.325 ± 0.035 mm, n = 2); interocular distance (0.65–0.7 mm) (0.675 ± 0.035 mm, n = 2) 1.9 times eye width; antennae long (4.02–4.27 mm) (4.14 ± 0.176 mm, n = 2) more than twice the length of pronotum; antennomere 1 (0.31–0.34 mm) (0.325 ± 0.021 mm, n = 2) longer than next two combined, antennomere 3 cup-shaped, 4 (0.30–0.33 mm) (0.315 ± 0.3 mm, n = 2) shorter than following antennomeres, 5 to 11 about equal in length (0.38–0.40 mm) (0.39 ± 0.014 mm, n = 2), 12 (terminal) (0.50–0.55 mm) (0.525 ± 0.035 mm, n = 2), antennal rami lanceolate in lateral view, 3 times the respective antennomere; terminal maxillary palpomere robust, securiform (0.35–0.40 mm) (0.375 mm ± 0.035 mm, n = 2), twice as short as the preceding three combined; terminal labial palpomere spindle-shaped (0.16–0.17 mm) (0.165 ± 0.007 mm, n = 2), 3 times as long as preceding one (0.05–0.07 mm) (0.06 ± 0.014 mm, n = 2). ***Thorax*.** Pronotum longer (1.9–2.0 mm) (1.95 ± 0.070 mm, n = 2) than wide (1.74–1.80 mm) (1.77 ± 0.042 mm, n = 2) (Fig. 15D); integument smooth, punctures twice as large as eye facets and separated by approximately 1 punctured diameter, each puncture bearing a yellow-orange seta, convex disc, weakly elevated dorsally forming a small depression in the basal part of each side, posterior margin curved, sides almost straight, anterior and posterior angles rounded; mesosternal suture incomplete; scutellum with small notch on posterior margin; each elytron 4 times as long (4.52–4.80 mm) (4.66 ± 0.197 mm, n = 2) as wide (1.1–1.2 mm) (1.15 ± 0.070 mm, n = 2), convex, with one longitudinal costae, elytral apex rounded; hind wings with posterior radial vein (RP) length twice less than the length of MP1+2, radial cell closed, r3 vein absent, r4 vein developed (reaching the RP and the radial cell), those of the anterior anal and posterior anal sectors, evident (Fig. 15E). Legs: tarsomeres 1 and 2 of the prothoracic legs with a similar length and tarsomere 1 of meso- and metathoracic legs is longer than 2. ***Abdomen*.** Integument shiny, punctured, with long dense setae, sternite 7 with margin concave, sternite 8 with margin rounded; aedeagus with three teeth at the inner apex of paramere (Fig. 15F–H).

#### Female and immatures.

Unknown.

#### Distribution.

Mexico: Nuevo León and Tamaulipas (Fig. 3).

#### Additional material examined.

“Mexico: Tamaulipas/ Gómez Farías km 7 a Julilo/ 13-IV-2003 /I. Pacheco L. Cervantes” “*Cenophengus /magnus* /S. Zaragoza C. det.” (2) | CNIN.

### Cenophengus major

Taxon classificationAnimaliaColeopteraPhengodidae

Wittmer, 1976

C12987A8-8B0B-535C-84E9-0369CB1E28A9

Fig. 16A–H

Cenophengus major Wittmer, 1976: 450
Cenophengus guerrerensis Zaragoza-Caballero, 1991: 109, syn. nov.

#### Type locality.

Nayarit, Mexico.

#### Type material examined.

***Holotype*** ♂: Mexico: “Tepic, Nayarit, / Mex. VII-28-53” “D. Rockefeller/ Mex. Exp. 1953/ C. & P. Vaurie” “*Cenophengusmajor* Wittmer” “Holotypus”. |AMNH. Holotype ♂: MEXICO: “Guerrero, Cerro Tuxpan/ Iguala, 12-VII-88. 8-2 pm. Col. R. Sánchez 11617” “*Cenophengus/ guerrerensis* /Zaragoza” | CNIN. ***Paratypes* ♂ (6)**: “Cerro Tuxpan/ Iguala, Gro. /1700 m. / 25-VI-87 /Col. R. Sánchez” | CNIN.

#### Remarks.

We synonymise *C.guerrerensis* with *C.major*, based on the observation of holotypes, being the body shape and total body length, as well as the maxillary palps and wing venation particularly important characters for its synonymisation. *C.major* is sister to *C.magnus* (Vega-Badillo et al. 2021b), but can be distinguished by the elytral length and r3 vein. In *C.magnus*, each elytron is 4 times as long as wide, whereas in *C.major*, they are almost 4.5 times as long as wide; the r3 vein is absent in *C.magnus*, whereas in *C.major*, it is present.

#### Diagnosis.

Integument smooth, antennae long, more than twice the length of pronotum, antennal rami lanceolate in lateral view, 3.1 times the respective antennomere and each elytron 4.5 times as long as wide; aedeagus with three teeth at the inner apex of paramere.

#### Redescription.

**Figure 16.**
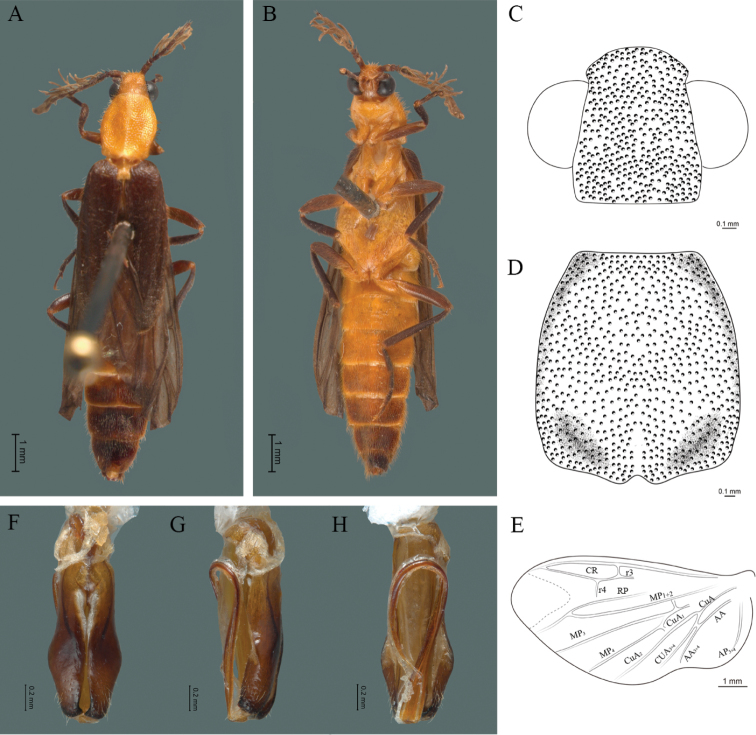
*Cenophengusmajor* Wittmer, 1976, male. Habitus: **A** dorsal **B** ventral **C** head dorsal **D** pronotum dorsal **E** hind wing. Wing venation: CR = Radial Cell; r3 = radial 3 vein; r4 = radial 4 vein; RP = Posterior Radial vein; MP1+2 = Posterior Median vein; CuA = Cubital vein; AA and AP = Anterior and Posterior Anal veins. Aedeagus: **F** dorsal view **G** lateral view **H** ventral view.

**Male.** Body length 10.0–13.0 mm; maximum body width 1.33–1.64 mm (pronotum). Body brown, except for head, pronotum and scutellum yellow-orange; antennae and buccal parts dark brown (Fig. 16A, B). ***Head*.** Wider (1.13–1.47 mm) (1.35 ± 0.117 mm, n = 8) than long (0.65–0.72 mm) (0.692 ± 0.0218 mm, n = 8) (Fig. 16C), at eye level, less wide than the pronotum, integument smooth, punctures twice as large as eye facets and separated by approximately 1 punctured diameter, each puncture bearing a yellow-orange seta; interantennal distance (0.11–0.16 mm) (0.15 ± 0.017 mm, n = 8) less than the length of antennomere 1 (0.35–0.40 mm) (0.353 ± 0.028 mm, n = 8); eyes 3/4 as long as head in lateral view, longer (0.51–0.58 mm) (0.54 ± 0.021 mm, n = 8) than wide (0.28.0.38 mm) (0.33 ± 0.035 mm, n = 8); interocular distance (0.66–0.75 mm) (0.70 ± 0.035 mm, n = 8) 1.8 times eye width; antennae long (3.40–4.03 mm) (3.8 ± 0.168 mm, n = 8), more than twice the length of pronotum; antennomere 1 (0.35–0.40 mm) (0.353 ± 0.028 mm, n = 8) longer than next two combined, antennomere 3 cup-shaped, 4 (0.30–0.40 mm) (0.38 ± 0.035 mm, n = 8) shorter than following antennomeres, 5 to 11 about equal in length (0.33–0.40 mm) (0.35 ± 0.025 mm, n = 8), 12 (terminal) (0.50–0.55 mm) (0.52 ± 0.24 mm, n = 8), antennal rami lanceolate in lateral view, 3 times the respective antennomere; terminal maxillary palpomere robust, securiform (0.38–0.46 mm) (0.42 ± 0.029 mm, n = 8), shorter than the preceding three combined; terminal labial palpomere spindle-shaped (0.25–0.27 mm) (0.22 ± 0.030 mm, n = 8), twice as long as preceding one (0.10–0.12 mm) (0.102 ± 0.007 mm, n = 8). ***Thorax*.** Pronotum longer (1.65–1.84 mm) (1.71 ± 0.071 mm, n = 8) than wide (1.33–1.64 mm) (1.45 ± 0.122 mm, n = 8) (Fig. 16D); integument smooth, punctures twice as large as eye facets and separated by approximately 1.5 punctured diameters coarsely punctured, each puncture bearing a yellow-orange seta, convex disc, weakly elevated dorsally forming a small depression in the basal part of each side, posterior margin curved with middle notch, sides almost straight, anterior and posterior angles rounded; mesosternal suture incomplete; scutellum with posterior margin rounded; each elytron 4.5 times as long (4.0–5.0 mm) (4.59 ± 0.332 mm, n = 8) as wide (0.92–1.16 mm) (1.05 ± 0.084 mm, n = 8), convex, with one longitudinal costae, elytral apex rounded; hind wings with posterior radial vein (RP) length twice less than the length of MP1+2, radial cell closed, r3 vein present, r4 vein developed (reaching the radial cell), those of the anterior anal and posterior anal sectors, evident (Fig. 16E). Legs: tarsomere 1 of pro-, meso- and metathoracic legs is longer than 2. ***Abdomen*.** Integument shiny, punctured, with long dense setae, sternite 7 with margin sinuate, sternite 8 with margin rounded; aedeagus with three teeth at the inner apex of paramere (Fig. 16F–H).

#### Female and immatures.

Unknown.

#### Distribution.

Mexico: Nayarit, Guerrero, and Hidalgo (Fig. 3).

#### Additional material examined.

“MEXICO: Hidalgo: PN Los Mármoles/ Minas Viejas, Bosque de encino/ 1892 m. N 20° 55' W 99° 12' 41.1”/ Trampa de luz 18-VIII-2007/ J. Márquez, J. Asiain y S. Sierra cols.” | CNIN.

### Cenophengus marmoratus

Taxon classificationAnimaliaColeopteraPhengodidae

Wittmer, 1976

F4857074-95C1-54F0-9D25-B653C0BE645A

Fig. 17A–H

Cenophengus marmoratus Wittmer, 1976: 453.

#### Type locality.

Veracruz, Mexico.

#### Type material examined.

***Holotype*** ♂: Mexico: “Cordoba/ Mex. Ver. / Dr. A. Fenyes” “*Cenophengus/ mamoratus*/ Wittmer” “Type No./ 73886/ USMN” | NMNH.

#### Remarks.

*Cenophengusmarmoratus* is morphologically similar to *C.wittmeri*, but can be distinguished by the colour of the body and the terminal maxillary palpomere. In *C.marmoratus*, the body is yellow or pale brown, the pronotum is partially interrupted by darker brown spots, whereas in *C.wittmeri*, they are brown, except for the middle part of the pronotum that is dark brown. The terminal maxillary palpomere is shorter than the preceding three combined in *C.marmoratus*, in *C.wittmeri*, it is as long as the preceding three combined. Additionally, in *C.marmoratus*, the posterior radial vein (RP) length is 1.6 times less than the length of MP1+2, whereas in *C.wittmeri*, it is twice less than the length of MP1+2.

#### Diagnosis.

Head almost as wide as the pronotum, integument chagreened, antennae long, more than twice the length of pronotum, antennal rami twice as long as the respective antennomere; pronotum mottled with darker brown spots; each elytron 4.0 times as long as wide; aedeagus with three teeth at the inner apex of paramere.

#### Redescription.

**Figure 17.**
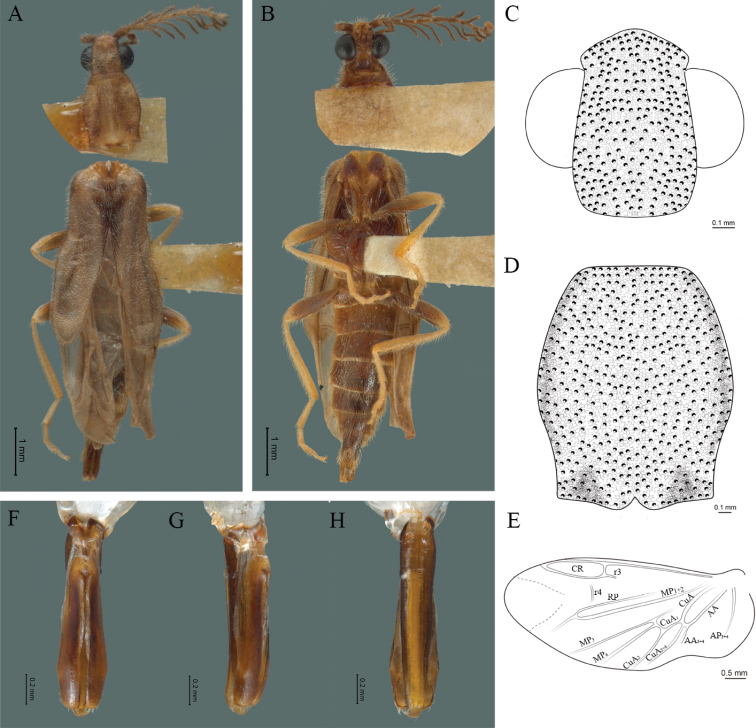
*Cenophengusmarmoratus* Wittmer, 1976, male. Habitus: **A** dorsal **B** ventral **C** head dorsal **D** pronotum dorsal **E** hind wing. Wing venation: CR = Radial Cell; r3 = radial 3 vein; r4 = radial 4 vein; RP = Posterior Radial vein; MP1+2 = Posterior Median vein; CuA = Cubital vein; AA and AP = Anterior and Posterior Anal veins. Aedeagus: **F** dorsal view **G** lateral view **H** ventral view.

**Male.** Body length 7.9–10.3 mm; maximum body width 0.99–1.20 mm (pronotum). Body yellow or pale brown; antennal rami somewhat darker than respective antennomere, pronotum mottled with darker brown spots (Fig. 17A, B). ***Head*.** Wider (0.86–1.10 mm) (0.1 ± 0.085, n = 6) than long (0.60–0.75 mm) (0.66 ± 0.051 mm, n = 6) (Fig. 17C), at eye level, almost as wide as the pronotum, integument chagreened, punctures 1.5 times as long as eye facets and separated by approximately 2 punctured diameters, each puncture bearing a yellow-brown seta; interantennal distance (0.09–0.10 mm) (0.096 ± 0.005 mm, n = 6) less than the length of antennomere 1 (0.24–0.30 mm) (0.28 ± 0.025 mm, n = 6); eyes 3/4 as long as head in lateral view, longer (0.42–0.55 mm) (0.473 ± 0.045 mm, n = 6) than wide (0.20–0.27 mm) (0.24 ± 0.025 mm, n = 6); interocular distance (0.45–0.60 mm) (0.52 ± 0.057 mm, n = 6) 2.1 times longer than eye width; antennae long (2.62–3.0 mm) (2.74 ± 0.13 mm, n = 6) more than twice the length of pronotum; antennomere 1 (0.24–0.30 mm) (0.28 ± 0.025 mm, n = 6) longer than next two combined, antennomere 3 cup-shaped, 4 (0.20–0.25 mm) (0.22 ± 0.024 mm, n = 6) shorter than the following antennomeres, 5 to 11 about equal in length (0.24–0.28 mm) (0.25 ± 0.016 mm, n = 6), 12 (terminal) (0.33–0.38 mm) (0.35 ± 0.018 mm, n = 6), antennal rami lanceolate in lateral view, twice as long as the respective antennomere; terminal maxillary palpomere robust, securiform (0.31–0.36 mm) (0.33 ± 0.024 mm, n = 6), shorter than the preceding three combined; terminal labial palpomere spindle-shaped (0.15–0.20 mm) (0.17 ± 0.025 mm, n = 6), twice as long as preceding one (0.05–0.08 mm) (0.07 ± 0.015 mm, n = 6). ***Thorax*.** Pronotum longer (1.30–1.54 mm) (0.136 ± 0.09 mm, n = 6) than wide (0.92–1.22 mm) (1.1 ± 0.123 mm, n = 6) (Fig. 17D); integument chagreened, punctures 1.5 times as long as eye facets and separated by approximately 2 punctured diameters, each puncture bearing a yellow-brown seta, convex disc, weakly elevated dorsally forming a small depression in the basal part of each side, posterior margin curved with middle notch, sides almost straight, anterior angles rounded and posterior angles acute; mesosternal suture complete; with scutellum posterior margin rounded; each elytron 4.0 times as long (2.50–3.70 mm) (3.08 ± 0.458 mm, n = 6) as wide (0.68–0.94 mm) (0.77 ± 0.116 mm, n = 6), convex, without longitudinal costae, elytral apex rounded; hind wings posterior radial vein (RP) length 1.6 times less than the length of MP1+2, radial cell closed, r3 vein present, r4 vein reduced (not reaching the RP or the radial cell), those of the anterior anal and posterior anal sectors, evident (Fig. 17E). Legs: tarsomere 1 of pro-, meso- and metathoracic legs is longer than 2. ***Abdomen*.** Integument shiny, punctured, with long dense setae, sternite 7 with margin sinuate, sternite 8 with margin notched; aedeagus with three teeth at the inner apex of paramere (Fig. 17F–H).

#### Female and immatures.

Unknown.

#### Distribution.

Mexico: Veracruz, Hidalgo, San Luis Potosí, and Querétaro (Fig. 3).

#### Additional material examined.

“Mexico: Hidalgo/: km 14 Carr. Huejutla- /Atlepexco 13-05-1999/ H. Brailovsky y E. Barrera” (1) |CNIN “MEXICO: Hidalgo, Cuautepec /Tezoncualpan “El Caminero” / Bosque de encino. / N 19° 56' 53.8" W 98° 16' 27.9". /Trampa intercepción de vuelo, / 22 a 29- VIII-2009, M. Torres col.” (1) | CNIN; “San Luis Potosí: /Rio Micos /9-IV-78 / Col S.Z.C.” (2) | CNIN; “MEXICO: San Luis Potosí /Xilitla, Los Pozos/ 21 22 45 N 99 00 15 O/ 780 m.a.s.l. 03-VII-2006/ L. Cervantes, D. Brzoska” (1) | CNIN; “Mexico: Querétaro: / Misión de Bucareli, / N 21° 02' 280"/ O 99° 36' 885"/ 1150 m.a.s.l.1. III.1998 / G. Ortega, E. Barrera” (1) | CNIN.

### Cenophengus mboi

Taxon classificationAnimaliaColeopteraPhengodidae

Vega-Badillo et al. 2021

6F525195-DE15-5B54-86FA-2AD25BA87C86

Fig. 18A–H

Cenophengus mboi Vega-Badillo et al. 2021a: 227.

#### Type locality.

Hidalgo, Mexico (Fig. 19).

#### Type material examined.

***Holotype*** ♂: “Mexico: Santiago de Anaya/ Hgo.20°24'0761"N/ 98°53'1797"O, 28-29 agosto /2017 Col. A. Ibarra Vázquez” |CNIN. ***Paratype*** ♂: “Mexico, Atotonilco El / Grande, 3 km NE Montecillos/ Bosque Juniperus-Quercus. 20° /18' 9" N, 98° 36'17" W. Trampa de / Intercepción de vuelo 12 al 19-VII-/2010. J. Márquez y J. Asiain” | CC-UAEH.

#### Remarks.

*Cenophengusmboi* is morphologically similar to *C.predregalensis*, but can be distinguished by the colour of the body and terminal maxillary palpomere. In *C.mboi*, the body is dark brown, whereas in *C.pregalensis*, it is dark brown and the pronotum yellow-orange. Terminal maxillary palpomere is as long as the preceding three combined in *C.mboi*, in *C.pedregalensis*, it is longer than the preceding three combined.

#### Diagnosis.

Body black, integument chagreened, antennae long, more than twice the length of pronotum, antennal rami twice as long as the respective antennomere, terminal maxillary palpomere is as long as the preceding three combined and each elytron 4.3 times as long as wide; aedeagus with three teeth at the inner apex of paramere.

#### Redescription.

**Figure 18.**
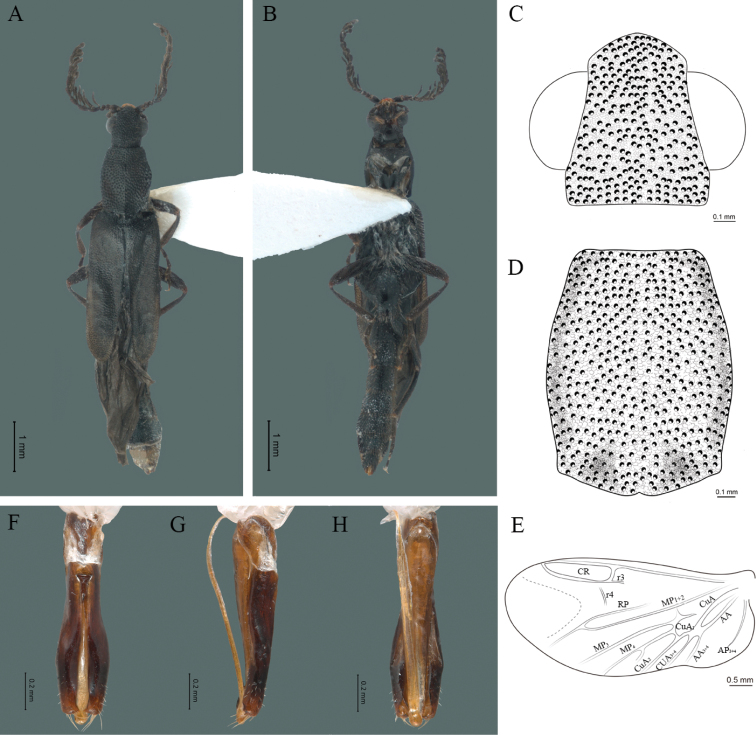
*Cenophengusmboi* Vega-Badillo et al. 2021, male. Habitus: **A** dorsal **B** ventral **C** head dorsal **D** pronotum dorsal **E** hind wing. Wing venation: CR = Radial Cell; r3 = radial 3 vein; r4 = radial 4 vein; RP = Posterior Radial vein; MP1+2 = Posterior Median vein; CuA = Cubital vein; AA and AP = Anterior and Posterior Anal veins. Aedeagus: **F** dorsal view **G** lateral view **H** ventral view.

**Male.** Body length 8.0–9.6 mm; maximum body width 0.8–1.0 mm (pronotum). Body black (Fig. 18A, B). ***Head*.** As wide (0.76–0.80 mm) (0.78 ± 0.028 mm, n = 2) as long (0.70–0.81 mm) (0.76 ± 0.084 mm, n = 2) (Fig. 18C), almost as wide as pronotum, integument chagreened, punctures as long as eye facets and separated by approximately 1 punctured diameter, each puncture bearing a yellow-brown seta; interantennal distance (0.10 mm) (0.10 ± 0 mm, n = 2) less than the length of antennomere 1 (0.20–0.25 mm) (0.225 ± 0.035 mm, n = 2); eyes 1/2 as long as head in lateral view, longer (0.27–0.32 mm) (0.295 ± 0.035 mm, n = 2) than wide (0.14–0.15 mm) (0.145 ± 0.007 mm, n = 2); interocular distance (0.43–0.48 mm) (0.455 ± 0.035 mm, n = 2) 3.5 times eye width; antennae long (2.37–2.66 mm) (2.43 ± 0.205 nn, n = 2) more than twice the length of pronotum; antennomere 1 (0.20–0.25 mm) (0.225 ± 0.035 mm, n = 2) longer than the next two combined, antennomere 3 cup-shaped, 4 (0.21–0.23 mm) (0.22 ± 0.014 mm, n = 2) shorter than the following antennomeres, 5 to 11 about equal in length (0.22–0.25 mm) (0.26 ± 0.014 mm, n = 2), 12 (terminal) (0.25–0.27 mm) (0.26 ± 0.014 mm, n = 2), antennal rami lanceolate in lateral view, twice as long as the respective antennomere; terminal maxillary palpomere robust, securiform (0.30–0.35 mm) (0.325 ± 0.035 mm, n = 2), as long as the preceding three combined; terminal labial palpomere spindle-shaped (0.13–0.15 mm) (0.14 ± 0.014 mm, n = 2), 3 times as long as preceding one (0.04–0.05 mm) (0.045 ± 0.007 mm, n = 2). ***Thorax*.** Pronotum longer (1.02–1.30 mm) (1.16 ± 0.197 mm, n = 2) than wide (0.8–1.0 mm) (0.9 ± 0.141 mm, n = 2) (Fig. 18D); integument chagreened, punctures as long as eye facets and separated by approximately 1 punctured diameter, each puncture bearing a yellow-brown seta, disc convex, weakly elevated dorsally forming a small depression in the basal part of each side, posterior margin almost straight with middle notch, sides almost straight, anterior and posterior angles rounded; mesosternal suture complete; scutellum with posterior margin rounded; each elytron 4.3 times as long (2.48–2.60 mm) (2.54 ± 0.084 mm, n = 2) as wide (0.60–0.68 mm) (0.64 ± 0.056 mm, n = 2), convex, without longitudinal costae, elytral apex rounded; hind wings with posterior radial vein (RP) length twice less than the length of MP1+2, radial cell closed, r3 vein present, r4 vein reduced (not reaching the RP or the radial cell), those of the anterior anal and posterior anal sectors, evident (Fig. 18E). Legs: tarsomeres 1 and 2 of prothoracic legs about equal in length, tarsomere 1 of meso- and metathoracic legs longer than 2. ***Abdomen*.** Integument shiny, punctured, with long dense setae, sternite 7 with margin sinuate, sternite 8 with margin notched; aedeagus with three teeth at the inner apex of paramere (Fig. 18F–H).

#### Female and immatures.

Unknown.

#### Distribution.

**Figure 19.**
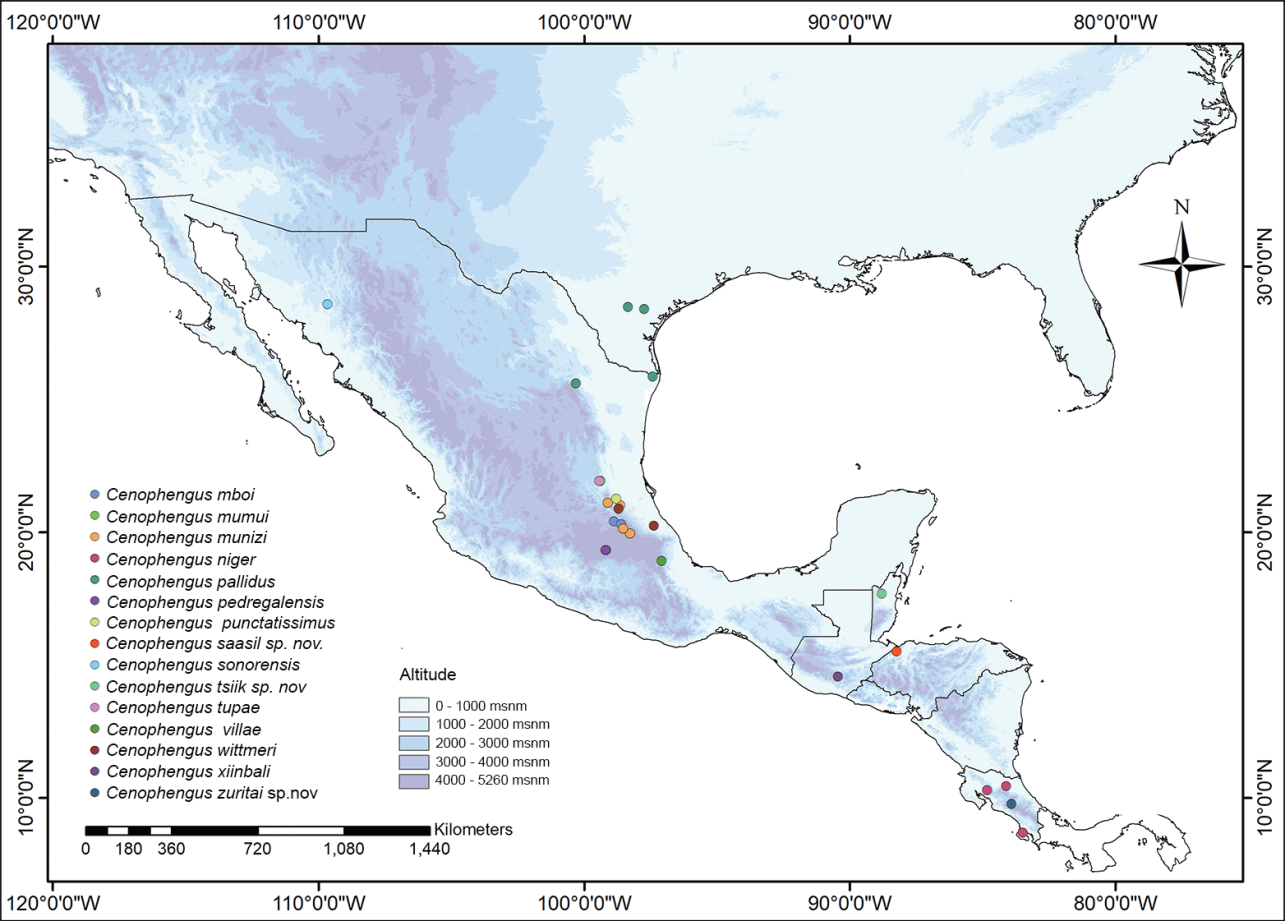
Map of southern North America showing specimen localities for *Cenophengus* spp. (continued).

Mexico: Hidalgo (Fig. 19).

### Cenophengus mumui

Taxon classificationAnimaliaColeopteraPhengodidae

Vega-Badillo et al. 2021

A6B6241D-8DC0-5F64-881A-707A8CC00479

Fig. 20A–H

Cenophengus mumui Vega-Badillo et al. 2021a: 231.

#### Type locality.

San Luis Postosí, Mexico.

#### Type material examined.

***Holotype*** ♂: “Mexico, San Luis Potosí, / Tamasopo. Cerro al noroeste/ del cafetal, 01-06-15, / N 21°55.47' W 99°24.95' Col. / Jessica Ríos” |CNIN.

#### Remarks.

*Cenophengusmumui* is in a clade with *C.munizi* and *C.huatulcoensis* (Vega-Badillo et al. 2021b), but can be distinguished from *C.munizi* by the shape and colour of the head. In *C.mumui*, the head is square and brown, whereas in *C.munizi*, it is rectangular-shaped. Additionally, in *C.mumui*, the antennal rami are 1.5 times as long as the respective antennomere, whereas in *C.munizi*, they are twice as long as the respective antennomere. Finally, *C.mumui* can be distinguished from *C.huatulcoensis* by the interantennal distance and interocular distance. In *C.huatulcoensis*, the interantennal distance is equal to the length of antennomere 1, whereas in *C.mumui*, it is less than the length of antennomere 1. The interocular distance is 2 times eye width in *C.mumui*, in *C.huatulcoensis*, it is 3 times eye width.

#### Diagnosis.

Body yellow, except for head brown, integument smooth, antennae long more than twice the length of pronotum, antennal rami 1.5 times the respective antennomere, pronotum as long as wide and each elytron 4.3 times as long as wide; aedeagus with one spine at the inner apex of paramere.

#### Redescription.

**Figure 20.**
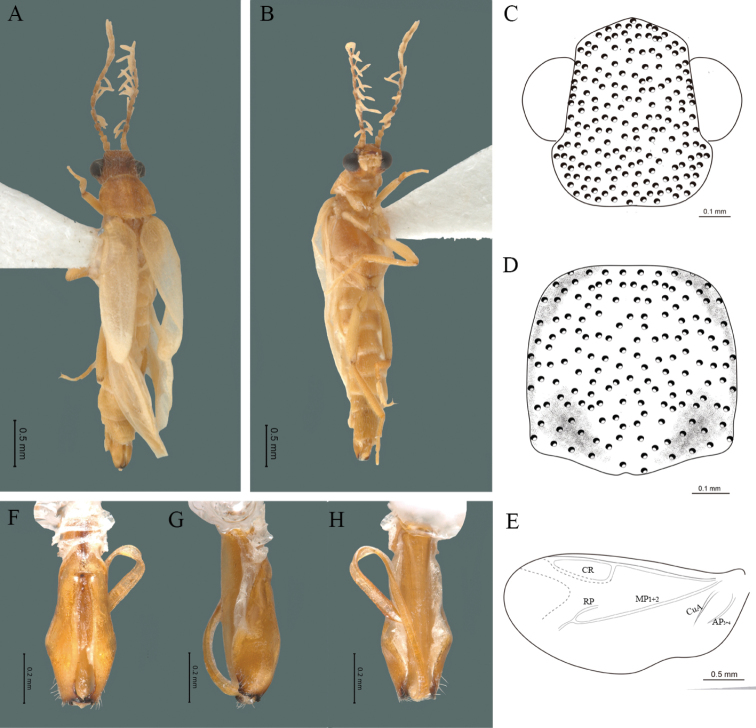
*Cenophengusmumui* Vega-Badillo et al. 2021, male. Habitus: **A** dorsal **B** ventral **C** head dorsal **D** pronotum dorsal **E** hind wing. Wing venation: CR = Radial Cell; RP = Posterior Radial vein; MP1+2 = Posterior Median vein; CuA = Cubital vein; AP = Posterior Anal vein. Aedeagus: **F** dorsal view **G** lateral view **H** ventral view.

**Male.** Body length 3.5–4.0 mm; maximum body width 0.56–0.59 mm (pronotum). Body yellow, except for head brown (Fig. 20A, B). ***Head*.** Wider (0.58–0.61 mm) (0.595 ± 0.458 mm, n = 2) than long (0.49–0.52 mm) (0.505 ± 0.021 mm, n = 2) (Fig. 20C), at eye level, almost as wide as the pronotum, integument smooth, punctures twice as long as eye facets and separated by approximately 1 punctured diameter, each puncture bearing a yellow seta; interantennal distance (0.09–0.10 mm) (0.095 ± 0.007 mm, n = 2) less than the length of antennomere 1 (0.13–0.15 mm) (0.14 ± 0.014 mm, n = 2); eyes 1/2 as long as head in lateral view, longer (0.20–0.22 mm) (0.21 ± 0.014 mm, n = 2) than wide (0.13–0.15 mm) (0.14 ± 0.028 mm, n = 2); interocular distance (0.32–0.36 mm) (0.34 ± 0.028 mm, n = 2) twice as long as eye width; antennae long (1.67–1.87 mm) (1.77 ± 0.141 mm, n = 2), more than twice the length of pronotum; antennomere 1 (0.13–0.15 mm) (0.14 ± 0.014 mm, n = 2) as long as the next two combined, antennomere 3 cup-shaped, 4 (0.12–0.13 mm) (0.125 ± 0.007 mm, n = 2) shorter than the following antennomeres; 5 to 11 about equal in length (0.15–0.17 mm) (0.16 ± 0.014 mm, n = 2), 12 (terminal) (0.25–0.27 mm) (0.26 ± 0.014 mm, n = 2), antennal rami lanceolate in lateral view, 1.5 times as long as respective antennomere; terminal maxillary palpomere robust, securiform (0.15–0.16 mm) (0.155 ± 0.007 mm, n = 2), as long as the preceding three combined; terminal labial palpomere spindle-shaped (0.05–0.06 mm) (0.055 ± 0.007 mm, n = 2), twice as long as the preceding one (0.02–0.03 mm) (0.025 ± 0.007 mm, n = 2). ***Thorax*.** Pronotum as long (0.58–0.62 mm) (0.6 ± 0.028 mm, n = 2) as wide (0.56–0.59 mm) (0.575 ± 0.021 mm, n = 2) (Fig. 20D); integument smooth, punctures twice as long as eye facets and separated by approximately 2 punctured diameters, with a yellow-coloured seta in each puncture; disc convex, weakly elevated dorsally forming a small depression in the basal part of each side, posterior margin almost straight with middle notch, sides convex, anterior and posterior angles rounded; mesosternal suture incomplete; scutellum with posterior margin rounded; each elytron 4.3 times as long (1.44–1.62 mm) (1.53 ± 0.127 mm, n = 2) as wide (0.34–0.37 mm) (0.355 ± 0.021 mm, n = 2), convex, without longitudinal costae, elytral apex right angled; posterior radial vein (RP) length 4.8 times less than the length of MP1+2, radial cell closed and slightly defined, r3 and r4 veins absent, those of the anterior anal and posterior anal sectors, absent (Fig. 20E). Legs: tarsomeres 1 of pro-, meso- and metathoracic legs with a similar length. ***Abdomen*.** Integument shiny, punctured, with long dense setae, sternite 7 with margin sinuate, sternite 8 with margin notched; aedeagus with one spine at the inner apex of paramere (Fig. 20F–H).

#### Immatures and females.

Unknown.

#### Distribution.

Mexico: San Luis Postosí and Tamaulipas (Fig. 19).

#### Additional material examined.

“MEXICO: Tamps. Mun. / Gómez Farías, Al/ Cimas, 1000 m. 22- / III-1987 P. Kovarik/ R. Jones; UV light” “From the Michael / Ivie Collection” (1) |CNIN.

### Cenophengus munizi

Taxon classificationAnimaliaColeopteraPhengodidae

Zaragoza-Caballero, 2008

8E5FEE59-5A01-5102-AA0A-89DA552C1AB1

Fig. 21A–H

Cenophengus munizi Zaragoza-Caballero, 2008: 155.

#### Type locality.

Hidalgo, Mexico.

#### Type material examined.

***Holotype*** ♂: “MEXICO: Hidalgo, Tlanchinol, La/ Cabaña. Bosque Mesófilo de montaña/ 1478 m. N 21° 01.3343', W 98° 38.600' / Trampa de intercepción de vuelo 1. /13-20 -mayo- 2006. C. Ortiz y M.C. / Pedraza.”

#### Remarks.

*Cenophengusmunizi* is in a clade with *C.mumui* and *C.huatulcoensis* (Vega-Badillo et al. 2021b), but can be distinguished from *C.mumui* by the shape and colour of the head and interantennal distance. *Cenophengusmunizi* exhibits a rectangular-shaped head, which is yellow-brown coloured like the rest of the body, whereas in *C.mumui*, it is square and brown. In *C.munizi*, the interantennal distance is equal to the length of antennomere 1, whereas in *C.mumui*, it is less than the length of antennomere 1. Additionally, in *C.munizi*, the antennal rami are twice as long as the respective antennomere, whereas in *C.mumui*, they are 1.5 times as long as the respective antennomere. Finally, *C.munizi* can be distinguished from *C.huatulcoensis* by the interocular distance. The interocular distance is 2 times eye width in *C.mumui*, in *C.huatulcoensis*, it is 3 times eye width.

#### Diagnosis.

Integument smooth, head almost as wide as the pronotum, antennae long, more than twice the length of pronotum, antennal rami twice as long as respective antennomere, each elytron 6 times as long as wide; aedeagus one with spine at the inner apex of paramere.

#### Redescription.

**Figure 21.**
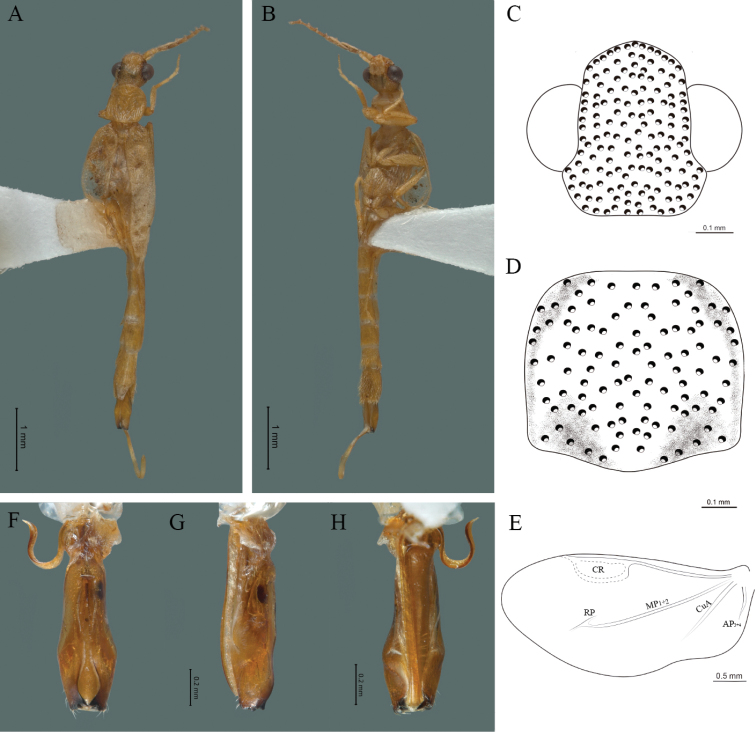
*Cenophengusmunizi* Zaragoza-Caballero, 2008, male. Habitus: **A** dorsal **B** ventral **C** head dorsal **D** pronotum dorsal **E** hind wing. Wing venation: CR = Radial Cell; RP = Posterior Radial vein; MP1+2 = Posterior Median vein; CuA = Cubital vein; AP = Posterior Anal vein. Aedeagus: **F** dorsal view **G** lateral view **H** ventral view.

**Male.** Body length 4.8–6.2 mm; maximum body width 0.58–0.65 mm (pronotum). Body yellow, elytra yellow with whitish apical part (Fig. 21A, B). ***Head*.** Wider (0.63–0.65 mm) (0.637 ± 0.009 mm, n = 4) than long (0.42–0.45 mm) (0.435 ± 0.017 mm, n = 4) (Fig. 21C), at eye level, almost as wide as the pronotum, integument smooth, punctures twice as long as eye facets and separated by approximately 0.5 punctured diameters, each puncture bearing a yellow-brown seta; interantennal distance (0.11–0.12 mm) (0.115 ± 0.005 mm, n = 4) equal to the length of antennomere 1 (0.12–0.15 mm) (0.13 ± 0.017 mm, n = 4); eyes 1/2 as long as head in lateral view, longer (0.20–0.22 mm) (0.21 ± 0.011 mm, n = 4) than wide (0.14–0.15 mm) (0.145 ± 0.005 mm, n = 4); interocular distance (0.33–0.36 mm) (0.345 ± 0.017 mm, n = 4) twice as long as eye width; antennae long (2.13–2.21 mm) (2.15 ± 0.039 mm, n = 4), more than twice the length of pronotum; antennomere 1 (0.12–0.15 mm) (0.13 ± 0.017 mm, n = 4) as long as the next two combined, antennomere 3 cup-shaped, the 4 (0.15–0.17 mm) (0.157 ± 0.009 mm, n = 4) shorter than the following antennomeres; 5 to 11 about equal in length (0.22–0.30 mm) (0.30 ± 0.458 mm, n = 4), 12 (terminal) (0.30) (0.30 ± 0 mm, n = 4), antennal rami lanceolate in lateral view, twice as long as the respective antennomere; terminal maxillary palpomere robust, securiform (0.19–0.20 mm) (0.195 ± 0.005 mm, n = 4), shorter than the preceding three combined; terminal labial palpomere spindle-shaped (0.05–0.06 mm) (0.055 mm ± 0.005 mm, n = 4), 5 times as long as preceding one (0.01 mm) (0.01 ± 0 mm, n = 4). ***Thorax*.** Pronotum as long (0.62–0.70 mm) (0.65 ± 0.052 mm, n = 4) as wide (0.58–0.65 mm) (0.62 ± 0.035 mm, n = 4) (Fig. 21D); integument smooth, punctures twice as long as eye facets and separated by approximately 2 punctured diameters, with a yellow-brown seta in each puncture; disc convex, weakly elevated dorsally forming a small depression in the basal part of each side, posterior margin curved with middle notch, sides convex, anterior and posterior angles rounded; mesosternal suture incomplete; scutellum with posterior margin rounded; each elytron 6 times as long (1.80–2.16 mm) (1.98 ± 0.207 mm, n = 4) as wide (0.36–0.40 mm) (0.38 ± 0.023 mm, n = 4), convex, without longitudinal costae, elytral apex right angled; hind wings with posterior radial vein (RP) length 10 times less than the length of MP1+2, radial cell closed and slightly defined, r3 and r4 veins absent, those of the anterior anal and posterior anal sectors, absent (Fig. 21E). Legs: tarsomeres 1 and 2 of pro-, meso- and metathoracic legs with a similar length. ***Abdomen*.** Integument shiny, punctured, with long dense setae, sternite 7 with margin sinuate, sternite 8 with margin notched; aedeagus one with spine at the inner apex of paramere (Fig. 21F–H).

#### Immatures and females.

Unknown.

#### Distribution.

Mexico: Hidalgo (Fig. 19).

#### Additional material examined.

“MEXICO: Hidalgo, Cuautepec /Tezoncualpan “El Caminero” / Bosque de encino. / 19° 56' 53.8" N; 98° 16' 27.9" W. /Trampa intercepción de vuelo, / 22 a 29- VIII-2009, M. Torres col.” (1) | CNIN; “MEXICO, Hidalgo, La Misión, Lomas / del Pericón, Bosque Mesofilo de mon- /taña perturbado. 1377 m. N 21° 06' 46.0"/ W 99° 06' 15.6". Trampa de intercepción / de vuelo. Del 8 al 16-III-2008/ J. Márquez y J. Asiain cols.” (1) | CNIN; “MEXICO, Hidalgo, La Misión, Lomas/ del Pericón, Bosque Mesofilo de mon- /taña perturbado. 1377 m. N 21° 06' 46.0" /W 99° 06' 15.6". NTP-80 (Calamar). Del 8 / al 16-III-2008. J. Márquez y J. Asiain cols.” (1) | CNIN. “MEXICO: Hidalgo, Tlanchinol/ J. Márquez y J. Asiain cols.” (2) | CC-UAEH.

### Cenophengus niger

Taxon classificationAnimaliaColeopteraPhengodidae

Wittmer, 1986

16CFB7AD-0190-54B7-9F70-206AB5941961

Fig. 22A–H

Cenophengus niger Wittmer, 1986: 160.

#### Type locality.

Monteverde, Costa Rica.

#### Type material examined.

***Holotype*** ♂: “COSTA RICA: Punt. / Monteverde. 1400 m/ 23 May 1979/ H & A Howden” “*Cenophengus/ niger* Wittmer” “PHENGODIDAE/ PHENG00000347” | NHMB.

#### Remarks.

*Cenophengusniger* is morphologically similar to *C.howdeni*, but can be distinguished by the length of antennomere 1 and the diameters of the punctures. In *C.niger*, antennomere 1 is equal to the length of antennomeres 2 and 3 combined, whereas in *C.howdeni*, it is shorter than antennomeres 2 and 3 combined. In *C.niger*, the punctures are twice as long as eye facets and separated by approximately 0.2 punctured diameters, whereas in *C.howdeni*, they are as long as eye facets and separated by approximately 1 punctured diameter.

#### Diagnosis.

Integument chagreened, head less wide than pronotum, antennae less than twice the length of the pronotum, antennal rami twice as long as the respective antennomere and each elytron 3.5 times as long as wide; aedeagus with three teeth at the inner apex of paramere.

#### Redescription.

**Figure 22.**
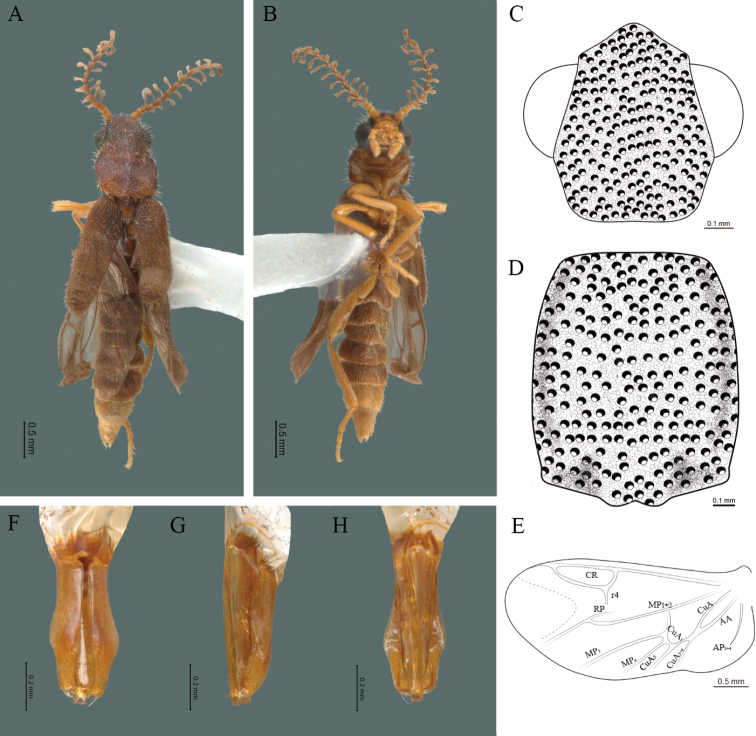
*Cenophengusniger* Wittmer, 1986, male. Habitus: **A** dorsal **B** ventral **C** head dorsal **D** pronotum dorsal **E** hind wing. Wing venation: CR = Radial Cell; r4 = radial 4 vein; RP = Posterior Radial vein; MP1+2 = Posterior Median vein; CuA = Cubital vein; AA and AP = Anterior and Posterior Anal veins. Aedeagus: **F** dorsal view **G** lateral view **H** ventral view.

**Male.** Body length 6.0–6.3 mm, maximum body width 0.80–0.85 mm (pronotum). Body dark brown, only mouthparts, three first antennomeres, two last abdominal segments, all legs with trochanter and coxae yellowish (Fig. 22A, B). ***Head*.** Wider (0.80–0.83 mm) (0.81 ± 0.0.17 mm, n = 3) than long (0.55–0.60 mm) (0.56 ± 0.028 mm, n = 3) (Fig. 22C), at eye level, less wide than the pronotum, integument chagreened, punctures twice as long as eye facets and separated by approximately 0.2 punctured diameters, each puncture bearing a yellow-brown seta; interantennal distance (0.08–0.10 mm) (0.089 ± 0.11 mm, n = 3) less than the length of antennomere 1 (0.14–0.15 mm) (0.146 ± 0.005 mm, n = 3); eyes 1/2 as long as head in lateral view , longer (0.26–0.28 mm) (0.266 ± 0.011 mm, n = 3) than wide (0.12–0.14 mm) (0.126 ± 0.011 mm, n = 3); interocular distance (0.40–0.42 mm) (0.403 ± 0.011 mm, n = 3) 3 times eye width; antennae short (2.2–2.3 mm) (2.26 ± 0.057 mm, n = 3) less than twice the length of the pronotum; antennomere 1 (0.14–0.15 mm) (0.146 ± 0.005 mm, n = 3) equal to the length of the next two combined, antennomere 3 cup-shaped, 4 (0.13–0.14 mm) (0.136 ± 0.005 mm, n = 3) shorter than following antennomeres, 5 to 11 about equal in length (0.15–0.16) (0.156 ± 0.005, n = 3), 12 (terminal) (0.20–0.21 mm) (0.206 ± 0.005 mm, n = 3), antennal rami lanceolate in lateral view, twice as long as the respective antennomere; terminal maxillary palpomere robust, securiform (0.25–0.27 mm) (0.263 ± 0.011 mm, n = 3), as long as the preceding three combined; terminal labial palpomere spindle-shaped (0.05–0.06 mm) (0.056 ± 0.005 mm, n = 3), 3 times as long as preceding one (0.02 mm) (0.02 ± 0 mm, n = 3). ***Thorax*.** Pronotum longer (0.95–0.98 mm) (0.966 ± 0.015 mm, n = 3) than wide (0.80–0.85 mm) (0.833 ± 0.0.11 mm, n = 3) (Fig. 22D); integument chagreened, punctures twice as long as eye facets and separated by approximately 0.2 punctured diameters, with a yellow-brown seta in each puncture; disc convex, weakly elevated dorsally forming a small depression in the basal part of each side, posterior margin curved, sides almost straight, anterior angles rounded and posterior angles acute; mesosternal suture complete; scutellum with posterior margin rounded; each elytron 3.5 times as long (1.62–1.74 mm) (1.68 ± 0.061 mm, n = 3) as wide (0.46–0.48 mm) (0.473 ± 0.011 mm, n = 3), convex, without longitudinal costae, elytral apex rounded; hind wings with posterior radial vein (RP) length 6.5 times less than the length of MP1+2, radial cell closed, r3 vein absent, r4 vein developed (reaching the radial cell), those of the anterior anal and posterior anal sectors, evident (Fig. 22E). Legs: tarsomere 1 of pro-, meso- and metathoracic legs is longer than 2. ***Abdomen*.** Integument shiny, punctured, with long dense setae, sternite 7 with margin sinuate, sternite 8 with margin notched; aedeagus with three teeth at the inner apex of paramere (Fig. 22F–H).

#### Female and immatures.

Unknown.

#### Distribution.

Costa Rica: Monteverde, Heredia and Puntarenas (Fig. 19).

#### Additional material examined.

“COSTA RICA: Heredia/ La Selva, 75 m / 10°26'N, 84°01'W /Sept 1992 / P. Hansen, Malaise” “From the Michael/ Ivie Collection” (2) | MTEC; “COSTA RICA: Puntarenas/ 3 km SW Rincón /8.683°N, 83.483°W July 1991. 10 m /P. Hanson. Malaise” “From the Michael/ Ivie Collection” (1) | MTEC.

### Cenophengus pallidus

Taxon classificationAnimaliaColeopteraPhengodidae

Schaeffer, 1904

158262C7-1779-57D0-A3D5-2BF8CEFFD4DA

Fig. 23A–H

Cenophengus pallidus Schaeffer, 1904: 213.

#### Type locality.

Texas, USA.

#### Type material.

***Holotype*** ♂: USA “Texas. Brownsvell, 21.V. 1904, H.S. Barber col.” | BMNH.

#### Remarks.

*Cenophenguspallidus* is sister to *C.sonoraensis* (Vega-Badillo et al. 2021b), but can be distinguished by the colour of the body and the interocular distance. In *C.pallidus*, body is yellow, whereas in *C.sonoraensis*, it is pale brown. The interocular distance is 1.5 times eye width in *C.pallidus*, in *C.sonoraensis*, it is twice eye width. Additionally, in *C.pallidus*, the pronotal disc has a longitudinal carina, whereas in *C.sonoraensis*, it is weakly elevated dorsally forming a small depression in the basal part of each side.

#### Diagnosis.

Integument chagreened, head wider than the pronotum, antennae short, less than twice the length of the pronotum, antennal rami twice as long as the respective antennomere and each elytron 3.5 times as long as wide; aedeagus with three teeth at the inner apex of paramere.

#### Redescription.

**Figure 23.**
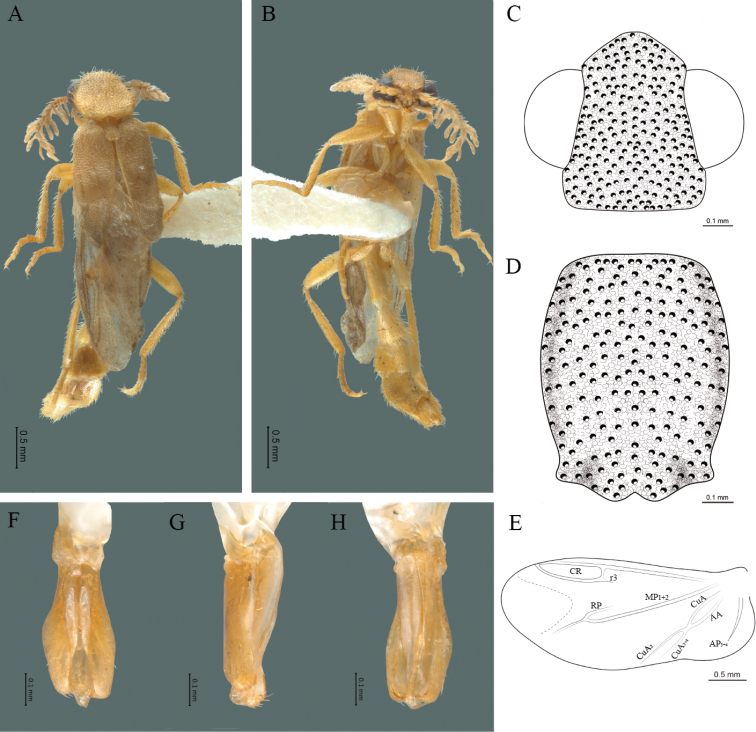
*Cenophenguspallidus* Schaeffer, 1904, male. Habitus: **A** dorsal **B** ventral **C** head dorsal **D** pronotum dorsal **E** hind wing. Wing venation: CR = Radial Cell; r3 = radial 3 vein; RP = Posterior Radial vein; MP1+2 = Posterior Median vein; CuA = Cubital vein; AP = Posterior Anal vein. Aedeagus: **F** dorsal view **G** lateral view **H** ventral view.

**Male.** Body length 3.84–4.50 mm, maximum body width 0.53–0.76 mm (pronotum). Body yellow (Fig. 23A, B). ***Head*.**Wider (0.61–0.73) (0.65 ± 0.059, n = 6) than long (0.34–0.42 mm) (0.386 ± 0.037 mm, n = 6) (Fig. 23C), at eye level, wider than the pronotum, integument chagreened, punctures 1.5 times as long as eye facets and separated by approximately 1 punctured diameter, each puncture bearing a yellow seta; interantennal distance (0.06–0.08 mm) (0.075 ± 0.0083 mm, n = 6) less than the length of antennomere 1 (0.14–0.16 mm) (0.15 ± 0.006 mm, n = 6); eyes 3/4 as long as head in lateral view, longer (0.30–0.32 mm) (0.31 ± 0.008 mm, n = 6) than wide (0.16–0.19 mm) (0.173 ± 0.013 mm, n = 6); interocular distance (0.27–0.35 mm) (0.30 ± 0.036 mm, n = 6) 1.5 times eye width; antennae short (1.20–1.34 mm) (1.275 ± 0.056 mm, n = 6) less than twice the length of the pronotum; antennomere 1 (0.14–0.16 mm) (0.15 ± 0.006 mm, n = 6) is longer than the next two combined, antennomere 3 cup-shaped, 4 (0.08–0.12 mm) (0.095 ± 0.015 mm, n = 6) shorter than the following antennomeres, 5 to 11 about equal in length (0.10–0.12 mm) (0.11 ± 0.008 mm, n = 6), 12 (terminal) (0.16–0.18 mm) (0.166 ± 0.01 mm, n = 6), antennal rami lanceolate in lateral view, twice as long as the respective antennomere; terminal maxillary palpomere robust, securiform (0.21–0.25 mm) (0.23 ± 0.017 mm, n = 6), as long as the preceding three combined; terminal labial palpomere spindle-shaped (0.09–0.10 mm) (0.095 ± 0.005 mm, n = 6), 3 times as long as preceding one (0.02–0.03) (0.21 ± 0.004, n = 6). ***Thorax*.** Pronotum longer (0.69–1.0 mm) (0.83 ± 0.14 mm, n = 6) than wide (0.53–0.76 mm) (0.64 ± 0.102 mm, n = 6) (Fig. 23D); integument chagreened, punctures 1.5 times as long as eye facets and separated by approximately 2 punctured diameters, each puncture bearing a yellow seta, disc convex, weakly elevated dorsally forming a small depression in the basal part of each side, posterior margin curved, sides almost straight, anterior angles rounded and posterior angles acute; mesosternal suture incomplete; scutellum with posterior margin rounded; each elytron 3.5 times as long (1.2–1.4 mm) (1.33 ± 0.103 mm, n = 6) as wide (0.30–0.44 mm) (0.38 ± 0.064 mm, n = 6), convex, without longitudinal costae, elytral apex rounded; hind wings with posterior radial vein (RP) length 5.5 times less than the length of MP1+2, radial cell closed, r3 vein absent, r4 vein absent, those of the anterior anal and posterior anal sectors, evident (Fig. 23E). Legs: tarsomere 1 of pro-, meso- and metathoracic legs is longer than 2. ***Abdomen*.** Integument shiny, punctured, with long dense setae, sternite 7 with margin sinuate, sternite 8 with margin notched; aedeagus with three teeth at the inner apex of paramere (Fig. 23F–H).

#### Female and immatures.

Unknown.

#### Distribution.

USA: Texas; Mexico: Nuevo León (Fig. 19).

#### Additional material examined.

USA: “Texas, Cameron Co. / Sabal Palm Grove/ Audobon Reserve/ 26-28 May 1979/ N. M. Downie” (1) |FMNH. “Texas, Bee Co. / 5 m N. Beeville/ on US181/1 June 1979/ N. M. Downie” “*Cenophengus/ pallidus* /Schaeffer” “N.M. Downie Colln. / 1992 Acc. Z-18,343/ FIELD MUSEUM” (1) |FMNH; “Esprza Rch/ Brownsville, Tex.” (1) | CNIN; “Mexico: 5 mi. S. Monterrey/ N.L. Mex. VII.22.1963/ H. Howden” “*Cenophengus/ pallidus* Schaeffer/ det. W. Wittemer” (1) |FMNH. “Tx. Cameron Co. / Sabal Palm Grove/ June 9-10-1978/ J.E. Wappes” “*C.pallidus*” (2) | FSCA.

### Cenophengus pedregalensis

Taxon classificationAnimaliaColeopteraPhengodidae

Zaragoza-Caballero, 1975

5B62F4FA-13D1-518D-9BE6-D3F5906B547F

Fig. 24A–H

Cenophengus pedregalensis Zaragoza-Caballero, 1975: 452.

#### Type locality.

Mexico City, Mexico.

#### Type material examined.

***Holotype*** ♂: Mexico: “Pedregal San Ángel/ 11-VIII-69/ S. Zaragoza” | CNIN. ***Paratype*** ♂: Mexico: “Jardín Botánico, C.U. / D.F. 2. VIII.69. /S. Zaragoza-Caballero” (6) | CNIN.

#### Remarks.

*Cenophenguspedregalensis* is morphologically similar to *C.mboi*, but can be distinguished by the colour of the body and the terminal maxillary palpomere. In *C.predregalensis*, body is dark brown and pronotum yellow-orange, whereas in *C.mboi*, it is dark. The terminal maxillary palpomere is longer than the preceding three combined in *C.pedregalensis*, in *C.mboi*, it is as long as the preceding three combined.

#### Diagnosis.

Body dark brown with pronotum yellow-orange, integument chagreened, antennae long, more than twice the length of pronotum, antennal rami twice as long as the respective antennomere, terminal maxillary palpomere is longer than the preceding three combined and each elytron 4.5 times as long as wide; aedeagus with three teeth at the inner apex of paramere.

#### Redescription.

**Figure 24.**
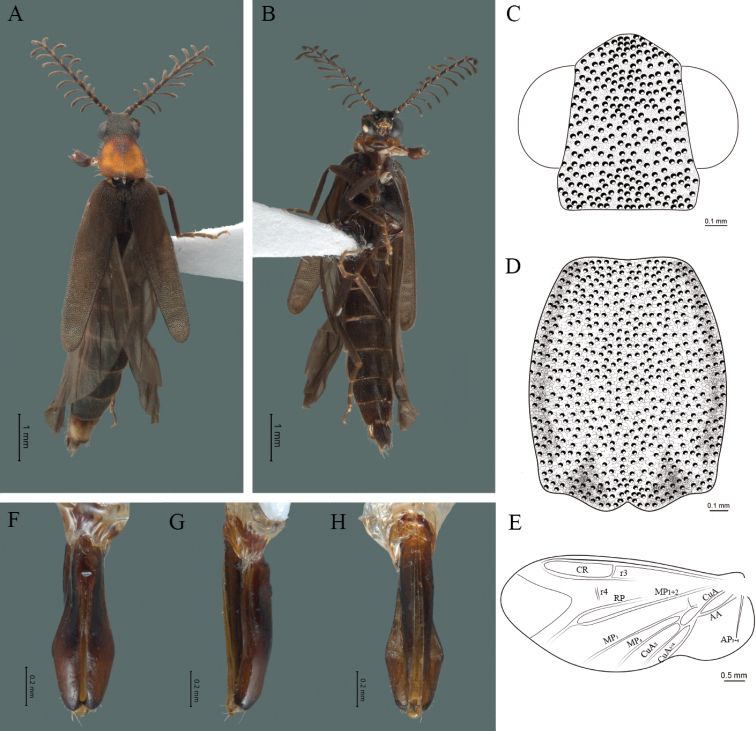
*Cenophenguspedregalensis* Zaragoza-Caballero, 1975, male. Habitus: **A** dorsal **B** ventral **C** head dorsal **D** pronotum dorsal **E** hind wing. Wing venation: CR = Radial Cell; r3 = radial 3 vein; r4 = radial 4 vein; RP = Posterior Radial vein; MP1+2 = Posterior Median vein; CuA = Cubital vein; AA and AP = Anterior and Posterior Anal veins. Aedeagus: **F** dorsal view **G** lateral view **H** ventral view.

**Male.** Body length 7.2–10.5 mm, maximum body width 1.01–1.10 mm (pronotum). Body dark brown, antennae black to brown, pronotum yellow-orange (Fig. 24A, B). ***Head*.** Wider (0.86–0.96 mm) (0.91 ± 0.061 mm, n = 11) than long (0.60–0.70 mm) (0.63 ± 0.041 mm, n = 11) (Fig. 24C), at eye level, less wide than the pronotum, integument chagreened, punctures 1.5 times as long as eye facets and separated by approximately 1 punctured diameter, each puncture bearing a yellow-brown seta; interantennal distance (0.10–0.11 mm) (0.10 ± 0.005 mm, n = 11) less than the length of antennomere 1 (0.22–0.25 mm) (0.24 ± 0.009 mm, n = 11); eyes 1/3 as long as head in lateral view , longer (0.35–0.40 mm) (0.38 ± 0.018 mm, n = 11) than wide (0.20–25 mm) (0.22 ± 0.022 mm, n = 11); interocular distance (0.45–0.55 mm) (0.48 ± 0.04 mm, n = 11) 2.3 times longer than eye width; antennae long (2.68–2.81 mm) (2.72 ± 0.044 mm, n = 11), more than twice the length of pronotum; antennomere 1 (0.22–0.25 mm) (0.24 ± 0.009 mm, n = 11) as long as the next two combined, antennomere 3 cup-shaped, 4 (0.20–0.23 mm) (0.21 ± 0.011 mm, n = 11) shorter than the following antennomeres, 5 to 11 about equal in length (0.25–0.26 mm) (0.253 ± 0.004 mm, n = 11), 12 (terminal) (0.30–0.35 mm) (0.32 ± 0.026 mm, n = 11), antennal rami lanceolate in lateral view, twice as long as the respective antennomere; terminal maxillary palpomere robust, securiform (0.28–0.36 mm) (0.32 ± 0.031 mm, n = 11), longer than the preceding three combined; terminal labial palpomere spindle-shaped (0.14–0.15 mm) (0.147 ± 0.004 mm, n = 11), 4 times as long as preceding one (0.04–0.05 mm) (0.44 ± 0.005 mm, n = 11). ***Thorax*.** Pronotum longer (1.15–1.40 mm) (1.24 mm ± 0.087 mm, n = 11) than wide (1.01–1.10 mm) (1.07 ± 0.034 mm, n = 11) (Fig. 24D); integument chagreened, punctures twice as large as eye facets and separated by approximately 1 punctured diameter, each puncture bearing a yellow-brown seta, disc convex, weakly elevated dorsally forming a small depression in the basal part of each side, posterior margin curved with middle notch, sides almost straight, anterior angles rounded and posterior angles acute; mesosternal suture complete; scutellum with posterior margin rounded; each elytron 4.5 times as long (3.0–3.52 mm) (3.33 ± 0.17 mm, n = 11) as wide (0.65–0.80 mm) (0.70 ± 0.056 mm, n = 11), convex, without longitudinal costae, elytral apex rounded; hind wings with posterior radial vein (RP) length twice less than the length of MP1+2, radial cell closed, r3 vein present, r4 vein reduced (not reaching the RP or the radial cell), those of the anterior anal and posterior anal sectors, evident (Fig. 24E). Legs: tarsomere 1 of pro-, meso- and metathoracic legs is longer than 2. ***Abdomen*.** Integument shiny, punctured, with long dense setae, sternite 7 with margin sinuate, sternite 8 with margin notched; aedeagus with three teeth at the inner apex of paramere (Fig. 24F–H).

#### Female and immatures.

Unknown.

#### Distribution.

Mexico: Mexico City (Fig. 19).

#### Additional material examined.

“MEXICO: Ciudad de Mexico/Jardín Botánico, /19°19'10" N 99° 11'37.25"/ W, 2321 m.a.s.l. 19-X-2017/ V. Vega-Badillo y S. Zaragoza-Caballero” (2) | CNIN; MEXICO: Ciudad de Mexico/ Jardín Botánico, /19°19'10" N 99° 11'37.25" /W, 2321 m.a.s.l. 25-VIII-2017/ V. Vega-Badillo y S. Zaragoza-Caballero (1) | CNIN; MEXICO: Ciudad de Mexico/ Jardín Botánico/ 19°19'10" N 99° 11'37.25" /W, 2321 m.a.s.l. 27-VIII-2017/ V. Vega-Badillo y S. Zaragoza-Caballero (1) | CNIN.

### Cenophengus punctatissimus

Taxon classificationAnimaliaColeopteraPhengodidae

Wittmer, 1976

579B2A1E-1F85-5BC1-A368-1617FAABC102

Fig. 25A–H

Cenophengus punctatissimus Wittmer, 1976: 452.

#### Type locality.

San Luis Potosí, Mexico.

#### Type material examined.

***Holotype*** ♂: MEXICO: “2 km S Tamazunchale, / San Luis Potosí (R. 1 km 363) / 31-V-1948, 700 ft / tropical canyon-jungle” “at light/ F, Werner/ W. Nutting” “Type No. / 73888/ USNM” | NMNH.

#### Remarks.

*Cenophenguspunctatissimus* is morphologically similar to *C.mboi*, but can be distinguished by the interocular distance. In *C.punctatissimus*, the interocular distance is 2.5 times eye width, whereas in *C.mboi*, it is 3 times eye width. Additionally, in *C.punctatissimus*, the posterior radial vein length is 5.3 times less than the length of MP1+2, whereas in *C.pedregalensis*, it is twice less than the length of MP1+2.

#### Diagnosis.

Body dark brown, integument chagreened, head less wide than the pronotum, antennae long, more than twice the length of pronotum, antennal rami twice as long as the respective antennomere and each elytron 5.4 times as long as wide; aedeagus with three teeth at the inner apex of paramere.

#### Redescription.

**Figure 25.**
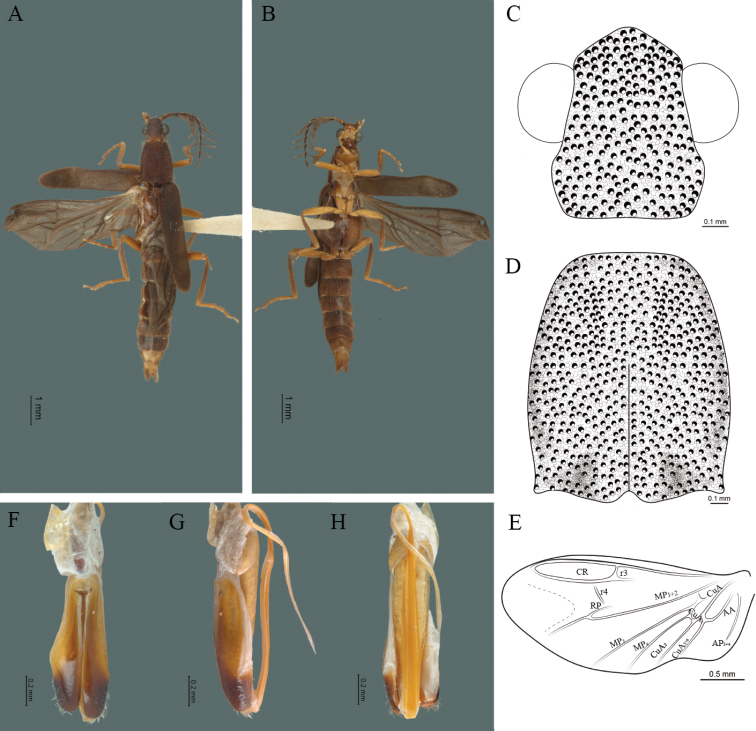
Cenophenguspunctatissimus Wittmer, 1976, male. Habitus: **A** dorsal **B** ventral **C** head dorsal **D** pronotum dorsal **E** hind wing. Wing venation: CR = Radial Cell; r3 = radial 3 vein; r4 = radial 4 vein; RP = Posterior Radial vein; MP1+2 = Posterior Median vein; CuA = Cubital vein; AA and AP = Anterior and Posterior Anal veins. Aedeagus: **F** dorsal view **G** lateral view **H** ventral view.

**Male.** Body length 10.5 mm, maximum body width 1.0 mm (pronotum). Body dark brown, except for buccal parts, coxa, trochanter, femur and two last sternites yellowish-coloured (Fig. 25A, B). ***Head*.** Wider (0.91 mm) than long (0.8 mm) (Fig. 25C), at eye level, less wide than the pronotum, integument chagreened, punctures 2.5 times as long as eye facets and separated by approximately 0.5 punctured diameters, each puncture bearing a yellow-brown seta; interantennal distance (0.10 mm) less than the length of antennomere 1 (0.20 mm); eyes 1/2 as long as head in lateral view, longer (0.35 mm) than wide (0.21 mm); interocular distance (0.52 mm) 2.5 times eye width; antennae long (2.42 mm) more than twice the length of pronotum; antennomere 1 (0.20 mm) as long as the next two combined, antennomere 3 cup-shaped, 4 (0.20 mm) shorter than the following antennomeres, 5 to 11 about equal in length (0.25), 12 (terminal) (0.30 mm), antennal rami lanceolate in lateral view, twice as long as the respective antennomere; terminal maxillary palpomere robust, securiform (0.40 mm), as long as the preceding three combined (0.40 mm); terminal labial palpomere spindle-shaped (0.15 mm), 3 times as long as preceding one (0.05 mm). ***Thorax*.** Pronotum longer (1.40 mm) than wide (1.0 mm) (Fig. 25D); integument chagreened, punctures 2.5 times as long as eye facets and separated by approximately 1 punctured diameter, each puncture bearing a yellow-brown seta, disc convex, with a longitudinal carina in posterior portion of pronotum strongly visible, with a length exceeding the median length of the pronotum, posterior margin curved with middle notch, sides almost straight, anterior angles rounded and posterior angles acute; mesosternal suture incomplete; scutellum with posterior margin rounded; each elytron 5.4 times as long (3.48 mm) as wide (0.64 mm), convex, without longitudinal costae, elytral apex blunted; hind wings with posterior radial vein (RP) length 5.3 times less than the length of MP1+2, radial cell closed, r3 vein present, r4 vein reduced (reaching the RP or the radial cell), those of the anterior anal and posterior anal sectors, evident (Fig. 25E). Legs: tarsomere 1 of pro-, meso- and metathoracic legs is longer than 2. ***Abdomen*.** Integument shiny, punctured, with long dense setae, sternite 7 with margin sinuate, sternite 8 with margin concave; aedeagus with three teeth at the inner apex of paramere (Fig. 25F–H).

#### Female and immatures.

Unknown.

#### Distribution.

Mexico: San Luis Potosí (Fig. 19).

### Cenophegus saasil

Taxon classificationAnimaliaColeopteraPhengodidae

Vega-Badillo, Morrone & Zaragoza-Caballero sp. nov.

7DB01BA5-75B3-5932-A43C-7B95C4049D2B

http://zoobank.org/7200B416-7A3D-421E-98F8-1D03CB4AACC6

Fig. 26A–H

#### Type locality.

Honduras.

#### Type material.

***Holotype*** ♂: HONDURAS: “HND: CR; Cusuco National Park; / Cantiles 15.5077°N 88.2336°W/ 2028 m 19-25 Jun. 2014 Michelle/ D’Souza” “Barcode of Life DNA/ Voucher specimen/ Sample ID/ BIOUG19147-G03 /ProcessID/ GMHKB847-15” | CBG.

#### Remarks.

*Cenophegussaasil* is morphologically similar to *C.wittmeri*, but can be distinguished by pronotum colouration and r3 vein. In *C.saasil*, the pronotum colouration is uniform, whereas in *C.wittmeri*, it is dark brown near mid-line; the r3 vein is present in *C.wittmeri* and absent in *C.saasil*.

#### Diagnosis.

Body pale yellow, integument chagreened, head as wide as the pronotum, antennae long, more than twice the length of pronotum, antennal rami twice as long as the respective antennomere and each elytron 4.6 times as long as wide; aedeagus with three teeth at the inner apex of paramere.

#### Description.

**Figure 26.**
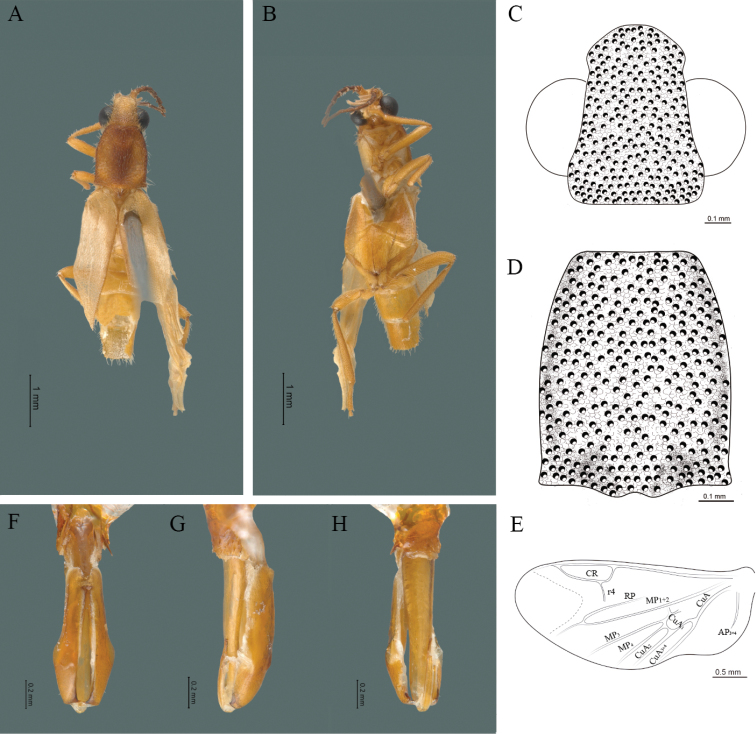
*Cenophengussaasil* Vega-Badillo, Morrone & Zaragoza-Caballero, sp. nov., male. Habitus: **A** dorsal **B** ventral **C** head dorsal **D** pronotum dorsal **E** hind wing. Wing venation: CR = Radial Cell; r4 = radial 4 vein; RP = Posterior Radial vein; MP1+2 = Posterior Median vein; CuA = Cubital vein; AA and AP = Anterior and Posterior Anal veins. Aedeagus: **F** dorsal view **G** lateral view **H** ventral view.

**Male.** Body length 9.50 mm, maximum body width 0.90 mm (pronotum). Body pale yellow, except for the antennae and stripe on pronotum brown (Fig. 26A, B). ***Head*.** Wider (0.90 mm) than long (0.65 mm) (Fig. 26C), at eye level, as wide as the pronotum, integument chagreened, punctures twice as long as eye facets and separated by approximately 1 punctured diameter, each puncture bearing a yellow seta; interantennal distance (0.06 mm) less than the length of antennomere 1 (0.24 mm); eyes 1/2 as long as head in lateral view, longer (0.41 mm) than wide (0.21 mm); interocular distance (0.42 mm) twice as long as eye width; antennae long (3.10 mm) more than twice the length of pronotum; antennomere 1 (0.24 mm) longer than next two combined, antennomere 3 cup-shaped, 4 to 11 about equal in length (0.30 mm), 12 (terminal) (0.33 mm), antennal rami lanceolate in lateral view, twice as long as the respective antennomere; maxillary palpomeres of the holotype lost; labial palpomere 1 (0.03 mm). ***Thorax*.** Pronotum longer (1.10 mm) than wide (0.90 mm) (Fig. 26D); integument chagreened, punctures twice as long as facets and separated by approximately 1.5 punctured diameters, each puncture bearing a yellow seta, disc convex, weakly elevated dorsally, forming a small depression in the basal part of each side, posterior margin curved with middle notch, sides almost straight, anterior angles rounded and posterior angles acute; mesosternal suture incomplete; scutellum of the holotype lost; each elytron 4.6 times as long (3.40 mm) as wide (0.74 mm), convex, with longitudinal costae, elytral apex rounded; hind wings with posterior radial vein (RP) length twice less than the length of MP1+2, radial cell closed, r3 vein absent, r4 vein developed (reaching the radial cell), those of the anterior anal and posterior anal sectors, present (Fig. 26E). Legs: tarsomeres 1 and 2 of the prothoracic legs with a similar length and tarsomere 1 of meso- and metathoracic legs is longer than 2. ***Abdomen*.** Integument shiny, punctured, with long dense setae, sternite 7 with margin sinuate, sternite 8 with margin notched; aedeagus with three teeth at the inner apex of paramere (Fig. 26F–H).

#### Female and immatures.

Unknown.

#### Distribution.

Honduras (Fig. 19).

#### Etymology.

The term sáasil means glow in the Maya language, which is spoken in Honduras.

### Cenophengus sonoraensis

Taxon classificationAnimaliaColeopteraPhengodidae

Zaragoza-Caballero, 2008

396DF461-4AE9-5878-966B-A824167F463E

Fig. 27A–E

Cenophengus sonoraensis Zaragoza-Caballero 2008: 155.

#### Type locality.

Sonora, Mexico.

#### Type material examined.

***Holotype*** ♂: “MEXICO: Sonora, 36.6 / km SE Tecoripa, La / Barranca, 28°34'40.1"N, / 109° 39' 40.1"O. Alt. 562 m. / TL 1 16-08-2004 / Col. S. Zaragoza” | CNIN.

#### Remarks.

*Cenophengussonoraensis* is sister to *C.pallidus* (Vega-Badillo et al. 2021b), but can be distinguished by the colour of the body and the interocular distance. In *C.sonoraensis*, it has a pale brown body, whereas in *C.pallidus*, it is yellow. The interocular distance is twice as long as eye width in *C.sonoraensis*, in *C.pallidus*, it is 1.5 longer than eye width. Additionally, in *C.sonoraensis*, disc weakly elevated dorsally, forming a small depression in the basal part of each side, whereas in *C.pallidus*, it has a longitudinal carina.

#### Diagnosis.

Body pale brown, integument chagreened, head wider than the pronotum, antennae less than twice the length of the pronotum, antennal rami twice as long as the respective antennomere and each elytron 4.1 times as long as wide.

#### Redescription.

**Figure 27.**
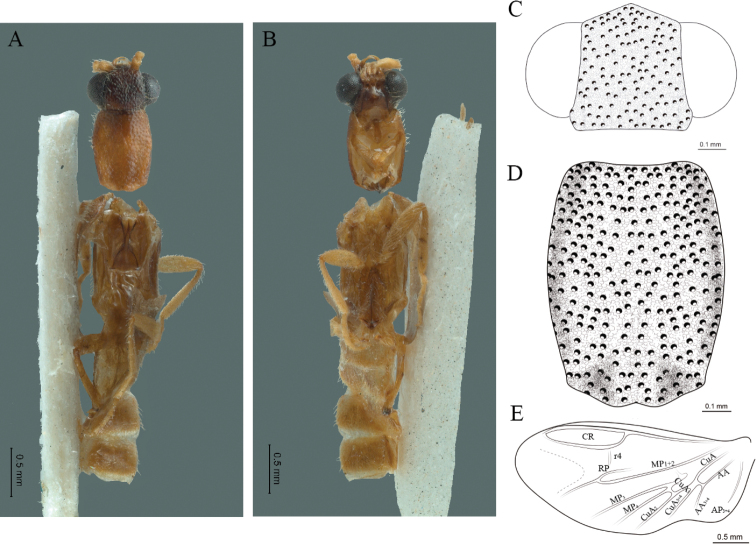
*Cenophengussonoraensis* Zaragoza-Caballero, 2008, male. Habitus: **A** dorsal **B** ventral **C** head dorsal **D** pronotum dorsal **E** hind wing. Wing venation: CR = Radial Cell; r4 = radial 4 vein; RP = Posterior Radial vein; MP1+2 = Posterior Median vein; CuA = Cubital vein; AA and AP = Anterior and Posterior Anal veins.

**Male.** Body length 4.20 mm, maximum body width 0.55 mm (pronotum). Body pale brown, except for head dark brown (Fig. 27A, B). ***Head*.** Wider (0.69) than long (0.35 mm) (Fig. 27C), at eye level, wider than the pronotum, integument chagreened, punctures as long as eye facets and separated by approximately 2 punctured diameters, each puncture bearing a yellow-brown seta; interantennal distance (0.07 mm) less than the length of antennomere 1 (0.15 mm); eyes 3/4 as long as head in lateral view, longer (0.30 mm) than wide (0.20 mm); interocular distance (0.38 mm) twice as long as eye width; antennae short (1.20 mm) less than twice the length of the pronotum; antennomere 1 (0.15 mm) is longer than the next two combined, antennomere 3 cup-shaped, 4 (0.10 mm) shorter than following antennomeres, 5 to 11 about equal in length (0.11 mm), 12 (terminal) (0.12 mm), antennal rami lanceolate in lateral view, twice as long as the respective antennomere; terminal maxillary palpomere robust, securiform (0.25 mm), as long as the preceding three combined (0.25 mm); terminal labial palpomere spindle-shaped (0.10 mm), 3 times as long as preceding one (0.03 mm). ***Thorax*.** Pronotum longer (1.40 mm) than wide (1.0 mm) (Fig. 27D); integument chagreened, punctures as long as eye facets and separated by approximately 2 punctured diameters, each puncture bearing a yellow-brown seta, disc convex, weakly elevated dorsally forming a small depression in the basal part of each side, sides almost straight, anterior and posterior angles rounded; mesosternal suture complete; scutellum with posterior margin rounded; each elytron 4.1 times as long (1.40 mm) as wide (0.34 mm), convex, without longitudinal costae, elytral apex rounded; hind wings with posterior radial vein (RP) length 3.5 times less than the length of MP1+2, radial cell closed, r3 vein absent, r4 vein reduced (not reaching the RP and the radial cell), those of the anterior anal and posterior anal sectors, evident (Fig. 27E). Legs: tarsomere 1 of pro-, meso- and metathoracic legs is longer than 2. ***Abdomen*.** Integument shiny, punctured, with long dense setae, sternite 7 with margin sinuate, last sternite with margin notched; aedeagus of the holotype lost.

#### Female and immatures.

Unknown.

#### Distribution.

Mexico: Sonora (Fig. 19).

### Cenophengus tsiik

Taxon classificationAnimaliaColeopteraPhengodidae

Vega-Badillo, Morrone & Zaragoza-Caballero sp. nov.

A5F56C4D-1CA4-5EA8-B36C-52150EC10D0D

http://zoobank.org/32433840-EF27-4AEE-A4BB-3B64A250BE2E

Fig. 28A–H

#### Type locality.

Belize.

#### Type material.

***Holotype*** ♂: “BELIZE: Orange Walk Dist/ Rio Bravo Conserv. Area/18. IV. 1995; PKKovarik &/ JShuey colrs; light trap” “From the Michael Ivie Collection” | NMNH.

#### Remarks.

*Cenophengustsiik* is morphologically similar to *C.cuicatlaensis*, but can be distinguished by the interocular distance and the terminal maxillary palpomere. In *C.tsiik*, interocular distance is 3 times eye width, whereas in *C.cuicatlaensis*, it is twice as long as eye width. Terminal maxillary palpomere is shorter than the preceding three combined in *C.tsiik*, in *C.cuicatlaensis*, it is longer than the preceding three combined.

#### Diagnosis.

Integument chagreened, head almost as wide as the pronotum, antennae less than twice the length of the pronotum, antennal rami 1.5 times the respective and antennomere, each elytron 2.8 times as long as wide; aedeagus with three teeth at the inner apex of paramere.

#### Description.

**Figure 28.**
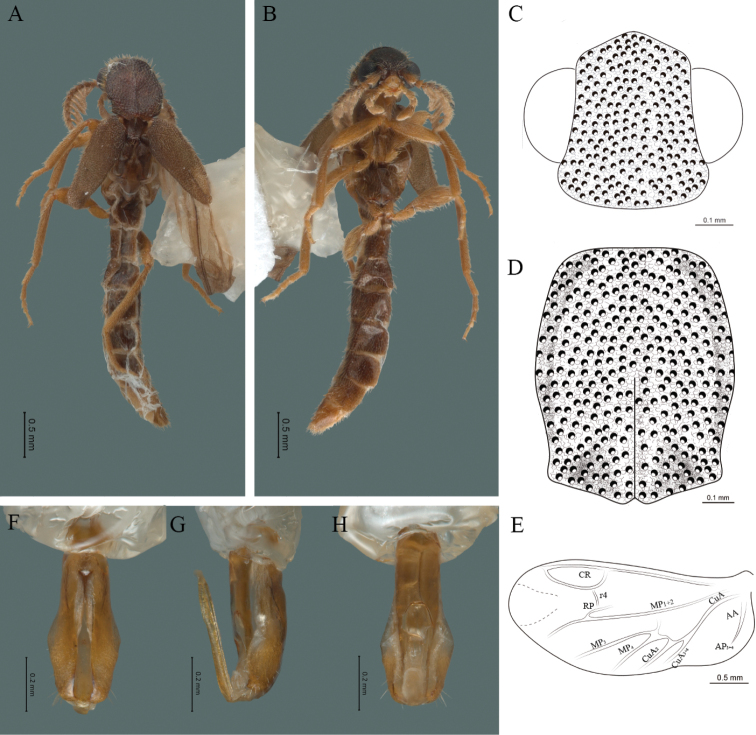
*Cenophengustsiik* Vega-Badillo, Morrone & Zaragoza-Caballero, sp. nov., male. Habitus: **A** dorsal **B** ventral **C** head dorsal **D** pronotum dorsal **E** hind wing. Wing venation: CR = Radial Cell; r4 = radial 4 vein; RP = Posterior Radial vein; MP1+2 = Posterior Median vein; CuA = Cubital vein; AP = Posterior Anal vein. Aedeagus: **F** dorsal view **G** lateral view **H** ventral view.

**Male.** Body length 5.50 mm, maximum body width 0.65 mm (pronotum). Body dark brown, except for the antennae buccal parts, legs and the two last sternites are pale brown to yellow (Fig. 28A, B). ***Head*.** Wider (0.68 mm) than long (0.55 mm) (Fig. 28C), at eye level, almost as wide as the pronotum (0.65 mm), integument chagreened, punctures twice as long as eye facets and separated by approximately 0.5 punctured diameters, each puncture bearing a yellow-brown seta; interantennal distance (0.09 mm) less than the length of antennomere 1 (0.18 mm); eyes 1/2 as long as head in lateral view, longer (0.30 mm) than wide (0.13 mm); interocular distance (0.40 mm) 3 times eye width; antennae short (1.58 mm less than twice the length of the pronotum; antennomere 1 (0.18 mm) is longer than the next two combined (0.10 mm), antennomere 3 cup-shaped, 4 (0.10 mm) shorter than following antennomeres, 5 to 11 about equal in length (0.15 mm), 12 (terminal) (0.17 mm), antennal rami lanceolate in lateral view, 1.5 times the respective antennomere; terminal maxillary palpomere robust, securiform (0.25 mm), shorter than the preceding three combined; terminal labial palpomere spindle-shaped (0.09 mm), 3 times as long as preceding one (0.03 mm). ***Thorax*.** Pronotum longer (0.80 mm) than wide (0.65 mm) (Fig. 28D); integument chagreened, punctures twice as long as eye facets and separated by approximately 1 punctured diameter, each puncture bearing a yellow-brown seta, disc convex, with a longitudinal carina in posterior portion of pronotum strongly visible, with a length equal to the median length of the pronotum, posterior margin curved with middle notch, sides almost straight, anterior and posterior angles rounded; mesosternal suture complete; scutellum with posterior margin rounded; each elytron 2.8 times as long (1.12 mm) as wide (0.40 mm), convex, without longitudinal costae, elytral apex rounded; hind wings with posterior radial vein (RP) length 5 times less than the length of MP1+2, radial cell closed, r3 vein absent, r4 vein developed (reaching the radial cell), those of the anterior anal and posterior anal sectors, slightly evident (Fig. 28E). Legs: tarsomere 1 of pro-, meso- and metathoracic legs is longer than 2. ***Abdomen*.** Integument shiny, punctured, with long dense setae, sternite 7 with margin sinuate, sternite 8 with margin notched; aedeagus with three teeth at the inner apex of paramere (Fig. 28F–H).

#### Female and immatures.

Unknown.

#### Distribution.

Belize (Fig. 19).

#### Etymology.

The term “tsiik” means honour in the Maya language, which is spoken in some regions of Belize.

### Cenophengus tupae

Taxon classificationAnimaliaColeopteraPhengodidae

Vega-Badillo et al. 2021

A9A8831F-6CC8-55AC-8327-3B3C378B0F06

Fig. 29A–H

Cenophengus tupae Vega-Badillo et al. 2021a: 232.

#### Type locality.

San Luis Potosí, Mexico.

#### Type material examined.

***Holotype*** ♂: “Mexico, San Luis Potosí, / Tamasopo. Cerro al noroeste/ del cafetal, 01-06-15, / N 21°55.47' W 99°24.95' Col. / Jessica Ríos” | CNIN. ***Paratype*** ♂: same data | CNIN.

#### Remarks.

*Cenophengustupae* is morphologically similar to *C.howdeni*, but can be distinguished by the length of antennomere 1, the pronotal disc and interocular distance. In *C.tupae*, antennomere 1 is longer than the next two combined, whereas in *C.howdeni*, it is shorter than the next two combined. The pronotal disc is convex, weakly elevated dorsally, forming a small depression in the basal part of each side, in *C.howdeni* it has a groove along mid-line. The interocular distance is 3.0 times eye width in *C.howdeni*, in *C.tupae*, it is twice as long as eye width.

#### Diagnosis.

Body brown except for antennae yellow-brown, integument chagreened, antennae long, more than twice the length of pronotum, antennal rami 3 times as long as the respective antennomere, head almost as wide as the pronotum and each elytron 4.1 times as long as wide; aedeagus trilobed with three teeth at the inner apex of paramere.

#### Redescription.

**Figure 29.**
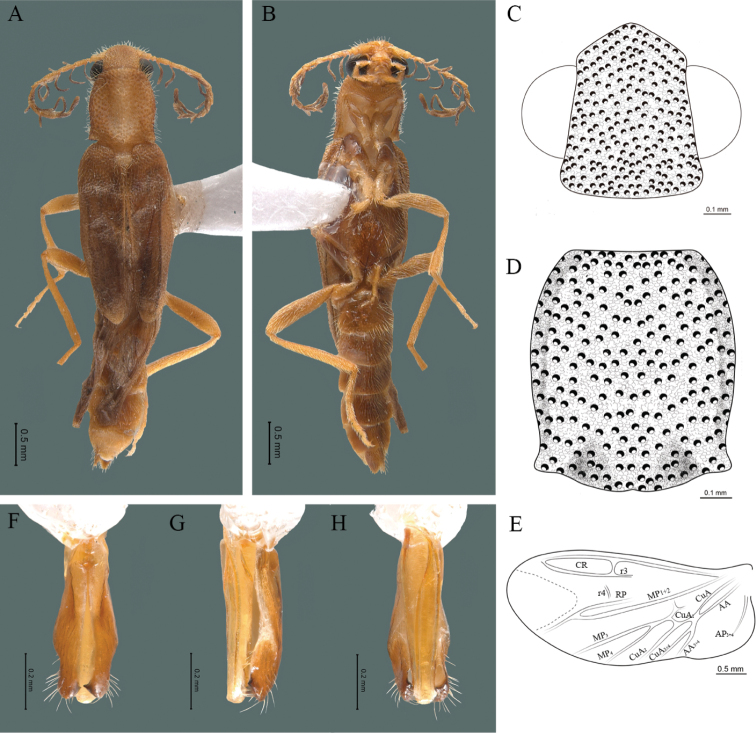
*Cenophengustupae* Vega-Badillo et al. 2021, male. Habitus: **A** dorsal **B** ventral **C** head dorsal **D** pronotum dorsal **E** hind wing. Wing venation: CR = Radial Cell; r3 = radial 3 vein; r4 = radial 4 vein; RP = Posterior Radial vein; MP1+2 = Posterior Median, vein; CuA = Cubital vein; AA and AP = Anterior and Posterior Anal veins. Aedeagus: **F** dorsal view **G** lateral view **H** ventral view.

**Male.** Body length 4.0–5.2 mm, maximum body width 0.60–0.70 mm (pronotum). Body brown, except for antennae and stripe on pronotum yellow-brown (Fig. 29A, B). ***Head*.** Wider (0.60–0.71 mm) (0.655 ± 0.077 mm, n = 2) than long (0.40–0.50 mm) (0.45 ± 0.07 mm, n = 2) (Fig. 29C), at eye level, almost as wider as the pronotum, integument chagreened, punctures 1.5 times as long as eye facets and separated by approximately 1.5 punctured diameters, each puncture bearing a yellow-brown seta; interantennal distance (0.04–0.05 mm) (0.045 ± 0.007 mm, n = 2) less than the length of antennomere 1 (0.16–0.18 mm) (0.17 ± 0.014 mm, n = 2); eyes 1/2 as long as head in lateral view, longer (0.27–0.30 mm) (0.285 ± 0.021 mm, n = 2) than wide (0.18–0.23 mm) (0.20 ± 0.035 mm, n = 2); interocular distance (0.35–0.40 mm) (0.375 ± 0.035 mm, n = 2) twice as long as eye width; antennae long (1.54–1.60 mm) (1.58 ± 0.042 mm, n = 2), more than twice the length of pronotum; antennomere 1 (0.16–0.18 mm) (0.17 ± 0.014 mm, n = 2) longer than the next two combined, antennomere 3 cup-shaped, 4 (0.12–0.13 mm) (0.125 ± 0.007 mm, n = 2) shorter than the following antennomeres, 5 to 11 about equal in length (0.14–0.15 mm) (0.145 ± 0.007 mm, n = 2), 12 (terminal) (0.17–0.20) (0.18 ± 0.021 mm, n = 2), antennal rami lanceolate in lateral view, 3 times as long as the respective antennomere; terminal maxillary palpomere robust, securiform (0.15–0.16 mm) (0.155 ± 0.007 mm, n = 2), shorter than the preceding three combined; terminal labial palpomere spindle-shaped (0.09–0.10 mm) (0.095 ± 0.007 mm, n = 2), 3 times as long as the preceding one (0.03) (0.03 ± 0 mm, n = 2). ***Thorax*.** Pronotum longer (0.72–0.80 mm) (0.76 ± 0.056 mm, n = 2) than wide (0.60–0.71 mm) (0.65 ± 0.077 mm, n = 2) (Fig. 29D); integument chagreened, punctures 1.5 times as long as eye facets and separated by approximately 1.5 punctured diameters, each puncture bearing a yellow-brown seta, convex disc, weakly elevated dorsally, forming a small depression in the basal part of each side, posterior margin almost straight with middle notch, sides almost straight, anterior angles rounded and posterior angles acute; mesosternal suture complete; scutellum with posterior margin rounded; each elytron 4.1 times as long (1.57–1.90 mm) (1.73 ± 0.233 mm, n = 2) as wide (0.40–0.46 mm) (0.43 ± 0.021 mm, n = 2), convex, without longitudinal costae, elytral apex rounded; hind wings with posterior radial vein (RP) length 3 times less than the length of MP1+2, radial cell closed, r3 vein present, r4 vein reduced (not reaching the RP or the radial cell), those of the anterior anal and posterior anal sectors, evident (Fig. 29E). Legs: tarsomeres 1 and 2 of the prothoracic legs with a similar length and tarsomere 1 of meso- and metathoracic legs is longer than 2. ***Abdomen*.** Integument shiny, punctured, with long dense setae, sternite 7 with margin sinuate, sternite 8 with margin notched; aedeagus trilobed with three teeth at the inner apex of paramere (Fig. 29F–H).

#### Female and immatures.

Unknown.

#### Distribution.

Mexico: San Luis Potosí (Fig. 19).

### Cenophengus villae

Taxon classificationAnimaliaColeopteraPhengodidae

Zaragoza-Caballero, 1984

277B116A-B532-5C7E-BCDC-8A265FB41F77

Fig. 30A–E

Cenophengus villae Zaragoza-Caballero, 1984: 198.

#### Type locality.

Veracruz, Mexico.

#### Type material examined.

***Holotype*** ♂: Mexico: “Veracruz, Metlac, / VI. 76 /900 m.a.s.l. / S. Zaragoza / Col. Noc.” | CNIN.

#### Remarks.

*Cenophengusvillae* is sister to *C.brunneus* (Vega-Badillo et al. 2021b), but can be distinguished by the interocular distance: in *C.villae*, it is 4 times eye width, whereas in *C.brunneus*, it is 3.5 times longer. Additionally, in *C.villae*, the pronotal disc has a longitudinal carina, whereas in *C.brunneus*, the disc is without a longitudinal carina.

#### Diagnosis.

Body dark brown, integument chagreened, antennae less than twice the length of the pronotum, antennal rami 1.5 times the respective antennomere and each elytron 4.3 times as long as wide.

#### Redescription.

**Figure 30.**
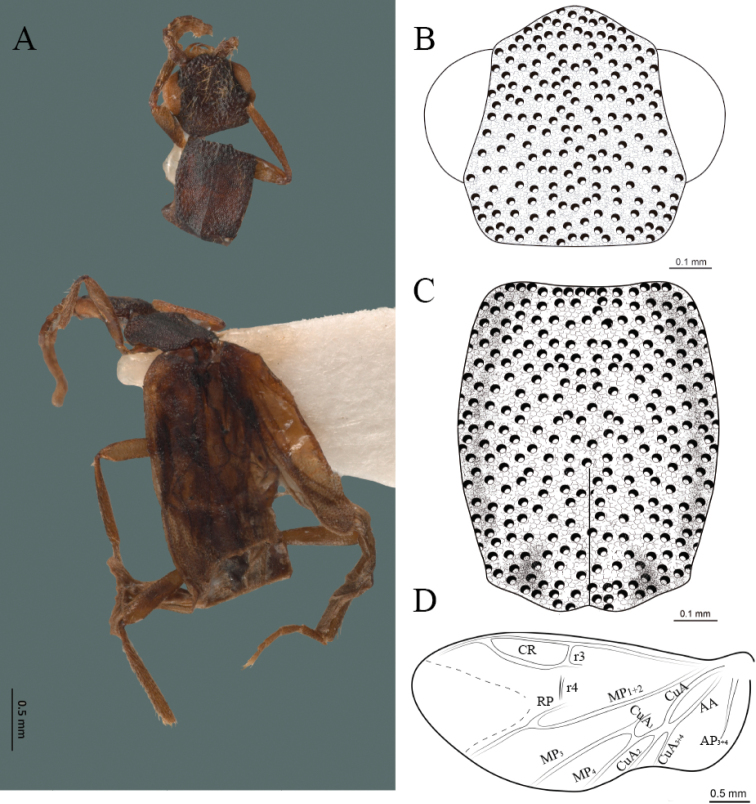
*Cenophengusvillae* Zaragoza-Caballero, 1984, male. Habitus: **A** dorsal **B** head dorsal **C** pronotum dorsal **D** hind wing. Wing venation: CR = Radial Cell; r3 = radial 3 vein; r4 = radial 4 vein; RP = Posterior Radial vein; MP1+2 = Posterior Median vein; CuA = Cubital vein; AA and AP = Anterior and Posterior Anal veins.

**Male.** Body length 4.20 mm, maximum body width 0.51 mm (pronotum). Body dark brown, except for legs yellowish (Fig. 30A). ***Head*.** Wider (0.65 mm) than long (0.50 mm) (Fig. 30B), at eye level, less wide than the pronotum, integument chagreened, punctures twice as long as eye facets and separated by approximately 1 punctured diameter, each puncture bearing a brown seta; interantennal distance (0.05 mm) less than the length of antennomere 1 (0.13 mm); eyes 1/2 as long as head in lateral view, longer (0.28 mm) than wide (0.15 mm); interocular distance (0.70 mm) 4 times eye width; antennae short (1.50 mm) less than twice the length of the pronotum; antennomere 1 (0.13 mm) as long as the next two combined, antennomere 3 cup-shaped, 4 (0.11 mm) shorter than following antennomeres, 5 to 11 about equal in length (0.15 mm), 12 (terminal) (0.20 mm), antennal rami lanceolate in lateral view, 1.5 times the respective antennomere; terminal maxillary palpomere robust, securiform (0.40 mm), as long as the preceding three combined; terminal labial palpomere spindle-shaped (0.06 mm), 3 times as long as preceding one (0.02 mm). ***Thorax*.** Pronotum longer (0.70 mm) than wide (0.51 mm) (Fig. 30C); integument chagreened, punctures twice as long as eye facets and separated by approximately 1 punctured diameter, each puncture bearing a brown seta, disc convex, with a longitudinal carina in posterior portion of pronotum strongly visible, with a length that does not reach the median length of the pronotum, posterior margin curved, sides almost straight, anterior and posterior angles rounded; mesosternal suture complete; scutellum with posterior margin rounded; each elytron 4.3 times as long (1.64 mm) as wide (0.38 mm), convex, without longitudinal costae, elytral apex rounded; hind wings with posterior radial vein (RP) length 5 times less than the length of MP1+2, radial cell closed, r3 vein present, r4 vein reduced (not reaching the RP or the radial cell, those of the anterior anal and posterior anal sectors, evident (Fig. 30D). Legs: tarsomeres 1 and 2 of pro- and mesothoracic legs with a similar length, tarsomere 1 of metathoracic legs is longer than 2. ***Abdomen*.** Integument shiny, punctured, with long dense setae, sternite 7 with margin sinuate, sternite 8 with margin notched; aedeagus of the holotype lost.

#### Female and immatures.

Unknown.

#### Distribution.

Mexico: Veracruz (Fig. 19).

### Cenophengus wittmeri

Taxon classificationAnimaliaColeopteraPhengodidae

Zaragoza-Caballero, 1984

A9F8BFAE-4D04-5210-A7C4-3C7FB0939636

Fig. 31A–H

Cenophengus wittmeri Zaragoza-Caballero, 1984: 196.

#### Type locality.

Puebla, Mexico.

#### Type material examined.

***Holotype*** ♂: Mexico: “Puebla, Plata, / VII-75 / 960 m.a.s.l. / J. Bueno / Col. Noc.” |CNIN. ***Paratype*** ♂: “Mexico: Hidalgo, Ixtlahuaco / Alt. 1550 m. 17- 07- 1983 / Luz incandescente amarilla / colecta nocturna, Bosque / Mesófilo de montaña. / Col. R. Terrón” (1) | CNIN.

#### Remarks.

*Cenophenguswittmeri* is morphologically similar to *C.marmoratus*, but can be distinguished by the colour of the body and the terminal maxillary palpomere. In *C.wittmeri*, its body is brown, except for the middle part of the pronotum that is dark brown, whereas in *C.marmoratus*, it is yellow or pale brown, the pronotum partially interrupted by darker brown spots. The terminal maxillary palpomere is as long as the preceding three combined in *C.wittmeri*, in *C.marmoratus*, it is shorter than the preceding three combined. Additionally, in *C.wittmeri*, the posterior radial vein length is twice less than the length of MP1+2, whereas in *C.marmoratus*, it is 1.6 times less than the length of MP1+2.

#### Diagnosis.

Body brown, except for middle part of pronotum, integument chagreened, head almost as wide as the pronotum, antennae long, more than twice the length of pronotum, antennal rami twice as long as the respective antennomere and each elytron 3.7 times as long as wide; aedeagus with three teeth at the inner apex of paramere.

#### Redescription.

**Figure 31.**
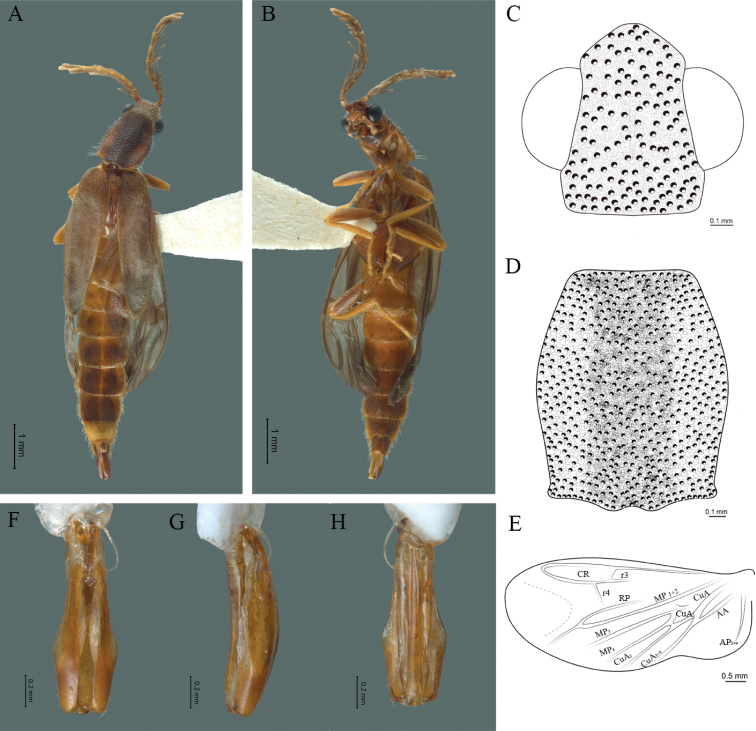
*Cenophenguswittmeri* Zaragoza-Caballero, 1984, male. Habitus: **A** dorsal **B** ventral **C** head dorsal **D** pronotum **E** hind wing. Wing venation: CR = Radial Cell; r3 = radial 3 vein; r4 = radial 4 vein; RP = Posterior Radial vein; MP1+2 = Posterior Median vein; CuA = Cubital vein; AA and AP = Anterior and Posterior Anal veins. Aedeagus: **F** dorsal view **G** lateral view **H** ventral view.

**Male.** Body length 5.80–9.20 mm, maximum body width 0.87–1.04 mm (pronotum). Body brown, except for middle part of pronotum and last two sternites dark brown, elytral apex whitish (Fig. 31A, B). ***Head*.** Wider (0.83–0.90 mm) (0.85 ± 0.034 mm, n = 4) than long (0.62–0.80 mm) (0.74 ± 0.081 mm, n = 4) (Fig. 31C), at eye level, almost as wide as the pronotum, integument chagreened with punctures twice as large as eye facets and separated by approximately 2 punctured diameters, each puncture bearing a yellow-brown seta; interantennal distance (0.09–0.11 mm) (0.1 ± 0.009 mm, n = 4) less than the length of antennomere 1 (0.22–0.28 mm) (0.25 ± 0.0.21 mm, n = 4); eyes 1/2 as long as head in lateral view, longer (0.35–0.40 mm) (0.38 ± 0.024 mm, n = 4) than wide (0.20–0.24 mm) (0.21 ± 0.02 mm, n = 4); interocular distance (0.43–0.50 mm) (0.46 ± 0.029 mm, n = 4) twice as long as eye width; antennae long (2.50–3.09 mm) (2.07 ± 0.373 mm, n = 4), more than twice the length of pronotum; antennomere 1 (0.22–0.28 mm) (0.25 ± 0.0.21 mm, n = 4) longer than the next two combined, antennomere 3 cup-shaped, 4 (0.15–0.18 mm) (0.26 ± 0.046 mm, n = 4) shorter than the following antennomeres, 5 to 11 about equal in length (0.22–0.30 mm) (0.27 ± 0.057 mm, n = 4), 12 (terminal) (0.30–0.40) (0.36 ± 0.047 mm, n = 4), antennal rami lanceolate in lateral view, twice as long as the respective antennomere; terminal maxillary palpomere robust, securiform (0.22–0.35 mm) (0.29 ± 0.075 mm, n = 4), shorter than the preceding three combined; terminal labial palpomere spindle-shaped (0.12–0.14 mm) (0.13 ± 0.01 mm, n = 4), twice as long as preceding one (0.05–0.06 mm) (0.005 ± 0.458, n = 4). ***Thorax*.** Pronotum longer (0.94–1.30 mm) (1.04 ± 0.173 mm, n = 4) than wide (0.87–1.04 mm) (0.94 ± 0.081 mm, n = 4) (Fig. 31D); integument chagreened, punctures twice as long as eye facets and separated by approximately 1 punctured diameter, each puncture bearing a yellow-brown seta, disc convex, weakly elevated dorsally forming a small depression in the basal part of each side, posterior margin curved with middle notch, sides almost straight, anterior and posterior angles rounded; mesosternal suture incomplete; scutellum with posterior margin rounded; each elytron 3.5 times as long (1.7–3.5 mm) (2.55 ± 0.741 mm, n = 4) as wide (0.48–1.0 mm) (0.74 ± 0.216 mm, n = 4), convex, without longitudinal costae, elytral apex rounded; hind wings with posterior radial vein (RP) length twice less than the length of MP1+2, radial cell closed, r3 vein present, r4 vein developed (reaching the radial cell), those of the anterior anal and posterior anal sectors, evident (Fig. 31E). Legs: tarsomere 1 of pro-, meso- and metathoracic legs is longer than 2. ***Abdomen*.** Integument shiny, punctured, with long dense setae, sternite 7 with margin sinuate, sternite 8 with margin notched; aedeagus with three teeth at the inner apex of paramere (Fig. 31F–H).

#### Female and immatures.

Unknown.

#### Distribution.

Mexico: Puebla, Hidalgo and Veracruz (Fig. 19).

#### Additional material examined.

“MEXICO: Hidalgo, Tlanchinol/TiV-1, 1 a 22 - X -2011/ Cols. J. Márquez y J. Asiain” (1) | CNIN; “MEXICO: Veracruz, Hwy. /131, Altotonga /7000´20 Aug. 1982 C & / L.O’ Brien & G. Wibmer “(1) | FSCA.

### Cenophengus xiinbali

Taxon classificationAnimaliaColeopteraPhengodidae

Vega-Badillo et al. 2021

1CA9E05F-8BC4-5364-BE33-C9A095523D7B

Fig. 32A–H

Cenophengus xiinbali Vega-Badillo et al. 2021a: 233.

#### Type locality.

Puerta Parada, Guatemala.

#### Type material examined.

***Holotype*** ♂: “Guatemala: Guatemala Dept. / Puerta Parada km 14.5 carr. a / El Salvador 1840 m alt. / 8-15/VI/2013 Col. J.C Schuster” | CNIN. ***Paratype*** ♂: same data | CNIN.

#### Remarks.

*Cenophengusxiinbali* is morphologically similar to *C.longicollis*, but can be distinguished by the interocular distance and terminal maxillary palpomere. In *C.xiinbali*, the interocular distance is 3.5 times eye width, whereas in *C.longicollis*, it is 3 times longer. The terminal maxillary palpomere is as long as the preceding three combined in *C.xiinbali*, whereas in *C.longicollis*, it is longer than the preceding three combined.

#### Diagnosis.

Integument chagreened, antennae long, more than twice the length of pronotum, antennal rami twice as long as the respective antennomere, terminal maxillary palpomere as long as the preceding three combined and each elytron 4.1 times as long as wide; aedeagus with three teeth at the inner apex of paramere.

#### Redescription.

**Figure 32.**
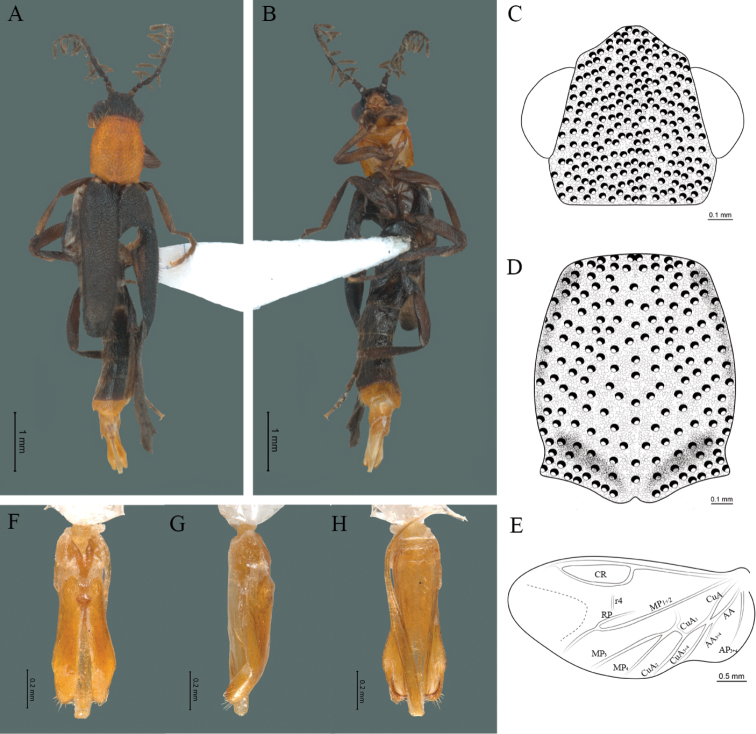
*Cenophengusxiinbali* Vega-Badillo et al. 2021, male. Habitus: **A** dorsal **B** ventral **C** head dorsal **D** pronotum **E** hind wing. Wing venation: CR = Radial Cell; r4 = radial 4 vein; RP = Posterior Radial vein; MP1+2 = Posterior Median vein; CuA = Cubital vein; AA and AP = Anterior and Posterior Anal veins. Aedeagus: **F** dorsal view **G** lateral view **H** ventral view.

**Male.** Body length 8.15–8.30 mm, maximum body width 0.90–0.93 mm (pronotum). Body brown, except for pronotum, legs and two last abdominal segments orange (Fig. 32A, B). ***Head*.** Wider (0.80–81 mm) (0.80 ± 0.007 mm, n = 2) than long (0.72–0.73 mm) (0.725 ± 0.007 mm, n = 2) (Fig. 32C), at eye level, less than the pronotum, integument chagreened, punctures 3 times as long as eye facets and separated by approximately 0.2 punctured diameters, each puncture bearing a yellow-brown seta; interantennal distance (0.10–0.12 mm) (0.11 ± 0.014 mm, n = 2) wider than the length of antennomere 1 (0.17–0.20 mm) (0.18 ± 0.21 mm, n = 2); eyes 1/2 as long as head in lateral view , longer (0.31–0.33 mm) (0.32 ± 0.014 mm, n = 2) than wide (0.13–0.15 mm) (0.14 ± 0.014 mm, n = 2); interocular distance (0.50–0.55 mm) (0.525 ± 0.035 mm, n = 2) 3.5 times eye width; antennae long (2.21–2.30 mm) (2.25 ± 0.056 mm, n = 2), more than twice the length of pronotum; antennomere 1 (0.17–0.20 mm) (0.18 ± 0.21 mm, n = 2) longer than the next two combined, antennomere 3 cup-shaped, 4 (0.14–0.16 mm) (0.15 ± 0.014 mm, n = 2), shorter than the following antennomeres, 5 to 11 about equal in length (0.20–0.22 mm) (0.21 ± 0.014 mm, n = 2), 12 (terminal) (0.25–0.30 mm) (0.27 ± 0.035 mm, n = 2), antennal rami lanceolate in lateral view, twice as long as the respective antennomere; terminal maxillary palpomere robust, securiform (0.30–0.33 mm) (0.31 ± 0.021 mm, n = 2), as long as the preceding three combined; terminal labial palpomere spindle-shaped (0.06–0.07 mm) (0.065 ± 0.007, n = 2), 3 times as long as the preceding one (0.02–0.03 mm) (0.25 ± 0.007, n = 2). ***Thorax*.** Pronotum longer (1.14–1.15 mm) 1.45 ± 0.007 mm, n = 2) than wide (0.90–0.93 mm) (0.915 ± 0.021 mm, n = 2) (Fig. 32D); integument chagreened, punctures 3 times as long as eye facets and separated by approximately 2 punctured diameters, each puncture bearing a yellow-brown seta, disc convex, weakly elevated dorsally, forming a small depression in the basal part of each side, posterior margin almost straight with middle notch, sides almost straight, anterior angles rounded and posterior angles acute; mesosternal suture complete; scutellum with posterior margin rounded; each elytron 4.1 times as long (2.32–2.68 mm) (2.5 ± 0.254 mm, n = 2) as wide (0.62–0.64 mm) (0.63 ± 0.014 mm, n = 2), convex, without longitudinal costae, elytral apex rounded; hind wings with posterior radial vein (RP) length 3.2 times less than the length of MP1+2, radial cell closed, r3 vein absent, r4 vein reduced (not reaching the RP or the radial cell), those of the anterior anal and posterior anal sectors, evident (Fig. 32E). Legs: tarsomeres 1 and 2 of the prothoracic legs with a similar length and tarsomere 1 of meso- and metathoracic legs is longer than 2. ***Abdomen*.** Integument shiny, punctured, with long dense setae, sternite 7 with margin sinuate, sternite 8 with margin notched; aedeagus with three teeth at the inner apex of paramere (Fig. 32F–H).

#### Female and immatures.

Unknown.

#### Distribution.

Guatemala: Puerta Parada (Fig. 19).

### Cenophengus zuritai

Taxon classificationAnimaliaColeopteraPhengodidae

Vega-Badillo, Morrone & Zaragoza-Caballero sp. nov.

6DFFF2DB-8025-51A9-BF43-924CE28D9E51

http://zoobank.org/D2470925-DD08-4C4F-B97C-E736CD50185B

Fig. 33A–H

#### Type locality.

Cartago, Costa Rica.

#### Type material examined.

***Holotype*** ♂: “COSTA RICA: Cartago/ 4 km NE Canon Genesis II/9.761°N, 83.916°W/ FEB-MAR 1993, 2350 m/S.& P. Friedman. Malaise” “From the Michael Ivie Collection” |. ***Paratype*** ♂: “COSTA RICA: Cartago / 4 km NE Canon Genesis II/ 9.761°N, 83.916°W/ FEB-MAR 1993, 2350 m/ S.& P. Friedman. Malaise” “From the Michael Ivie Collection” (2) | NMNH.

#### Remarks.

*Cenophenguszuritai* is morphologically similar to *C.xiinbali*, but can be distinguished by the interocular distance and terminal maxillary palpomere. In *C.zuritai*, the interocular distance is 3 times eye width, whereas in *C.xiinbali*, it is 2.5 times longer. The terminal maxillary palpomere is shorter than the preceding three combined in *C.zuritai*, whereas in *C.xiinbali*, it is as long as the preceding three combined.

#### Diagnosis.

Head orange-brown, pronotum orange, integument chagreened, head a little wider than the pronotum, antennae long, more than twice the length of pronotum; antennal rami 1.5 times the respective antennomere, terminal maxillary palpomere shorter than the preceding three combined and each elytron 1.8 times as long as wide; aedeagus with one spine at the inner apex of paramere.

#### Description.

**Figure 33.**
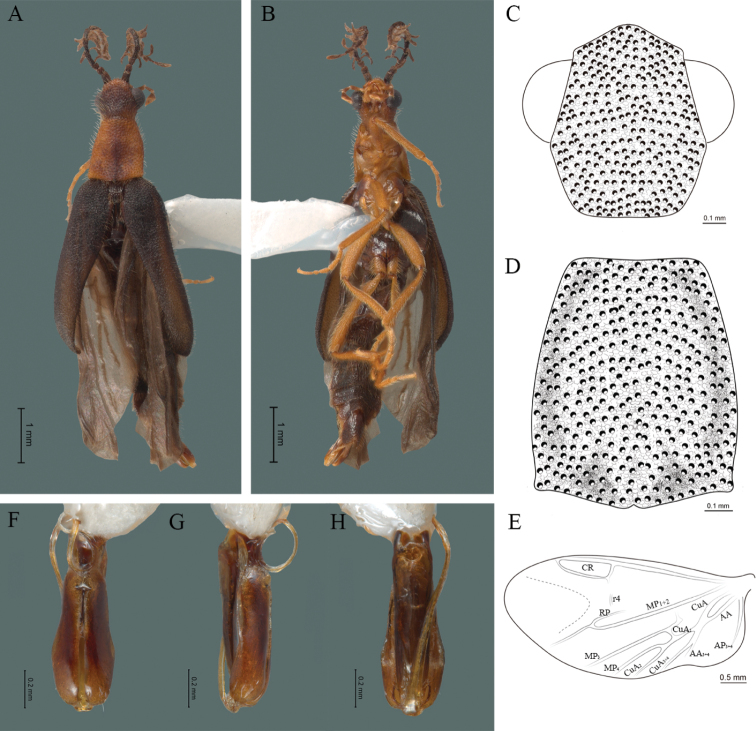
*Cenophenguszuritai* Vega-Badillo, Morrone & Zaragoza-Caballero, sp. nov., male. Habitus: **A** dorsal **B** ventral **C** head dorsal **D** pronotum **E** hind wing. Wing venation: CR = Radial Cell; r4 = radial 4 vein; RP = Posterior Radial vein; MP1+2 = Posterior Median vein; CuA = Cubital vein; AA and AP = Anterior and Posterior Anal veins. Aedeagus: **F** dorsal view **G** lateral view **H** ventral view.

**Male.** Body length 8.50–8.70 mm, maximum body width 0.86–0.88 mm (pronotum). Head orange-brown; antennae black to brown, pronotum orange; legs yellow to brown and two last sternites yellowish-coloured (Fig. 33A, B). ***Head*.** Wider (0.90–1.10 mm) (1.0 ± 0.1 mm, n = 3) than long (0.65–0.67 mm) (0.65 ± 0.011 mm, n = 3) (Fig. 33C), at eye level, a little wider than the pronotum, integument chagreened, punctures twice as long as eye facets and separated by approximately 0.5 punctured diameters, each puncture bearing a yellow-brown seta; interantennal distance (0.09–0.10 mm) (0.093 ± 0.005 mm, n = 3) wider than the length of antennomere 1 (0.19–0.20 mm) (0.196 ± 0.005 mm, n = 3); eyes 1/2 as long as head in lateral view, longer (0.33–0.35 mm) (0.343 ± 0.011 mm, n = 3) than wide (0.19–0.21 mm) (0.20 ± 0.1 mm, n = 3); interocular distance (0.60–0.63 mm) (0.613 ± 0.015 mm, n = 3) 3 times eye width; antennae long (2.40–2.48 mm) (2.426 ± 0.046 mm, n = 3) more than twice the length of pronotum; antennomere 1 (0.19–0.20 mm) (0.196 ± 0.005 mm, n = 3) longer than next two combined, antennomere 3 cup-shaped, 4 to 11 about equal in length (0.22–0.23 mm) (0.223 ± 0.005 mm, n = 3), 12 (terminal) (0.27–0.28 mm) (0.273 ± 0.005 mm, n = 3), antennal rami lanceolate in lateral view, 1.5 times the respective antennomere; terminal maxillary palpomere robust, securiform (0.30–0.33 mm) (0.31 ± 0.017 mm, n = 3), shorter than the preceding three combined; terminal labial palpomere spindle-shaped (0.10–0.11 mm) (0.103 ± 0.005 mm, n = 3), twice as long as the preceding one (0.05–0.06 mm) (0.053 ± 0.005 mm, n = 3). ***Thorax*.** Pronotum longer (1.10–1.15 mm) (1.116 ± 0.028 mm, n = 3) than wide (0.86–0.88 mm) (0.866 ± 0.011 mm, n = 3) (Fig. 33D); integument chagreened, punctures twice as long as eye facets and separated by approximately 1 punctured diameter, each puncture bearing a yellow-brown seta, disc convex, weakly elevated dorsally, forming a small depression in the basal part of each side, posterior margin and sides almost straight, anterior and posterior angles rounded; mesosternal suture complete; scutellum with posterior margin rounded; each elytron 1.8 times as long (2.8–3.0 mm) (2.86 ± 0.115 mm, n = 3) as wide (1.50–1.70 mm) (1.56 ± 0.115 mm, n = 3), convex, without longitudinal costae, elytral apex rounded; hind wings with posterior radial vein (RP) length 3.8 times less than the length of MP1+2, radial cell closed, r3 vein absent, r4 vein reduced (not reaching the RP or the radial cell), those of the anterior anal and posterior anal sectors, evident (Fig. 33E). Legs: tarsomeres 1 and 2 of pro- and mesothoracic legs with a similar length, tarsomere 1 of meathoracic legs is longer than 2. ***Abdomen*.** Integument shiny, punctured, with long dense setae, sternite 7 with margin sinuate, sternite 8 with margin notched; aedeagus with one spine at the inner apex of paramere (Fig. 33F–H).

#### Female and immatures.

Unknown.

#### Distribution.

Costa Rica: Cartago (Fig. 19).

#### Etymology.

Species dedicated to our dear friend and colleague Dr. Martín Leonel Zurita García, entomologist who dedicated his life to the study of beetles.

## Discussion

The genera *Cenophengus* and *Phengodes* (Zaragoza-Caballero and Pérez-Hernández 2014) are the richest genera within the family Phengodidae. *Cenophengus* species are distributed in the Nearctic and Neotropical regions, mainly in the mountainous areas of the Mexican Transition Zone (sensu Morrone et al. 2017), where its highest diversity is found (Vega-Badillo et al. 2021b). In this work, we describe four new species, three of which are distributed in the mountainous areas of Central America (Chiapas Highlands and Central American biogeographic provinces). This suggests that these species may show high levels of endemism, perhaps due to low vagility caused by the presence of neotenic females which reduces the capabilities to disperse and colonise new habitats (Bocak et al. 2008).

Some intraspecific variation in colouration has been observed in some species of *Cenophengus*, particularly in *C.pedregalensis*, *C.dedilis* and *C.major*, which are amongst the few species that have been widely collected, compared to other species for which only the holotype is available. One of the insights derived from observation of several populations of *C.pedregalensis* and *C.major*, however, is that, despite variation in colouration, wing venation remains comparatively constant intraspecifically, being useful for discerning between species within the genus (Vega-Badillo et al. 2021a). These observations are amenable to a morphometric analysis in the future, including extensive taxonomic and population sampling, as well as analysis of variation both in other structures and at the genetic level.

Taxonomy is essential for biodiversity knowledge and a crucial part of ecosystem conservation. It is necessary to implement an adequate sampling programme to explore the distributional patterns of *Cenophengus* species and detect the existence of possible areas of endemism. These could contribute to the identification of areas suitable for biodiversity conservation.

## Supplementary Material

XML Treatment for Cenophengus

XML Treatment for Cenophengus debilis

XML Treatment for Cenophengus baios

XML Treatment for Cenophengus brunneus

XML Treatment for Cenophengus ciceroi

XML Treatment for Cenophengus cuicatlaensis

XML Treatment for Cenophengus gardunoi

XML Treatment for Cenophengus gorhami

XML Treatment for Cenophengus hnogamui

XML Treatment for Cenophengus howdeni

XML Treatment for Cenophengus huatulcoensis

XML Treatment for Cenophengus kikapu

XML Treatment for Cenophengus longicollis

XML Treatment for Cenophengus magnus

XML Treatment for Cenophengus major

XML Treatment for Cenophengus marmoratus

XML Treatment for Cenophengus mboi

XML Treatment for Cenophengus mumui

XML Treatment for Cenophengus munizi

XML Treatment for Cenophengus niger

XML Treatment for Cenophengus pallidus

XML Treatment for Cenophengus pedregalensis

XML Treatment for Cenophengus punctatissimus

XML Treatment for Cenophegus saasil

XML Treatment for Cenophengus sonoraensis

XML Treatment for Cenophengus tsiik

XML Treatment for Cenophengus tupae

XML Treatment for Cenophengus villae

XML Treatment for Cenophengus wittmeri

XML Treatment for Cenophengus xiinbali

XML Treatment for Cenophengus zuritai
